# Contrasting genome composition and codon usage in *Listeria monocytogenes* temperate versus virulent phages

**DOI:** 10.1093/ve/veaf100

**Published:** 2025-12-18

**Authors:** Saba Kobakhidze, Anchao Song, Yi-Wei Tang, Mamuka Kotetishvili

**Affiliations:** One Health Institute, School of Science and Technology, the University of Georgia, 77a M. Kostava St., Tbilisi 0171, Georgia; College of Public Health, Chongqing Medical University, 61 University Town Middle Road, Chongqing 401331, China; College of Public Health, Chongqing Medical University, 61 University Town Middle Road, Chongqing 401331, China; One Health Institute, School of Science and Technology, the University of Georgia, 77a M. Kostava St., Tbilisi 0171, Georgia

**Keywords:** temperate phage, virulent phage, codon usage, *Listeria monocytogenes*, GC content, host–phage coevolution, viral adaptation

## Abstract

Phage lifestyles impose distinct selective pressures on genome architecture and codon usage. This study compares gene length and codon usage patterns in *Listeria monocytogenes* phages, analysing 686 temperate and 1516 virulent phages coding sequences. Most temperate phages had significantly smaller genomes (41 022.5 ± 3225.2 kb) and shorter genes (555 ± 647.1 *nt*) than virulent phages (101 089.3 ± 44 030.4 kb; 629 ± 583.5 *nt*). Codon adaptation was higher in the temperate phages [codon adaptation index (CAI) = 0.711 ± 0.045 vs. 0.679 ± 0.047; *P* < 1.4 × 10^−51^], while codon usage bias was stronger in the virulent phages (effective number of codons = 44.80 ± 5.863 vs. 46.00 ± 6.647; *P* < 1.0 × 10^−7^), which also had higher guanine and cytosine (GC) content (35.84 ± 2.985% vs. 35.21 ± 3.791%). CAI and %GC3 correlated strongly (*r* = −0.77 to −0.97) genome-wide. These results demonstrate that *L. monocytogenes* phage lifestyle shapes codon usage through distinct strategies of host adaptation and translational optimization. Also importantly, these findings can advance the development of biotechnological and synthetic biology tools for improving rapid determination of lifestyle of phages and their use in therapeutic and biocontrol applications.

## Introduction

Bacteriophages (phages) are viruses that infect and replicate within bacterial hosts, exerting profound influence on their ecology, population dynamics, and genome evolution (Holtappels et al. 2023). Phages shape microbial diversity and function in ecosystems by controlling bacterial abundance, promoting coevolution, and driving microbial community turnover (Koskella et al. 2022). Phages exhibit two principal lifestyles: a temperate (lysogenic) cycle, in which the phage genome integrates into the bacterial chromosome and can persist in a dormant state, and a virulent (lytic) cycle, in which infection rapidly leads to host cell lysis and release of progeny virions (Zhang et al. 2024). These distinct lifestyle strategies impose fundamentally different selective pressures on phage genomes, influencing not only their architecture and coding capacity, but also their adaptation to host translational machinery (Bailly-Bechet et al. 2007, Carbone 2008, Bobay et al. 2013). Like other viruses, phages rely on their host’s cellular machinery—specifically the translation apparatus—for reproduction, with codon usage biases towards translation optimization that can operate across the genomes of these organisms (Krakauer and Jansen 2002, Bailly-Bechet et al. 2007).

Codon usage bias—the non-random use of synonymous codons—can reflect a balance between mutational biases, genomic base composition, and selection for translational efficiency or accuracy (Pan et al. 1998, Bailly-Bechet et al. 2007). It was shown that synonymous codon usage patterns in temperate phages align more closely with those of the host genes, when compared to the same characteristics of the virulent phages, suggesting their selective advantage during prolonged intracellular association (Kunisawa et al. 1998). However, compared to temperate phages, certain virulent phages demonstrated higher codon usage biases, while exhibiting important compositional differences with respect to their host genome (Bailly-Bechet et al. 2007). When examining specific *Escherichia coli* and *Staphylococcus aureus* virulent phages (Prabhakaran et al. 2015), it was only suggested that, compared to their temperate counterparts, they could have better codon adaptation and stronger efficient translation. Collectively, these limited findings underscore the great need for more comprehensive investigations into genome-wide codon usage differences that can exist between temperate and virulent phages, and into their implications in host–phage coevolution and viral adaptation.

Among Gram-positive bacteria, *Listeria monocytogenes* remains one of the major foodborne pathogens, whose interactions with phages can carry significant clinical versus biotechnological implications: coevolution with temperate phages significantly contributes to its genetic divergence (Kobakhidze et al. 2024) and virulence, the latter being modulated by active prophages (Rabinovich et al. 2012). In contrast, virulent phages represent a promising technological approach for controlling *L. monocytogenes* across various food matrices (Perera et al. 2015, Lee et al. 2017, Kawacka et al. 2020) and related environments (Gupta and Adhikari 2022). In this light, however, it remains unclear whether temperate and virulent phages that infect *L. monocytogenes* differ in their genome-wide codon usage strategies—differences that could influence their respective patterns of adaptation and coevolution with the host.

This study presents the first genome-wide analysis of gene length and codon usage patterns in *L. monocytogenes* temperate and virulent tailed phages. We found that the temperate phages have smaller genomes and shorter genes, exhibiting significantly higher codon adaptation, which can suggest their long-term host integration and translational co-adaptation during lysogeny. In contrast, the virulent phages possess larger genomes, longer genes, illuminating stronger codon usage bias, likely shaped by selective pressures for rapid protein synthesis during lytic replication. Functional analysis of replication, lysis, and structural genes revealed lifestyle-specific codon usage trends, particularly involving GC content and GC3-driven biases. These findings suggest that phage lifestyle fundamentally shapes genome composition and translational strategies, with broader implications for our better understanding of phage–host co-evolution and viral fitness. Furthermore, they can contribute significantly to advancing the rational design of phage-based therapeutic and biocontrol applications, as well as biomarkers for a rapid determination of phage lifestyles.

## Materials and methods

### Genomes of temperate and virulent phages

The genomes of 10 temperate and 10 virulent tailed phages of *L. monocytogenes* (*Listeria* phages) from *Caudoviricetes* were selected. The corresponding DNA sequences of the genomes of these phages are publicly available in the GenBank nucleotide database maintained by the National Center for Biotechnology Information (NCBI; https://www.ncbi.nlm.nih.gov/). The genomes of these phages were randomly selected from the database above, with attention to variations in their lifestyle and genetic background—the latter assessed using the megablast or discontiguous megablast algorithms within the NCBI GenBank database. Importantly, the lifestyles of all selected phages (temperate or virulent) were experimentally determined and confirmed in previously published, peer-reviewed studies.

### Codon usage analysis of phage genomes

The protein-coding genes from the genomes of the temperate and virulent *Listeria* phages were analysed to assess codon usage patterns. Codon usage frequencies (totalling 1 510 127 codons) were calculated using 4518 protein-coding sequences (CDSs) of *L. monocytogenes* available in the Kazusa Codon Usage Database (https://www.kazusa.or.jp/codon/cgi-bin/showcodon.cgi?species=1639). Using the CAIcal server (http://genomes.urv.es/CAIcal/), we computed the following metrics for each gene: guanine and cytosine content (%GC), position-specific GC content (%GC1, %GC2, %GC3), *nt*, codon adaptation index (CAI), and effective number of codons (*Nc*). Codon usage matrices were generated for 686 genes from 10 temperate phages and 1516 genes from 10 virulent phages. For each dataset, we calculated the minimum, maximum, median, and standard deviation (SD) for all the variables. Pearson’s correlation coefficient (*r*) was used to evaluate the strength and direction of associations among CAIcal-derived variables within each lifestyle group (temperate vs. virulent). When applicable, correlation matrices were visualized as annotated heatmaps using the Seaborn data visualization package (Waskom 2021). Data wrangling and statistical analysis were performed in Python (v3.10) using the pandas library for structured data manipulation, following the approach described elsewhere (McKinney 2010). Genome size data (in kilobases) were grouped according to phage lifestyle, and the means and SDs were calculated for each group. Considering unequal variances between groups, Welch’s *t*-test was preferred, and thus chosen over the Student’s *t*-test to assess the observed differences in variance between the two groups.

### Principal component analysis

To explore differences in codon usage variation across the *Listeria* temperate and virulent phage genomes, we conducted principal component analysis (PCA) using the codon usage metrics (*nt*, CAI, *Nc*, %GC, %GC1, %GC2, %GC3) derived from the CAIcal analyses of the CDSs. Prior to PCA, all variables were standardized (z-transformed) to a mean of zero and unit variance to ensure comparability and equal weighting. PCA was performed using the scikit-learn library in Python (Pedregosa et al. 2011), applying eigen decomposition of the covariance matrix to extract orthogonal principal components. The first two components (PC1 and PC2), which captured the majority of variance, were retained for visualization. A PCA biplot was constructed to project each gene in PC1–PC2 space, with loading vectors indicating the contribution of each codon usage variable to the principal axes. The Mann–Whitney U test was used to statistically compare PC1 and PC2 distributions between the temperate and virulent phages, accommodating non-normality and unequal group sizes. All these analyses and visualizations were conducted using Python 3.11 with the pandas, matplotlib, and seaborn libraries.

## Results and discussion

Here, we delineate the genome architecture of the *L. monocytogenes* temperate and virulent tailed phages from *Caudoviricetes* by determining and correlating the genome-wide coding sequences (CDSs) length and codon usage patterns. The selected phage genomes showed genetic variations within both the temperate (Supplementary Table S1) and virulent (Supplementary Table S2) lifestyle groups. As shown, these genetic variations exhibited 73.54%–98.6% DNA identities across >1%–91% query coverages within the temperate lifestyle group, and 0%–98.69% DNA identities across 0%–100% query coverages within the virulent lifestyle group. A total of 686 CDSs from the temperate phages and 1516 CDSs from the virulent phages were examined. The key variables including gene length (*nt*), GC content (overall and by codon position), CAI, and effective number of codons (*Nc*) were determined to gain important insights into lifestyle-dependent genomic and translational strategies in the protein-coding genes of the above *Listeria* tailed phages. Supplementary Table S3 summarizes the information on strain designation, a GenBank accession number, host, genome size, and lifestyle for each phage examined in the study. Supplementary Tables S4 and S5 show the detailed CDSs *nt*, %GC, CAI, and *Nc* estimates obtained from our analyses for the genomes of the *Listeria* temperate and virulent phages, respectively.

### Protein-coding sequence length and codon usage genome-wide differences between *Listeria* temperate and virulent phages

The comparison of the full-genome lengths revealed a striking and statistically significant difference between the two *Listeria* phage lifestyle groups: a great majority of the temperate phages had a considerably smaller average genome size (41 022.5 ± 3225.2 kb) compared to their virulent counterparts (101 089.3 ± 44 030.4 kb). Welch’s *t*-test confirmed the robustness of this difference (*t* = −4.30, *P* = .001), strongly indicating a lifestyle-dependent divergence in genome complexity. Consistent with this trend, the virulent phages harboured mainly significantly longer genes (*nt* mean = 629) than the temperate phages (*nt* mean = 555) (Table 1), a difference supported by the Mann–Whitney U test (*U* = 452 826, *P* = 1.17 × 10^−6^) (Table 2). The broader *nt* variations observed within both groups [SDs of 583 and 647 for the virulent and temperate phages, respectively (Table 1)] underscore the heterogeneity of gene functions within each lifestyle, also suggesting differing coding demands. It remains to be determined whether the longer gene lengths observed in virulent phages reflect, in part, the demands of complex virion assembly and rapid replication in host cells.

**Table 1:** The descriptive statistics for gene length, codon adaptation, GC content at codon positions, and codon usage bias across the genomes of temperate versus virulent phages

Lifestyle		*nt*	CAI	%GC	%GC1	%GC2	%GC3	*Nc*
Temperate	Mean	555.3	0.71	35.21	44.62	31.47	29.54	46.00
	SD	647.1	0.045	3.791	5.745	5.782	5.88	6.64
	Minimum	66	0.51	22.4	21.8	16.7	12.5	25.1
	Median	384	0.71	35.4	45.2	31.2	29.2	46
	Maximum	5 385	0.87	45.5	60.5	46.9	59.1	61
							
	Mean	629.0	0.67	35.84	45.50	31.72	30.30	44.80
Virulent	SD	583.5	0.047	2.985	5.417	5.394	5.659	5.863
	Minimum	96	0.51	24.9	20.9	15	13.9	28.1
	Median	426	0.68	35.9	45.9	31.1	29.7	44.4
	Maximum	3 930	0.84	48.4	66.7	54.8	54.5	61

**Table 2:** Mann–Whitney U test-driven comparisons of the mean matrices of the gene length, codon adaptation, GC content by codon position, and codon usage bias between the *Listeria monocytogenes* temperate and virulent phages

**Genomic feature**	**Temperate** **(Mean)**	**Virulent** **(Mean)**	***U*-statistic**	***P*-value**
Gene size	555.3	629.0	452826.5	1.17E-06
CAI	0.71	0.67	728 751	1.41E-51
%GC	35.2	35.8	473 409	.0007
%GC1	44.6	45.5	478191.5	.002
%GC2	31.4	31.7	516015.5	.773
%GC3	29.5	30.3	481 115	.004
Nc	46.00	44.80	593 517	1.03E-07

**Figure 1:**
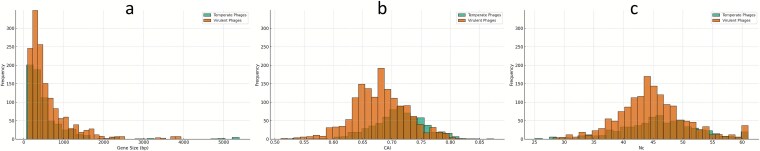
Frequency distribution of gene length (size in base pairs), CAI, and *Nc* determined for the protein-coding regions of the genomes of the *Listeria monocytogenes* temperate and virulent phages; (a) gene length distribution patterns for the genomes of the *L. monocytogenes* virulent and temperate phages; (b) CAI-parameter distribution patterns for the genomes of the *L. monocytogenes* virulent and temperate phages; (c) *Nc*-parameter distribution patterns for the genomes of the *L. monocytogenes* virulent and temperate phages.

More specifically, in the temperate phages, the gene lengths ranged from 66 to 5385 base pairs (Supplementary Table S4), with a mean of ~555 bp and a median of 399 bp, reflecting a strong bias towards shorter coding sequences (Table 1). By contrast, the virulent phages showed the gene lengths ranging from 63 to 6123 bp (Supplementary Table S5), with a mean of ~680 bp and a median of 531 bp (Table 1). The broader range in the gene size distribution, observed in the *Listeria* virulent phages versus their temperate counterparts (Fig. 1a), can partially reflect differences in their genome organization, lifestyle strategy, or functional specialization. The broader genome size range observed among virulent *Listeria* phages is similar to previous reports describing that virulent phages often exhibit genomes up to twice the size of those of temperate phages (Moura de Sousa et al. 2021).

In phages, genomic GC content—an approximate predictor of codon usage bias (Cardinale and Duffy 2011)—has been shown to correlate strongly with that of their hosts (Xia and Yuen 2005, Cardinale and Duffy 2011), with the genome of *L. monocytogenes* exhibiting a GC content of ~38.0% (Duceppe et al. 2017, Drali et al. 2019). In our study, the overall GC content was marginally higher across the genomes of the *Listeria* virulent phages (mean = 35.84%) than in those of their temperate counterparts (mean = 35.21%) (Table 1), with the difference reaching statistical significance (*U* = 473 409, *P* = 7.48 × 10^−4^) (Table 2). When partitioned by codon position, the significant differences were observed at GC1 and GC3 codon positions: GC1 was slightly elevated in the genes of the virulent phages (mean = 45.50%) compared to those of the temperate phages (mean = 44.62%) (*P* = .002), and GC3—the most variable position and a key marker of codon preference—was notably higher for the genes of the virulent phages (mean = 30.30%) relative to those of the temperate phages (mean = 29.54%) (*P* = .004) (Table 2). It is noteworthy that the mean %GC1 values (44.62 and 45.50) were higher than the corresponding mean %GC2 (31.48 and 31.72) and %GC3 (29.54 and 30.30) values calculated for the genes of the *Listeria* temperate and virulent phages, respectively (Table 1). These findings are in strong agreement with the tendency observed in microbial genes, where %GC1 is typically elevated relative to a GC content at the other codon positions (Hu et al. 2007).

**Figure 2:**
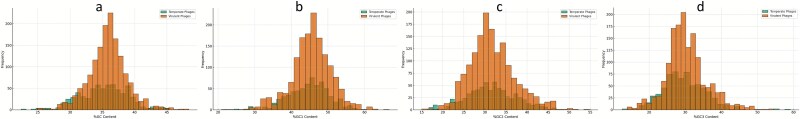
The distribution of GC content by codon position across the protein-coding genes of the *Listeria monocytogenes* temperate and virulent phages; (a) %GC-parameter distribution patterns across the protein-coding genes of the *L. monocytogenes* virulent and temperate phages; (b) %GC1-parameter distribution patterns across the protein-coding genes of the *L. monocytogenes* virulent and temperate phages; (c) %GC2-parameter distribution patterns across the protein-coding genes of the *L. monocytogenes* virulent and temperate phages; (d) %GC3-parameter distribution patterns across the protein-coding genes of the *L. monocytogenes* virulent and temperate phages.

Collectively, the above observed patterns suggest that the *Listeria* virulent phages experience distinct mutational selective pressures at synonymous codon sites, potentially influencing translational efficiency or replication kinetics. Across the both phage lifestyle groups, the GC content exhibited a unimodal, moderately skewed distribution (Fig. 2a–d): the virulent phages showed a broader and right-shifted distribution, indicating a higher and more variable GC content at the third codon position. Since GC3 is less constrained by amino acid identity (i.e. it is more synonymous), this suggests that codon usage bias is more relaxed or compositionally driven in these virulent phages.

The codon usage metrics, determined overall for the genes of the *Listeria* temperate and virulent phages, further highlighted their lifestyle-specific trends: the CAI-estimate, which reflects the extent to which genes utilize host-preferred codons (Jitobaom et al. 2020), was significantly higher for the genes of the temperate phages (mean = 0.711) than those of their virulent counterparts (mean = 0.679; *U* = 728 751, *P* = 1.41 × 10^−51^) (Tables 1 and 2 respectively). In this context, we propose that the *Listeria* temperate phages exhibit a closer adaptation to the codon usage of their bacterial host, which could be partially due to their long-term coexistence during lysogeny, a phase in which integration and maintenance within the host genome could promote translational optimization. Our observation aligns with the previous preliminary findings (Kunisawa et al. 1998), demonstrating a stronger correlation in synonymous codon usage between temperate phages and their hosts compared to virulent phages, suggesting a potential selective advantage during prolonged intracellular association. For the genes of the temperate phages, the CAI values formed a narrow, right-skewed distribution (Fig. 1b), concentrated between ~ 0.65 and 0.75, with a median of 0.714 and relatively low SD (SD = 0.046). For the genes of the virulent phages, the CAI values showed a broader distribution (Fig. 1b), skewed slightly towards lower values, with a median of 0.680 and a similar SD (SD = 0.047). These patterns, strongly supported statistically (*P* < .0001), suggest that the *Listeria* temperate phage genes are more consistently adapted to host codon preferences, whereas the genes of virulent phages exhibit greater variability in adaptation.

The analyses of gene populations in staphylococcal phages suggested that the highly biased synonymous codon usage, observed in certain loci, could be linked to enhanced adaptation to host cellular environments (Ge et al. 2020). In our study, as shown in Tables 1 and 2, respectively, the *Nc* estimate—a measure inversely related to codon usage bias (Wright 1990, Fuglsang 2004)—was higher for the genes in the *Listeria* temperate phages (*Nc* = 46.01) than in their virulent counterparts (*Nc* = 44.81; *U* = 593 517, *P* = 1.03 × 10^−7^). Generally, higher *Nc* values indicate a more relaxed codon selection regime, possibly reflecting the reduced translational pressure in the lysogenic phages, which can persist in a dormant state without the immediate need for rapid protein synthesis. This finding aligns with earlier work (Bailly-Bechet et al. 2007), which, although relying on a limited dataset of 37 phages across 15 hosts, reported greater codon usage bias in certain temperate phages compared to their virulent counterparts. However, it is important to note that *Nc*, which was previously thought to be independent of gene length (Wright 1990), has demonstrated both upward and downward correlations with *nt* of CDSs (Moriyama and Powell 1998, Khandia et al. 2022). Moreover, it was also shown that short sequences (~100–500 codons) can introduce statistical noise into *Nc* estimates (Supek and Vlahovicek 2005). This may partially explain our results, given the relatively higher abundance of the shorter genes found in the *Listeria* temperate phages compared to their virulent counterparts. Therefore, the above *Nc* values should be interpreted with caution, given their potential sensitivity to gene length. In our analyses, the derived *Nc* values, for the genes of the *Listeria* temperate phages, were normally distributed (Fig. 1c), centred around a mean of 46.0 with moderate spread (SD = 6.65), indicating relatively balanced codon usage across their genes. For the genes of the *Listeria* virulent phages, the analysis inferred a relatively left-shifted *Nc* distribution (Fig. 1c), with a lower mean (44.8) and smaller SD (SD = 5.86), suggesting more genes with strong codon usage bias. Thus, it can be thought that the *Listeria* virulent phages tend to restrict codon diversity (i.e. stronger bias), likely to enhance translation efficiency during rapid lytic cycles.

**Figure 3:**
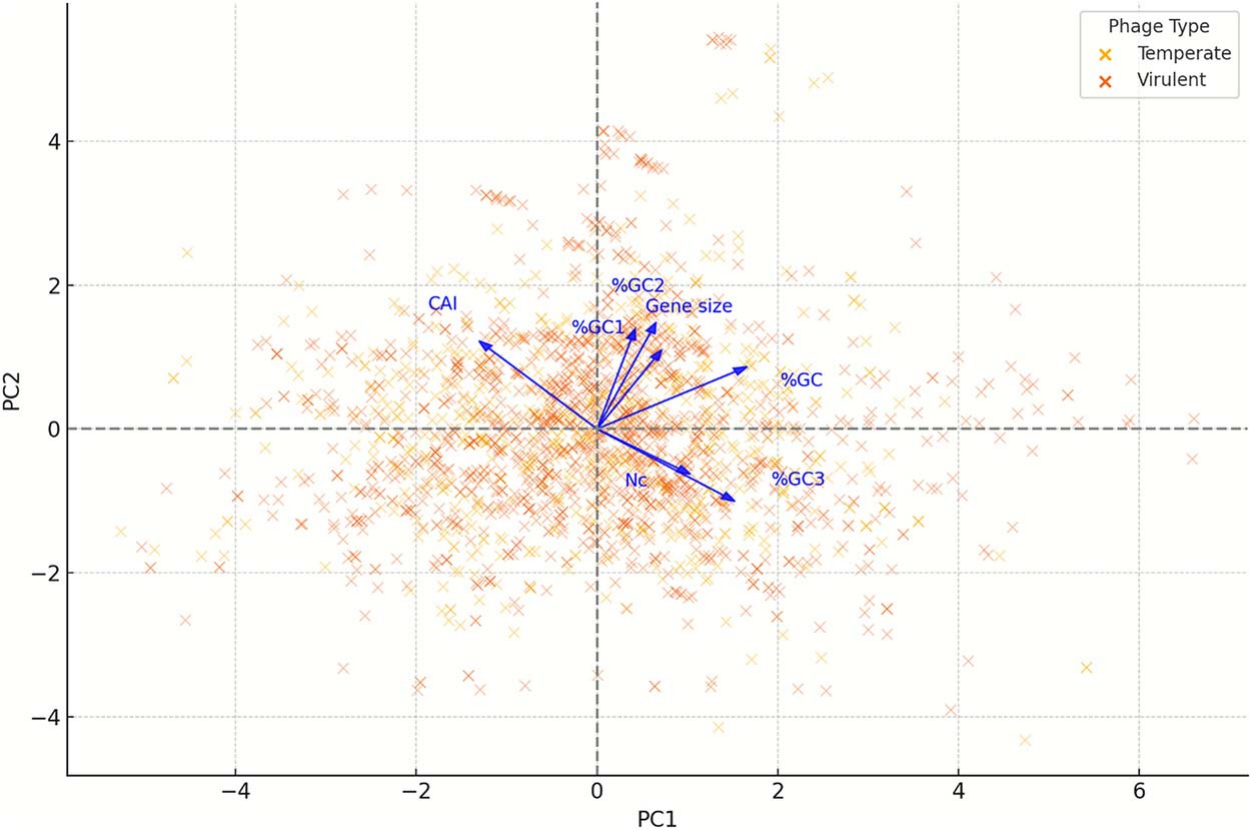
The PCA of gene features including the CDS length and codon usage metrics determined for the temperate and virulent phage genomes.

A PCA has been proven to be a powerful tool for uncovering patterns and dispersion in codon usage across the genomes of diverse organisms (Ge et al. 2020, Feng et al. 2022). In our study, the PCA was performed on the gene *nt* and codon usage metrics determined for the *Listeria* temperate and virulent phages, with PC1 and PC2 explaining 40.93% and 23.37% of the variance, respectively, accounting for 64.30% of the total variation in the dataset. As shown in the PCA biplot (Fig. 3), the analysis revealed partial overlap between the *Listeria* temperate and virulent phage genes in multivariate space, indicating no clear separation between the two lifestyles. However, specific directional trends were still evident: the virulent phage genes displayed greater dispersion along the positive PC1 axis, which was strongly influenced by %GC and %GC3 loadings; in contrast, the temperate phage genes tended to cluster slightly more towards the negative PC1 direction, where *Nc* and CAI exerted greater influence. Despite these tendencies, mean PC1 and PC2 scores were not significantly different between the two groups (PC1: *t* = 9.55 × 10^−16^, *P* ≈ 1.00; PC2: *t* = −3.11 × 10^−16^, *P* ≈ 1.00), suggesting that differences lie primarily in the pattern and spread of variation rather than in systematic shifts in central tendency. The greater dispersion of the virulent phage genes along PC1 (SD = 1.81 vs. 1.66 for temperate) suggests a broader range of their genome composition and codon usage bias, whereas the tighter clustering of the temperate phage genes could reflect more conserved codon profiles, potentially linked to prolonged association with host genomes during lysogeny. These observations provide additional strong support for our suggestion that the virulent phages exploit greater compositional flexibility, while the temperate phages maintain codon usage patterns more closely aligned with their hosts. Future studies integrating host genomic profiles, translational efficiency measures, and environmental data will be essential to determine whether these trends are driven primarily by host adaptation, replication strategy, or ecological constraints. Here, our findings show a notable parallel with the study on porcine circoviruses (Feng et al. 2022), despite the fundamental biological differences existing between bacterial phages and eukaryotic DNA viruses: in particular, the both studies suggest that viruses with lower codon adaptation to their host—such as the *Listeria* virulent phages in our dataset—can possibly gain selective advantages through increased genomic flexibility, potentially enhancing replication dynamics or facilitating immune evasion. We propose that the observed convergence towards reduced codon adaptation may reflect a broader viral strategy that offers advantages across diverse host–virus systems, rather than being confined to a particular virus type or host lineage. This interpretation aligns with the previous findings on certain polyvalent phages infecting *Salmonella* (Mondal et al. 2022) and *Staphylococcus* (El Haddad et al. 2014), which exhibited notable differences in codon usage compared to their hosts.

### Protein-coding sequence length and codon usage features of lysis, replication, and structural genes of *Listeria* temperate versus virulent phages

In the subsequent analyses, we specifically focused on exploring the CDSs *nt* and codon usage features specifically in the lysis, replication, and structural genes of these *Listeria* temperate and virulent phages. For this analysis, the lysis, replication, and structural genes were selected based on their annotated functional roles available in the GenBank records of each phage genome, inferred from sequence homology and standardized gene nomenclature. The descriptive statistics and independent *t*-tests revealed several significant differences in the gene characteristics between the two phage lifestyles, reflecting their distinct evolutionary and functional strategies. In particular, a marked difference in gene size was observed in the replication genes, with the genomes of the virulent phages encoding significantly longer CDSs (mean = 1044 bp) compared to those of the temperate phages (mean = 461 bp; *P* < .000001) (Table 3). This likely reflects functional adaptations in the virulent phages, which rely solely on lytic cycles and may require more complex or robust replication machinery. Although the lysis genes in the genomes of the virulent phages were also longer on average than those from the genomes of the temperate phages (Table 3), the difference was not statistically significant (*P* = .062) (Table 4). As also shown, codon adaptation was consistently higher in the temperate phages, especially for their replication (*P* < .0001) and structural genes (*P* < .000001). The higher CAI values suggest stronger translational optimization in the temperate phages, possibly facilitating efficient expression within the host during lysogeny. The lysis genes showed no significant difference in CAI between these two phage types, indicating that the both phage lifestyles may experience similar translational pressures for these genes.

**Table 3:** The descriptive mean statistics of length, codon usage, GC content by codon position, and codon usage bias for the lysis, replication, and structural genes of the *Listeria monocytogenes* temperate and virulent phages

**Lifestyle**	**Gene functional group**	**Gene size mean** **(SD)**	**CAI** **Mean** **(SD)**	**%GC** **Mean** **(SD)**	**%GC1** **Mean** **(SD)**	**%GC2** **Mean** **(SD)**	**%GC3** **Mean** **(SD)**	***Nc*** **Mean** **(SD)**
Temperate	Lysis	530.3 (269.68)	0.72 (0.0352)	35.50 (4.1907)	43.09 (3.8532)	33.64 (6.5863)	29.78 (6.7961)	49.18 (4.9271)
	Replication	461.7 (0.7056)	0.70 (0.0459)	36.47 (2.6961)	47.22 (4.1377)	31.69 (5.3688)	30.50 (4.7518)	47.18 (4.8348)
	Structural	1 017 (1076.6)	0.72 (0.0355)	36.86 (3.4812)	47.33 (5.1254)	34.14 (5.4891)	29.09 (4.8226)	47.37 (5.0730)
								
Virulent	Lysis	713.4 (328.47)	0.71 (0.0399)	38.38 (2.0841)	47.25 (3.7393)	38.02 (8.5134)	29.84 (5.2092)	43.90 (5.2855)
	Replication	1 044 (600.19)	0.66 (0.0333)	37.45 (2.1382)	49.14 (5.1824)	31.84 (4.8389)	31.36 (4.2398)	45.49 (3.8361)
	Structural	1 029 (828.98)	0.69 (0.0477)	37.84 (2.2725)	48.09 (3.4245)	35.66 (4.5345)	29.78 (5.5031)	44.29 (4.7652)

**Table 4:** Independent *t*-test comparisons of length, codon adaptation, GC content by codon position, and codon usage bias features determined for the lysis, replication, and structural genes of *Listeria monocytogenes* temperate and virulent phages

Functional group	Variable	Temperate (Mean)	Virulent (Mean)	*t*-statistic	*P*-value
Lysis genes	Gene size	530.3	713.4	−1.936	.062
	CAI	0.72	0.71	0.363	.718
	%GC	35.50	38.38	−3.070	.003
	%GC1	43.09	47.25	−3.578	.001
	%GC2	33.64	38.02	−1.815	.080
	%GC3	29.78	29.84	−0.034	.973
	*Nc*	49.18	43.9	3.338	.002
					
Replication genes	Gene size	461.7	1044.2	−7.186	1.88E-10
	CAI	0.70	0.66	4.105	.0001
	%GC	36.47	37.45	−1.853	.0690
	%GC1	47.22	49.14	−2.027	.0457
	%GC2	31.69	31.84	−0.141	.8877
	%GC3	30.50	31.36	−0.892	.3757
	*Nc*	47.18	45.49	1.798	.0773
					
Structural genes	Gene size	1 017	1 029	−0.106	.915
	CAI	0.72	0.69	6.820	4.93E-11
	%GC	36.86	37.84	−2.924	.003
	%GC1	47.33	48.09	−1.514	.131
	%GC2	34.14	35.66	−2.645	.008
	%GC3	29.09	29.78	−1.179	.239
	*Nc*	47.37	44.29	5.520	7.30E-08

Previous studies have reported that specific nucleotide usage patterns—namely, higher adenine and thymine (AT) content compared to GC content—in staphylococcal phages may enhance overall translation efficiency while minimizing energy expenditure, conserving chemical resources, and promoting elemental sparing in protein synthesis across the phage gene population (Ge et al. 2020). In our study, significant differences in the GC content were detected, particularly in the lysis genes: the virulent phage lysis genes had a higher overall GC content (mean = 38.3%) than those of their temperate counterparts (35.5%; *P* < .01) (Table 3), with the most notable increase at %GC1 (*P* < .001) (Table 4). The structural genes also exhibited the slightly higher GC content in the virulent phages, driven primarily by differences in the second codon position (*P* < .01). The *Nc* estimates calculated for the lysis, replication, and structural genes, revealed codon usage bias trends similar to those observed in the overall gene content of the *Listeria* temperate and virulent phages. Codon usage bias, assessed via these *Nc* estimates, was significantly stronger in the virulent phages for the lysis (*P* < .01) and structural genes (*P* < .000001) (Table 4). Although replication genes also showed a trend towards greater bias in the virulent phages, the difference was not statistically significant.

### Strength and direction of associations of protein-coding sequence length and codon usage patterns in *Listeria* temperate and virulent phages

**Figure 4:**
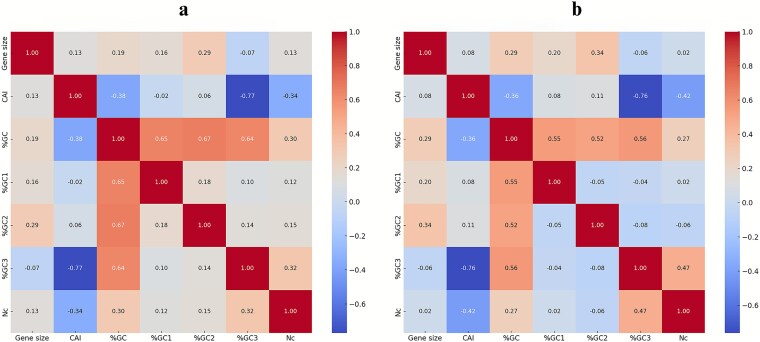
Pearson correlation heatmap of gene features including protein-coding sequence length, codon usage metrics, and GC content by codon position across the temperate and virulent phage genomes; (a) Pearson correlation heatmap of gene features determined for the temperate phage genomes; (b) Pearson correlation heatmap of gene features determined for the virulent phage genomes.

We evaluated the strength and direction of associations across the CDSs *nt* and codon usage matrices, for the genes of the *Listeria* temperate and virulent phages, calculating the respective *r*-values visualized further as the annotated heatmaps. The *r*-values calculated for the CDSs *nt* and codon usage patterns of the temperate and virulent phages are provided respectively in Supplementary Tables S6 and S7. We determined strong negative correlation (*r* = −0.77) between CAI and %GC3 (Fig. 4a), indicating that the highly optimized genes tend to use fewer GC-ending codons at the third codon position across the *Listeria* temperate phage genomes. In addition, moderate negative correlation (*r* = −0.38) was determined between CAI and overall %GC. These findings can suggest that the codon usage of these temperate phages is optimized for gene expression in relatively AT-rich hosts, e.g. such as *L. monocytogenes*. It was previously found that for prokaryotes e.g. GC content strongly correlates with the mean values for GC1, GC2, and GC3 (Zhou et al. 2014). Besides, it was also reported that the GC content of phages strongly correlates with the genomic GC content of their hosts (Simón et al. 2021). In our study, %GC1, %GC2, %GC3 were positively correlated with the overall %GC content, confirming that all these codon positions contribute to the genomic GC content of the above temperate phages. The CDS *nt* demonstrated weak positive correlation with %GC2 (*r* = 0.29), with the larger genes slightly favouring the GC content at the second codon position. As also shown in Fig. 4a, moderate negative correlation (*r* = −0.34) was observed among CAI and *Nc* for the genes of these *Listeria* temperate phages. This pattern mirrors findings from previous studies reporting negative correlations between CAI and *Nc* in genes from eukaryotic (Song et al. 2017, Li et al. 2022), prokaryotic (Nayak 2009, 2013), and viral sources (Hu et al. 2014).

A moderate negative correlation was found between the CAI and overall %GC values (*r* = −0.36), while a much stronger inverse relationship was observed between the former codon usage estimate and %GC3 pattern (*r* = −0.76) (Fig. 4b), suggesting selective pressure against GC-ending codons across the better codon-optimized genes in the *Listeria* virulent phages. Interestingly, similar negative correlations between CAI and %GC3 have been observed for genes from various AT-rich bacterial genomes (Ruden 2025). Here, we also show that all the three codon positions, %GC1 (*r* = 0.55), %GC2 (*r* = 0.52), and %GC3 (*r* = 0.56), contributed comparably to the overall GC content in the genes of the virulent phages. Furthermore, moderate negative correlation (*r* = −0.42) was observed among CAI and *Nc* in the analysis of the virulence phage genes as well, providing additional strong evidence suggesting that more optimized genes use fewer codons (i.e. exhibiting stronger codon usage bias).

**Figure 5:**
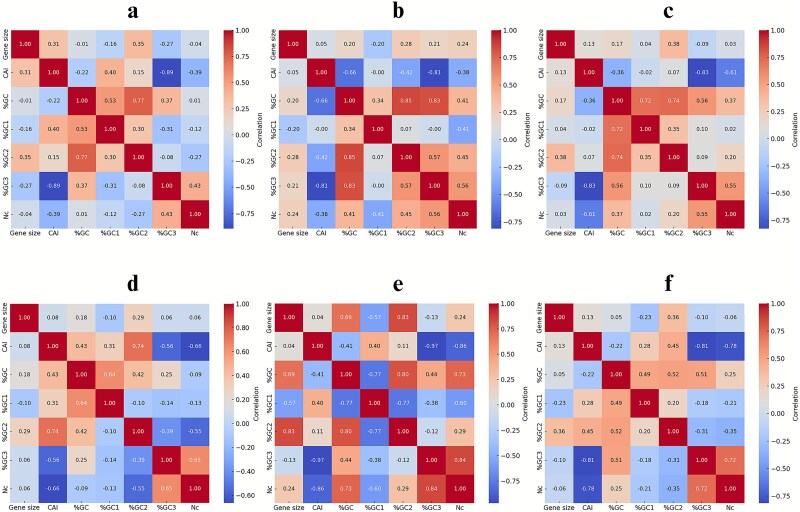
Pearson correlation heatmaps of coding sequence length, codon usage metrics, and GC content by codon position determined for the replication, lysis, and structural genes of the temperate and virulent phage genomes; (a) Pearson correlation heatmap of coding sequence length, codon usage metrics, and GC content by codon position determined for the replication genes of the temperate phage genomes; (b) Pearson correlation heatmap of coding sequence length, codon usage metrics, and GC content by codon position determined for the lysis genes of the temperate phage genomes; (c) Pearson correlation heatmap of coding sequence length, codon usage metrics, and GC content by codon position determined for the structural genes of the temperate phage genomes; (d) Pearson correlation heatmap of coding sequence length, codon usage metrics, and GC content by codon position determined for the replication genes of the virulent phage genomes; (e) Pearson correlation heatmap of coding sequence length, codon usage metrics, and GC content by codon position determined for the lysis genes of the virulent phage genomes; (f) Pearson correlation heatmap of coding sequence length, codon usage metrics, and GC content by codon position determined for the structural genes of the virulent phage genomes.

We also explored relationships across the gene *nt* and the above codon usage variables specifically for the replication, lysis, and structural genes in these phages. The annotated heatmaps shown in Fig. 5a–c (temperate phages) and Fig. 5d–f (virulent phages) summarize the derived *r-*values for replication gene metrics. As shown, a strong negative CAI–%GC3 correlation was observed across these genes of temperate phages (*r* = −0.89; Fig. 5a) compared to a moderate negative correlation determined for those of the virulent phages (*r* = −0.56; Fig. 5d). In contrast, *Nc*–%GC3 correlations were moderate and positive for the above loci of the temperate phages (*r* = 0.43), while being strong and positive for those of the virulent phages (*r* = 0.65). CAI–*Nc* correlations were negative for these genes in both groups, but this relationship was notably weaker in the replication genes of the temperate phages (*r* = −0.39) than in those of the virulent phages (*r* = −0.66). Overall %GC content correlated positively with %GC1 and %GC2 for the replication genes of the both phage types, but with differing patterns of strength. In the temperate phages, %GC–%GC1 correlations were moderate (*r* = 0.53) and %GC–%GC2 correlations were strong (*r* = 0.77) (Fig. 5a). In the virulent phages, the pattern was reversed, with a stronger %GC–%GC1 correlation (*r* = 0.64) and a weaker %GC–%GC2 correlation (*r* = 0.42) (Fig. 5d) for their replication genes. The markedly stronger negative CAI–%GC3 correlation for the above genes of the temperate phages suggests a greater inverse relationship between codon adaptation and GC3 composition, potentially reflecting genomic adaptation to their host codon preferences and compositional constraints. In contrast, these genes of the virulent phages display the stronger *Nc*–%GC3 and CAI–*Nc* correlations, suggesting a tighter link between codon bias and GC composition, possibly driven by selection for efficient translation during rapid replication cycles.

The analysis of the lysis genes from the temperate phages revealed a very strong negative correlation between CAI and %GC3 (*r* = −0.81), and a strong negative correlation between CAI and the overall %GC (*r* = −0.66) (Fig. 5c). In addition, there were very strong positive correlations observed between the overall %GC and %GC2 (*r* = 0.85), and between the overall %GC and %GC3 (*r* = 0.83), indicating that the GC content variation in these genes is strongly coupled between the second and third codon positions. Interestingly, the CAI–*Nc* relationship in the temperate phage lysis genes was weakly negative (*r* = −0.38), suggesting that codon usage (as measured by CAI) is only loosely linked to *Nc* in these phages. In contrast, the lysis genes from the virulent phages displayed a very strong negative CAI–*Nc* correlation (*r* = −0.86) (Fig. 5e), suggesting that at least for the above functional group of genes of these organisms, codon usage bias is much more tightly constrained by the available codon repertoire. These genes also exhibited a very strong positive *Nc*–%GC3 correlation (*r* = 0.84) and a strong positive *Nc*–%GC correlation (*r* = 0.73), indicating that GC content, especially at the third codon position, is a major determinant of codon diversity. In this context, it is noteworthy that studies examining *Nc*–%GC3 relationships have identified multimodal correlation patterns, which reflect diverse underlying selective and mutational pressures (Hassan et al. 2009, Saha et al. 2021), including the investigation on mycobacteriophage genomes illuminating a strong negative correlation between *Nc* and %GC3 (Hassan et al. 2009). For the lysis genes of the virulent phages, CAI showed an exceptionally strong negative correlation with %GC3 (*r* = −0.97), far stronger than observed in the respective genes of the temperate phages, suggesting a pronounced trade-off between codon adaptation and GC3 composition in these genomes.

Interestingly, in DNA viruses, GC content and gene length show a positive correlation, whereas in RNA viruses, the correlation is negative, suggesting that distinct mutational and selective pressures shape their genomic architectures (Chen 2013). Unlike the temperate phages, the CDSs *nt* of the lysis genes, in the virulent phages, was strongly correlated with %GC2 (*r* = 0.83) and %GC content (*r* = 0.69), implying that longer lysis genes may preferentially use GC-rich codons, especially at the second position in this group. A very strong negative correlation between CAI and %GC3 (*r* = −0.83) and a strong negative correlation between CAI and *Nc* (*r* = −0.61) were observed for the structural genes of the *Listeria* temperate phages. As shown in Fig. 5c, %GC displayed strong positive correlations with %GC1 (*r* = 0.72) and %GC2 (*r* = 0.74). Additionally, *Nc* was moderately and positively correlated with %GC3 (*r* = 0.55) for these temperate phage genes.

For the structural genes of the *Listeria* virulent phages, a similarly strong pattern was observed: CAI and %GC3 were very strongly negatively correlated (*r* = −0.81), and CAI and *Nc* showed a strong negative correlation (*r* = −0.78) (Fig. 5f). *Nc* was strongly and positively correlated with %GC3 (*r* = 0.72), while %GC showed moderate positive correlations with %GC3 (*r* = 0.51), %GC2 (*r* = 0.52), and %GC1 (*r* = 0.49) across these genes of virulent phages. The strong and consistent negative associations between CAI and %GC3, observed in both the temperate and virulent phage structural genes, suggest that selection for efficient translation drives codon usage at the expense of higher GC3 content, likely reflecting adaptation to host-specific translational machinery. The lower %GC3 values appear to be associated with higher codon adaptation, potentially reflecting selective pressures to match host tRNA pools or translational efficiency constraints. Similarly, the negative CAI–*Nc* correlations indicate that increased codon bias (lower *Nc*) accompanies higher CAI values, reinforcing the idea of translational optimization in phage structural proteins.

The classification of phage lifecycles is of great importance in deploying the potential applications of phage-based therapeutic or biotechnological tools. However, traditional biological methods for identifying phage lifecycles are complicated, time-consuming, and still lack the desired level of precision. Overall, the parallels between the *Listeria* temperate and virulent phages imply that despite differences in lifecycle strategies, similar genomic and codon usage constraints operate on their structural genes, possibly driven by common host interactions and translational demands. Nevertheless, our findings, which reveal significant differences in genome composition and codon usage between the above phage groups, hold strong potential for advancing biotechnology and synthetic biology tools. These insights can enable more rapid and precise determination of phage lifestyles and enhance the development of phage-based therapeutic and biocontrol applications.

## Conclusions

This study presents the first genome-wide comparison of gene architecture and codon usage between temperate and virulent *L. monocytogenes* phages, revealing distinct, lifestyle-dependent genomic strategies. The temperate phages generally exhibited smaller genomes, shorter genes, and significantly higher CAIs, suggesting stronger alignment with host-preferred codons—likely a result of prolonged lysogenic integration and translational co-adaptation. In contrast, the virulent phages mainly possessed larger genomes, longer genes, higher GC and GC3 content, and stronger overall codon usage bias, reflecting selective pressures for rapid and efficient gene expression during lytic replication. The virulent phages showed greater variation in codon usage and GC content, suggesting more flexible translational strategies, whereas temperate phages maintained more conserved codon profiles aligned with host preferences. These findings demonstrate that phage lifestyle significantly shapes genome composition and codon usage dynamics in *Listeria* phages. Beyond advancing our understanding of phage–host co-evolution, this work also lays the groundwork for codon usage–based biomarkers to rapidly distinguish phage lifestyles, while also enabling synthetic biology strategies to enhance phage-based therapeutics, diagnostics, and biocontrol.

## Supplementary Material

SUPPLEMENTARY_INFORMATION_2025_new_veaf100

## Data Availability

In this study, we analysed the whole-genome sequences of *Listeria* temperate and virulent phages. The above whole-genome sequences of these phages are publicly available in the NCBI GenBank database, and their respective accession numbers are provided in Supplementary Table S3 of this manuscript.
