# Supplementary material for: Contrasting genome composition and codon usage in Listeria monocytogenes temperate versus virulent phages
Source: Virus Evol. 2025 Dec 18;12(1):veaf100. doi: 10.1093/ve/veaf100 (PMC12822063; doi:10.1093/ve/veaf100)
Supplement: SUPPLEMENTARY_INFORMATION_2025_new_veaf100 [file supplementary_information_2025_new_veaf100.pdf]

SUPPLEMENTAL INFORMATION

Contrasting genome composition and codon usage in *Listeria monocytogenes* temperate versus virulent Phages

Table S1. The matrix of BLAST-derived query coverage and nucleotide identity values illustrating genome-wide similarity among the ten temperate phages.

Phage genome GenBank acc. #		NC_009810.1	NC_009815.1	NC_009812.1	NC_009813.1	NC_003291.2	NC_003216.1	NC_028929.1	NC_024387.1	NC_024384.1	NC_021539.2
NC_009810.1	Query Coverage (%)	-	7	1	2	6	48	65	2	75	8
	DNA Identity (%)	-	92.24	87.11	74.3	87.34	90.81	94.29	92.09	91.22	88.71
NC_009815.1	Query Coverage (%)	7	-	4	1	2	29	17	6	13	1
	DNA Identity (%)	92.24	-	96.91	85.54	88.74	98.26	95.67	92.59	92.24	87.05
NC_009812.1	Query Coverage (%)	1	4	-	4	26	4	1	59	1	28
	DNA Identity (%)	87.11	96.91	-	82.03	86.2	96.91	86.48	90.37	89.5	92.36
NC_009813.1	Query Coverage (%)	2	1	4	-	1	<1	1	<1	<1	2
	DNA Identity (%)	74.3	85.54	82.03	-	81.19	93.88	74.85	81.88	85.21	73.54
NC_003291.2	Query Coverage (%)	6	2	26	1	-	<1	6	35	9	91
	DNA Identity (%)	87.34	88.74	86.2	81.19	-	89.8	86.96	91.67	94.86	88.27
NC_003216.1	Query Coverage (%)	48	29	4	<1	<1	-	59	7	52	2
	DNA Identity (%)	90.81	98.26	96.91	93.88	89.8	-	89.11	92.33	90.46	83.28
NC_028929.1	Query Coverage (%)	65	17	1	1	6	59	-	4	79	6
	DNA Identity (%)	94.29	95.67	86.48	74.85	86.96	89.11	-	92.64	91.99	84.26
NC_024387.1	Query Coverage (%)	2	6	59	<1	35	7	4	-	1	29
	DNA Identity (%)	92.09	92.59	90.37	81.88	91.67	92.33	92.64	-	85.34	86.03
NC_024384.1	Query Coverage (%)	75	13	1	<1	9	52	79	1	-	7
	DNA Identity (%)	91.22	92.24	89.5	85.21	94.86	90.46	91.99	85.34	-	92.63
NC_021539.2	Query Coverage (%)	8	1	28	2	91	2	6	29	7	-
	DNA Identity (%)	88.71	87.05	92.36	73.54	88.27	83.28	84.26	86.03	92.63	-

Table S2. The matrix of BLAST-derived query coverage and nucleotide identity values illustrating genome-wide similarity among the ten virulent phages.

Phage genome GenBank acc. #		NC_009814.1	NC_011308.1	NC_009811.2	NC_007610.1	NC_018831.1	NC_024364.1	NC_041862.1	NC_024383.1	NC_024375.1	NC_024359.1
NC_009814.1	Query Coverage (%)	-	19	<1	<1	<1	<1	<1	<1	<1	<1
	DNA Identity (%)	-	65.99	70.19	73.08	75.59	72.31	73.08	71.43	75.59	71.43
NC_011308.1	Query Coverage (%)	19	-	<1	0	0	0	0	0	0	0
	DNA Identity (%)	65.99	-	92	0	0	0	0	0	0	0
NC_009811.2	Query Coverage (%)	<1	<1	-	98	1	95	98	98	1	96
	DNA Identity (%)	70.19	92	-	98.44	67.46	97.89	98.17	98.44	75.11	98.36
NC_007610.1	Query Coverage (%)	<1	0	98	-	<1	98	100	99	1	98
	DNA Identity (%)	73.08	0	98.44	-	77.83	98.07	98.61	98.69	91.43	98.33
NC_018831.1	Query Coverage (%)	<1	0	1	<1	-	1	1	1	95	2
	DNA Identity (%)	75.59	0	67.46	77.83	-	77.83	77.78	77.8	96.92	66.13
NC_024364.1	Query Coverage (%)	<1	0	95	98	1	-	98	98	1	98
	DNA Identity (%)	72.31	0	97.89	98.07	77.83	-	97.95	98.09	91.43	97.96
NC_041862.1	Query Coverage (%)	<1	0	98	100	1	98	-	99	97	1
	DNA Identity (%)	73.08	0	98.17	98.61	77.78	97.95	-	98.04	98.3	91.43
NC_024383.1	Query Coverage (%)	<1	0	98	99	1	98	99	-	1	96
	DNA Identity (%)	71.43	0	98.44	98.69	77.8	98.09	98.04	-	90.03	98.41
NC_024375.1	Query Coverage (%)	<1	0	1	1	95	1	97	1	-	2
	DNA Identity (%)	75.59	0	75.11	91.43	96.92	91.43	98.3	90.03	-	66.13
NC_024359.1	Query Coverage (%)	<1	0	96	98	2	98	1	96	2	-
	DNA Identity (%)	71.43	0	98.36	98.33	66.13	97.96	91.43	98.41	66.13	-

Table S3. The strain designations, GenBank accession numbers, host, genome size, and lifestyle for the bacteriophages examined in the study

Phage designation	GenBank acc. No.	Host	Genome length (Kb)	Phage Lifestyle	Supplemental references
Listeria phage A500	NC_009810.1	*Listeria monocytogenes*	38867	Temperate	Vu et al. Microorganisms. Jun 22;9(7):1354 (2021).[1]
Listeria phage A006	NC_009815.1	*Listeria monocytogenes*	38124	Temperate	Vu et al. Microorganisms. Jun 22;9(7):1354 (2021).[1]
Listeria phage B025	NC_009812.1	*Listeria monocytogenes*	42653	Temperate	Vu et al. Microorganisms. Jun 22;9(7):1354 (2021).[1]
Listeria phage B054	NC_009813.1	*Listeria monocytogenes*	48172	Temperate	Vu et al. Microorganisms. Jun 22;9(7):1354 (2021).[1]
Listeria Phage PSA	NC_003291.2	*Listeria monocytogenes*	37618	Temperate	Vu et al. Microorganisms. Jun 22;9(7):1354 (2021).[1]
Listeria phage A118	NC_003216.1	*Listeria monocytogenes*	40834	Temperate	Vu et al. Microorganisms. Jun 22;9(7):1354 (2021).[1]
Listeria phage vB_LmoS_293	NC_028929.1	*Listeria monocytogenes*	40759	Temperate	Vu et al. Microorganisms. Jun 22;9(7):1354 (2021).[1]
Listeria phage LP-101	NC_024387.1	*Listeria monocytogenes*	43767	Temperate	Vu et al. Microorganisms. Jun 22;9(7):1354 (2021).[1]
Listeria phage LP-030-3	NC_024384.1	*Listeria monocytogenes*	41156	Temperate	Vu et al. Microorganisms. Jun 22;9(7):1354 (2021).[1]
Listeria phage LP-030-2	NC_021539.2	*Listeria monocytogenes*	38275	Temperate	Vu et al. Microorganisms. Jun 22;9(7):1354 (2021).[1]
Listeria phage p35	NC_009814.1	*Listeria monocytogenes*	35822	Virulent	Dorscht et al. J Bacteriol. Dec;191(23):7206-15 (2009).[2]
Listeria phage p40	NC_011308.1	*Listeria monocytogenes*	35638	Virulent	Dorscht et al. J Bacteriol. Dec;191(23):7206-15 (2009).[2]
Listeria phage A511	NC_009811.2	*Listeria monocytogenes*	137619	Virulent	Habann et al. Mol Microbiol. Apr;92(1):84-99. (2014)[3]
Listeria phage p100	NC_007610.1	*Listeria monocytogenes*	131384	Virulent	Carlton et al. Regul Toxicol Pharmacol. Dec;43(3):301-12. (2005)[4]
Listeria phage p70	NC_018831.1	*Listeria monocytogenes*	67170	Virulent	Schmuki et al. J Virol. Dec;86(23):13099-102. (2012)[5]
Listeria phage List-36	NC_024364.1	*Listeria monocytogenes*	131952	Virulent	Perera et al. Food Microbiol. Dec;52:42-8. (2015)[6]
Listeria phage LP-064	NC_041862.1	*Listeria monocytogenes*	135279	Virulent	Hudson et al. Microbiol Resour Announc. Jan 7;10(1). (2021)[7]
Listeria phage LP-083-2	NC_024383.1	*Listeria monocytogenes*	135831	Virulent	Peters et al.. Microbiol Resour Announc. Nov 14;8(46):e01229-19. (2019)[8]
Listeria phage LP-026	NC_024375.1	*Listeria monocytogenes*	67150	Virulent	Denes et al. Appl Environ Microbiol. Aug;80(15):4616-25. (2014)[9]
Listeria phage LP-048	NC_024359.1	*Listeria monocytogenes*	133048	Virulent	Peters et al. Microbiol Resour Announc. Nov 14;8(46):e01229-19. (2019)8

Supplemental references

1. Vu HTK, Stasiewicz MJ, Benjakul S, Vongkamjan K. Genomic Analysis of Prophages Recovered from Listeria monocytogenes Lysogens Found in Seafood and Seafood-Related Environment. Microorganisms. Jun 22;9(7):1354 (2021).

2. Dorscht J, Klumpp J, Bielmann R, Schmelcher M, Born Y, Zimmer M, Calendar R, Loessner MJ. Comparative genome analysis of Listeria bacteriophages reveals extensive mosaicism, programmed translational frameshifting, and a novel prophage insertion site. J Bacteriol. Dec;191(23):7206-15 (2009).

3. Habann M, Leiman PG, Vandersteegen K, Van den Bossche A, Lavigne R, Shneider MM, Bielmann R, Eugster MR, Loessner MJ, Klumpp J. Listeria phage A511, a model for the contractile tail machineries of SPO1-related bacteriophages. Mol Microbiol. Apr;92(1):84-99. (2014)

4. Carlton RM, Noordman WH, Biswas B, de Meester ED, Loessner MJ. Bacteriophage P100 for control of Listeria monocytogenes in foods: genome sequence, bioinformatic analyses, oral toxicity study, and application. Regul Toxicol Pharmacol. Dec;43(3):301-12. (2005)

5. Schmuki MM, Erne D, Loessner MJ, Klumpp J. Bacteriophage P70: unique morphology and unrelatedness to other Listeria bacteriophages. J Virol. Dec;86(23):13099-102. (2012)

6. Perera MN, Abuladze T, Li M, Woolston J, Sulakvelidze A. Bacteriophage cocktail significantly reduces or eliminates Listeria monocytogenes contamination on lettuce, apples, cheese, smoked salmon and frozen foods. Food Microbiol. Dec;52:42-8. (2015)

7. Hudson LK, Peters TL, Bryan DW, Song Y, den Bakker HC, Denes TG. Complete Genome Sequences of Three Listeria monocytogenes Bacteriophage Propagation Strains. Microbiol Resour Announc. Jan 7;10(1). (2021)

8. Peters TL, Hudson LK, Song Y, Denes TG. Complete Genome Sequences of Two Listeria Phages of the Genus Pecentumvirus. Microbiol Resour Announc. Nov 14;8(46):e01229-19. (2019)

9. Denes T, Vongkamjan K, Ackermann HW, Moreno Switt AI, Wiedmann M, den Bakker HC. Comparative genomic and morphological analyses of Listeria phages isolated from farm environments. Appl Environ Microbiol. Aug;80(15):4616-25. (2014)

Table S4. The descriptive statistics of size, codon usage, GC content by codon position, and codon usage bias for the genes identified across the genomes of the Listeria temperate phages

No.	Gene	Gene coordinates in a genome	Gene length (nt)	CAI	%GC	%GC1	%GC2	%GC3	Nc
1	acetyltransferase	21345..21881	537	0.719	33.7	43	32.4	25.7	50.6
2	acetyltransferase	21345..21881	537	0.719	33.7	43	32.4	25.7	50.6
3	acetyltransferase	21307..21906	600	0.784	28.5	38.5	29	18	37.8
4	AcrIIA2_family_anti-CRISPR_protein	complement(22430..22780)	351	0.777	30.5	47	23.1	21.4	44.1
5	anti-CRISPR_protein_AcrIIA1	complement(21976..22425)	450	0.667	32	39.3	24	32.7	51.5
6	anti-CRISPR_protein_AcrIIA1	complement(20814..21263)	450	0.708	30	40.7	22.7	26.7	51.3
7	anti-CRISPR_protein_AcrIIA2	complement(21269..21640)	372	0.697	33.6	45.2	23.4	32.3	52.7
8	anti-CRISPR_protein_AcrIIA3	complement(21674..22051)	378	0.802	34.1	40.5	34.1	27.8	37.6
9	anti-repressor	28810..29586	777	0.725	33.7	44.4	29.3	27.4	45.3
10	anti-repressor	28810..29586	777	0.725	33.7	44.4	29.3	27.4	45.3
11	anti-repressor	37170..37949	780	0.711	34.9	45	30.4	29.2	45.2
12	anti-repressor	27786..28565	780	0.725	34.4	45	30.8	27.3	41.3
13	anti-repressor	29733..30521	789	0.737	35.4	46.8	31.2	28.1	45.5
14	anti-repressor_Ant	44372..45139	768	0.708	33.5	44.1	29.3	27	46.8
15	bacterioGp15_family_protein	8925..9527	603	0.718	35.3	44.8	27.9	33.3	47.5
16	bacterioGp15_family_protein	8925..9527	603	0.718	35.3	44.8	27.9	33.3	47.5
17	bacterioGp15_family_protein	8904..9506	603	0.729	34.8	45.8	27.9	30.8	49.1
18	bacterioGp15_family_protein	17318..17923	606	0.718	35.1	44.6	27.7	33.2	48
19	bacterioGp15_family_protein	8926..9531	606	0.733	34.8	46	27.7	30.7	49.3
20	baseplate_hub	17706..18512	807	0.726	34.7	40.9	40.9	22.3	47.2
21	baseplate_protein	20344..20985	642	0.717	36.4	47.2	36.9	25.2	50.4
22	baseplate_upper_protein	17417..18691	1275	0.717	35.6	42.1	36.9	27.8	49.5
23	baseplate_wedge_subunit	18848..19210	363	0.73	30.6	45.5	24.8	21.5	44.8
24	baseplate_wedge_subunit	19203..20354	1152	0.747	36.5	51.3	35.7	22.4	45.4
25	Bet-like_ssDNA_annealing_protein	33423..34208	786	0.729	36.6	50.4	34	25.6	44
26	BH0509_family_protein	38439..38579	141	0.661	31.2	40.4	17	36.2	42.7
27	carbohydrate-binding_protein_CenC	16812..17906	1095	0.715	39.1	44.7	42.5	30.1	49.1
28	Cas9_inhibitor_AcrIIA9_family_protein	38795..39217	423	0.688	37.4	44	33.3	34.8	54.4
29	cobalt_ABC_transporter_domain-containing_protein	complement(33287..34009)	723	0.689	33.1	43.6	29.9	25.7	47.4
30	distal_tail_protein_Dit	12830..13660	831	0.736	30.8	38.3	29.6	24.5	42.5
31	distal_tail_protein_Dit	12863..14512	1650	0.722	35.8	42.4	36.4	28.5	46.2
32	DNA_helicase	36858..37970	1113	0.713	36.2	46.9	33.2	28.6	49.3
33	DNA_helicase	35993..37135	1143	0.702	39.1	49.3	34.1	33.9	48
34	DNA_helicase	31119..32312	1194	0.756	35.2	46	33.2	26.4	44.2
35	DNA_helicase	30396..31652	1257	0.718	36.7	47.7	33.9	28.4	48.5
36	DNA_methyltransferase	34537..35001	465	0.65	37.4	41.3	32.3	38.7	54.9
37	DNA_methyltransferase	28601..29065	465	0.651	36.8	41.3	32.3	36.8	56.6
38	DNA_methyltransferase	28062..28592	531	0.644	35.6	40.7	31.1	35	48
39	DNA_methyltransferase	28316..29029	714	0.769	33.5	45.8	30.3	24.4	43.3
40	DNA_methyltransferase	24407..25219	813	0.71	36.5	46.9	32.5	30.3	49.2
41	DNA_methyltransferase	24130..24942	813	0.71	36.7	47.2	32.5	30.3	49.4
42	DNA_primase	32185..34458	2274	0.714	36.7	46.3	33.1	30.7	51.1
43	DNA_primase	38503..40776	2274	0.735	35.7	45.8	33.2	28	49.1
44	DNA_primase	32843..35116	2274	0.737	36.2	46.2	32.6	29.8	50.1
45	DNA_primase	37663..39936	2274	0.738	36	46.2	32.8	29	48.7
46	DnaC-like_helicase_loader	325..1131	807	0.715	35.1	40.9	34.9	29.4	45.5
47	DnaC-like_helicase_loader	35758..36573	816	0.705	34.9	45.2	30.5	29	48.1
48	DnaD-like_helicase_loader	32656..33573	918	0.643	38.1	45.8	32	36.6	53
49	DnaD-like_helicase_loader	32656..33573	918	0.643	38.1	45.8	32	36.6	53
50	DnaD-like_helicase_loader	33608..34540	933	0.674	35.2	42.8	29.6	33.1	50.4
51	DUF1642_domain-containing_protein	28109..28384	276	0.653	35.5	40.2	28.3	38	48.9
52	DUF1642_domain-containing_protein	29463..29996	534	0.681	39	48.9	33.7	34.3	49.1
53	DUF1642_domain-containing_protein	34407..34946	540	0.712	36.5	47.8	32.2	29.4	48.4
54	DUF1642_domain-containing_protein	34407..34946	540	0.712	36.5	47.8	32.2	29.4	48.4
55	DUF1642_domain-containing_protein	34998..35558	561	0.713	38.3	54	31	29.9	49.9
56	DUF1642_domain-containing_protein	30674..31237	564	0.746	36.5	53.7	30.3	25.5	47.3
57	DUF1642_domain-containing_protein	25216..25821	606	0.672	38.9	48	32.7	36.1	52.4
58	DUF1642_domain-containing_protein	24939..25544	606	0.672	38.9	48	32.7	36.1	52.4
59	DUF1642_domain-containing_protein	32719..33327	609	0.695	37.4	47.8	33.5	31	52.9
60	DUF1642_domain-containing_protein	2113..2721	609	0.695	37.4	47.8	33.5	31	52.9
61	DUF2481_family_protein	36512..36895	384	0.684	32.6	38.3	25.8	33.6	52
62	DUF2481_family_protein	6067..6450	384	0.684	32.6	38.3	25.8	33.6	52
63	DUF3800_domain-containing_protein	29458..30159	702	0.771	26.6	33.8	27.4	18.8	38.5
64	DUF3800_domain-containing_protein	17685..18386	702	0.771	26.6	33.8	27.4	18.8	38.5
65	DUF3800_domain-containing_protein	17494..18195	702	0.774	26.1	33.3	26.9	17.9	38.6
66	DUF3850_domain-containing_protein	33049..33276	228	0.745	35.5	50	27.6	28.9	55
67	DUF4065_domain-containing_protein	21942..22445	504	0.711	33.9	45.2	28.6	28	46.6
68	DUF722_domain-containing_protein	41185..41610	426	0.696	34	43	30.3	28.9	42.1
69	endolysin	16778..17341	564	0.692	38.8	42	44.7	29.8	56.9
70	endolysin	20257..21087	831	0.734	38.6	43.7	39	33.2	48.7
71	endolysin	20015..20884	870	0.717	38.5	43.8	37.9	33.8	52.3
72	endolysin	16422..17348	927	0.671	39.9	43.7	36.2	39.8	57.5
73	endolysin	16596..17540	945	0.699	38.9	43.2	37.8	35.9	53.2
74	endolysin	19966..20916	951	0.728	37.3	40.1	38.5	33.4	49.5
75	endolysin	19966..20916	951	0.728	37.3	40.1	38.5	33.4	49.5
76	endolysin	28363..29313	951	0.756	36	41.3	37.2	29.3	47.8
77	endolysin	23638..24603	966	0.709	39.8	45.3	37.6	36.3	52.4
78	Erf-like_ssDNA_annealing_protein	30400..31059	660	0.721	38.3	49.5	38.2	27.3	48.7
79	Erf-like_ssDNA_annealing_protein	39969..40667	699	0.741	37.5	46.8	38.6	27	48
80	excisionase_and_transcriptional_regulator	29220..29405	186	0.653	38.7	43.5	40.3	32.3	48.2
81	excisionase_and_transcriptional_regulator	30239..30424	186	0.701	40.3	43.5	41.9	35.5	44.3
82	excisionase_and_transcriptional_regulator	30239..30424	186	0.701	40.3	43.5	41.9	35.5	44.3
83	excisionase_and_transcriptional_regulator	38602..38787	186	0.71	39.8	43.5	41.9	33.9	41
84	ferredoxin	20061..20906	846	0.733	39	40.4	44.3	32.3	45
85	ferredoxin	19079..19930	852	0.72	39.8	41.2	44	34.2	48.6
86	gp10	6297..6680	384	0.729	31	41.4	30.5	21.1	45.9
87	Gp11_protein	7411..7773	363	0.783	31.4	43	28.9	22.3	37.9
88	gp12	9204..9578	375	0.674	39.7	46.4	36.8	36	57.8
89	gp13	7743..7892	150	0.711	32	46	24	26	31
90	gp16	11524..11880	357	0.694	38.7	52.9	30.3	32.8	47.2
91	gp17	11843..12055	213	0.653	39.4	52.1	36.6	29.6	43.9
92	Gp18_protein	15918..16322	405	0.731	35.8	48.9	31.1	27.4	48.6
93	gp20	18708..18983	276	0.775	32.2	48.9	25	22.8	46.5
94	gp20	17341..17706	366	0.802	32.5	50.8	27.9	18.9	40.3
95	gp21	18989..19153	165	0.646	37	52.7	25.5	32.7	43.9
96	gp21	18894..19211	318	0.694	35.5	45.3	30.2	31.1	48.4
97	Gp21-1_protein	18530..18676	147	0.613	34.7	38.8	32.7	32.7	57.1
98	gp22	19216..19374	159	0.675	35.2	56.6	22.6	26.4	35.6
99	gp22	18513..18851	339	0.714	38.3	52.2	33.6	29.2	42.3
100	Gp22_protein	18797..19030	234	0.682	30.3	48.7	16.7	25.6	35.2
101	gp23	19356..19721	366	0.647	35.5	45.1	24.6	36.9	49
102	gp23	19402..19767	366	0.653	34.4	44.3	23	36.1	53.6
103	gp23	19601..19996	396	0.666	37.9	50.8	29.5	33.3	44.6
104	Gp23_protein	19027..19218	192	0.761	22.9	29.7	17.2	21.9	38.3
105	Gp25_protein	complement(20701..21132)	432	0.761	28.5	37.5	24.3	23.6	44.2
106	gp26	21144..21320	177	0.708	31.1	35.6	30.5	27.1	44.3
107	gp26	21099..21533	435	0.726	37	37.9	43.4	29.7	53.6
108	gp26	21378..21929	552	0.69	29.3	35.9	28.8	23.4	43.8
109	gp26	21006..21734	729	0.733	38.1	44.9	41.6	28	46.9
110	gp27	21740..22081	342	0.648	37.4	48.2	26.3	37.7	56.4
111	gp27	22516..23148	633	0.755	34.8	41.7	41.2	21.3	40.1
112	gp28	22113..22238	126	0.755	34.1	47.6	26.2	28.6	34.9
113	gp29	22859..23017	159	0.778	32.7	35.8	37.7	24.5	37.1
114	gp29	23171..23353	183	0.696	24.6	29.5	23	21.3	32.3
115	Gp29_protein	22448..22690	243	0.791	36.2	44.4	38.3	25.9	43.5
116	gp30	23023..23175	153	0.644	30.1	27.5	31.4	31.4	46.8

117	gp30	23826..24032	207	0.648	30.4	37.7	24.6	29	39.7
118	gp30	22690..23136	447	0.65	36.2	45	28.9	34.9	43.9
119	Gp30_protein	22693..22878	186	0.696	34.9	46.8	29	29	45.8
120	gp31	23299..23463	165	0.677	29.1	21.8	36.4	29.1	48.3
121	Gp31_protein	23113..23265	153	0.74	33.3	41.2	33.3	25.5	40
122	gp32	23531..23764	234	0.682	29.9	44.9	17.9	26.9	49.7
123	gp32	complement(25549..26289)	741	0.757	33.2	42.9	32.4	24.3	36.6
124	gp32	complement(24940..25680)	741	0.762	33.1	42.9	32.4	23.9	36.4
125	Gp32_protein	23573..23824	252	0.654	31.7	44	22.6	28.6	45.2
126	gp33	23761..23952	192	0.749	22.4	29.7	18.8	18.8	33.3
127	gp33	complement(25702..26424)	723	0.689	33.1	43.6	29.9	25.7	47.4
128	Gp33_protein	23843..24133	291	0.636	35.4	37.1	32	37.1	49.4
129	gp35	complement(24915..25118)	204	0.761	27.9	38.2	22.1	23.5	38
130	gp35	27250..27477	228	0.701	35.1	42.1	30.3	32.9	44.2
131	gp35	complement(25436..26050)	615	0.795	34.1	48.8	34.6	19	40
132	gp36	27489..27773	285	0.758	28.8	36.8	23.2	26.3	42.9
133	gp36	complement(25132..25506)	375	0.732	30.9	36	28.8	28	45.1
134	Gp36_protein	25704..26072	369	0.636	40.1	48.8	33.3	38.2	55.5
135	gp37	complement(25646..25912)	267	0.688	26.2	24.7	29.2	24.7	61
136	gp37	27882..28148	267	0.707	37.1	47.2	32.6	31.5	58.9
137	gp37-1	complement(28077..28307)	231	0.715	36.4	57.1	24.7	27.3	45.3
138	gp38	28372..28548	177	0.666	40.1	47.5	33.9	39	37
139	Gp38_protein	26614..26907	294	0.654	33.7	35.7	26.5	38.8	48.7
140	gp39	28565..28720	156	0.731	31.4	48.1	25	21.2	50.2
141	gp39	27362..27604	243	0.738	37.9	45.7	38.3	29.6	45.9
142	gp39	complement(27560..27952)	393	0.75	35.4	46.6	41.2	18.3	41.3
143	Gp39_protein	26907..27077	171	0.717	34.5	56.1	24.6	22.8	30.8
144	gp39-1	complement(28722..28889)	168	0.785	33.3	44.6	32.1	23.2	33.7
145	gp40	29422..29511	90	0.719	33.3	33.3	33.3	33.3	39.6
146	gp40	27607..27792	186	0.66	34.9	48.4	24.2	32.3	51.4
147	gp40	28956..29192	237	0.733	35	51.9	32.9	20.3	39.5
148	Gp40_protein	27096..27530	435	0.721	33.6	45.5	24.8	30.3	41.8
149	gp41	29504..29632	129	0.69	39.5	53.5	30.2	34.9	49.7
150	gp41	28027..28179	153	0.721	31.4	41.2	31.4	21.6	51.6
151	gp41	29189..29470	282	0.68	29.8	37.2	22.3	29.8	45.1
152	Gp41_protein	27527..27802	276	0.779	30.8	43.5	25	23.9	39.6
153	gp41-1	complement(29472..29669)	198	0.687	34.3	40.9	33.3	28.8	34.2
154	gp42	29727..29921	195	0.705	29.7	43.1	21.5	24.6	39.3
155	Gp42_protein	27790..28176	387	0.675	36.2	50.4	28.7	29.5	43.1
156	Gp43_protein	28177..28599	423	0.697	34.3	43.3	34.8	24.8	40.7
157	Gp44	28596..28769	174	0.746	32.8	39.7	27.6	31	43
158	gp44	31175..31393	219	0.698	31.1	39.7	27.4	26	46.6
159	gp45	30124..30288	165	0.685	35.8	45.5	29.1	32.7	55
160	gp45	31390..31578	189	0.719	33.9	41.3	33.3	27	29.8
161	Gp45_protein	28766..29149	384	0.69	34.6	49.2	30.5	24.2	42.3
162	gp46	30438..30728	291	0.744	34.4	40.2	33	29.9	38.7
163	gp46	31458..31859	402	0.79	35.3	49.3	33.6	23.1	40.6
164	gp47	31856..32065	210	0.704	34.3	42.9	25.7	34.3	46.4
165	gp47	30906..31133	228	0.614	29.8	35.5	23.7	30.3	29.4
166	gp47	32223..32516	294	0.636	39.1	40.8	35.7	40.8	58.1
167	gp48	32513..32722	210	0.702	31.4	35.7	30	28.6	61
168	gp48	32066..32395	330	0.72	34.8	46.4	28.2	30	54.9
169	gp48	31151..31492	342	0.714	34.8	45.6	29.8	28.9	41.5
170	gp49	32392..32664	273	0.684	38.8	41.8	39.6	35.2	47
171	gp5	5146..5316	171	0.693	35.7	47.4	26.3	33.3	32.9
172	gp50	32664..32843	180	0.724	32.8	41.7	30	26.7	37.6
173	gp50	33324..33692	369	0.795	32	41.5	33.3	21.1	44.2
174	gp51	33689..33865	177	0.611	39	50.8	28.8	37.3	36.7
175	gp52	35555..35701	147	0.623	40.1	49	32.7	38.8	61
176	gp52	33379..33813	435	0.712	33.6	45.5	24.1	31	44.7
177	gp53	35717..35782	66	0.518	43.9	50	22.7	59.1	41
178	gp53	33810..34037	228	0.704	28.9	38.2	23.7	25	49.3
179	gp53	34310..34831	522	0.725	36.4	51.7	28.7	28.7	48.9
180	gp54	34076..34249	174	0.728	34.5	39.7	27.6	36.2	46.7
181	gp54	36570..36764	195	0.588	35.4	40	29.2	36.9	50.7
182	gp54	35779..36180	402	0.79	35.3	49.3	33.6	23.1	40.6
183	gp55	35265..35465	201	0.675	35.8	41.8	37.3	28.4	52.6
184	gp55	36751..36957	207	0.731	34.8	43.5	34.8	26.1	34.1
185	gp55	36177..36386	210	0.704	34.8	42.9	27.1	34.3	46.4
186	gp55	34246..34629	384	0.707	34.9	48.4	30.5	25.8	45.2
187	Gp55_protein	37300..37572	273	0.708	30	40.7	20.9	28.6	45.6
188	gp56	36958..37167	210	0.719	36.2	52.9	30	25.7	46.7
189	gp56	36383..36781	399	0.659	35.3	39.1	34.6	32.3	53
190	gp57	36787..36897	111	0.712	36	37.8	40.5	29.7	39.9
191	gp57	35968..36159	192	0.723	38.5	43.8	31.2	40.6	28.4
192	gp58	37606..38226	621	0.697	39.1	58.5	28	30.9	52.2
193	gp59	38267..38647	381	0.7	36.2	47.2	33.1	28.3	45.7
194	gp59	37225..37626	402	0.696	34.3	44	28.4	30.6	51.4
195	gp6	5074..5244	171	0.657	32.2	40.4	24.6	31.6	34.4
196	Gp6_protein	5277..5570	294	0.793	33	46.9	26.5	25.5	43.4
197	gp60	36888..37013	126	0.772	33.3	40.5	31	28.6	39.2
198	gp60	38607..38783	177	0.671	36.2	50.8	27.1	30.5	38.3
199	gp61	37025..37189	165	0.707	24.8	34.5	21.8	18.2	35.4
200	gp61	38137..38442	306	0.652	35.6	38.2	38.2	30.4	49.6
201	gp63	40588..40812	225	0.73	36.4	44	36	29.3	53.2
202	gp63	38006..38566	561	0.74	32.3	43.3	29.9	23.5	44.5
203	gp64	38942..39067	126	0.632	36.5	35.7	31	42.9	61
204	gp64	38663..38848	186	0.809	33.9	43.5	35.5	22.6	46.5
205	gp64	42047..42250	204	0.655	40.7	50	33.8	38.2	47.8
206	gp65	39078..39242	165	0.73	29.1	40	21.8	25.5	43.1
207	gp66	42446..42739	294	0.783	29.6	40.8	25.5	22.4	38.4
208	gp67	39741..39824	84	0.716	28.6	28.6	25	32.1	27.7
209	gp67	42757..42897	141	0.765	34.8	44.7	36.2	23.4	25.7
210	gp68	42884..43036	153	0.691	32.7	39.2	25.5	33.3	36.3
211	gp68	40061..40771	711	0.694	28.7	35	23.2	27.8	44.6
212	gp7	5254..5553	300	0.767	33	47	28	24	47
213	gp70	43296..43856	561	0.701	35.5	39.6	35.8	31	39.8
214	gp73	45087..45257	171	0.586	33.3	24.6	31.6	43.9	58
215	gp74	45270..45386	117	0.748	31.6	33.3	30.8	30.8	37.8
216	gp75	45576..45710	135	0.559	35.6	28.9	37.8	40	48.4
217	gp76	45667..45960	294	0.747	31	34.7	28.6	29.6	45.9
218	gp78	47342..47539	198	0.774	29.8	39.4	31.8	18.2	37.4
219	gp79	47670..47900	231	0.722	34.2	49.4	27.3	26	49.5
220	gp8	5537..5902	366	0.721	35	49.2	31.1	24.6	51.7
221	Gp8_protein	5888..6298	411	0.73	33.6	41.6	32.8	26.3	48.1
222	gp80	47928..48152	225	0.71	35.6	48	28	30.7	53.9
223	gp9	7872..8258	387	0.75	34.1	51.9	27.9	22.5	47.8
224	gp9	5899..6300	402	0.698	35.8	47	38.8	21.6	46.6
225	head_maturation_protease	3233..3979	747	0.787	34.5	46.2	35.7	21.7	42.5
226	head_maturation_protease	3140..3889	750	0.752	37.3	49.2	34	28.8	52.7
227	head_maturation_protease	3220..3972	753	0.796	34.1	47	34.7	20.7	40.9
228	head_maturation_protease	3132..3929	798	0.785	36	50.8	34.2	22.9	42.2
229	head_maturation_protease	5365..6474	1110	0.755	37.2	53.2	35.9	22.4	41.9
230	head_scaffolding_protein	4588..5157	570	0.665	41.8	55.8	34.2	35.3	42.6
231	head_scaffolding_protein	4588..5157	570	0.665	41.8	55.8	34.2	35.3	42.6
232	head_scaffolding_protein	4590..5159	570	0.666	40.2	54.7	30.5	35.3	44.8
233	head_scaffolding_protein	13023..13613	591	0.693	40.1	52.3	35	33	48.8
234	head_scaffolding_protein	4601..5200	600	0.686	40	52.5	35.5	32	46.2
235	head-tail_adaptor	7399..7731	333	0.697	29.7	40.5	18	30.6	56.9

236	head-tail_adaptor	5557..5895	339	0.708	32.2	41.6	29.2	25.7	43.8
237	head-tail_adaptor	5544..5888	345	0.763	32.8	41.7	31.3	25.2	43.2
238	head-tail_adaptor	15028..15390	363	0.714	36.6	47.9	31.4	30.6	45.1
239	head-tail_adaptor	6635..6997	363	0.717	35	45.5	30.6	28.9	44.8
240	head-tail_adaptor	6635..6997	363	0.717	35	45.5	30.6	28.9	44.8
241	head-tail_adaptor	6614..6976	363	0.722	36.6	47.9	31.4	30.6	47.9
242	head-tail_adaptor	6637..6999	363	0.752	34.7	44.6	32.2	27.3	42.5
243	head-tail_adaptor	7324..7692	369	0.675	28.7	39.8	17.9	28.5	48.1
244	head-tail_adaptor	14633..15028	396	0.682	38.9	45.5	39.4	31.8	61
245	head-tail_adaptor	6242..6637	396	0.689	37.9	45.5	38.6	29.5	54.9
246	head-tail_adaptor	6240..6635	396	0.7	37.9	45.5	39.4	28.8	52.7
247	head-tail_adaptor	6240..6635	396	0.7	37.9	45.5	39.4	28.8	52.7
248	head-tail_adaptor	6219..6614	396	0.703	38.9	47	40.2	29.5	56.3
249	head-tail_connector_protein	5270..5569	300	0.739	35	49	31	25	55.1
250	HeH/LEM_domain-containing_protein	6059..6217	159	0.692	44.7	52.8	41.5	39.6	57.3
251	HeH/LEM_domain-containing_protein	6082..6240	159	0.694	44	50.9	41.5	39.6	51.5
252	HeH/LEM_domain-containing_protein	6080..6238	159	0.7	44	50.9	43.4	37.7	46.7
253	HeH/LEM_domain-containing_protein	6080..6238	159	0.7	44	50.9	43.4	37.7	46.7
254	helix-turn-helix_transcriptional_regulator	complement(24666..24887)	222	0.74	34.2	43.2	33.8	25.7	54.6
255	helix-turn-helix_transcriptional_regulator	27015..27236	222	0.753	30.6	36.5	35.1	20.3	38
256	HNH_endonuclease	37642..37953	312	0.64	34.6	40.4	27.9	35.6	37.7
257	HNH_endonuclease	36983..37294	312	0.64	34.6	40.4	27.9	35.6	37.7
258	HNH_endonuclease	43416..43730	315	0.626	37.8	49.5	28.6	35.2	48.5
259	HNH_endonuclease	42295..42609	315	0.642	37.1	49.5	29.5	32.4	50.5
260	holin	20009..20257	249	0.706	37.3	44.6	27.7	39.8	51.5
261	holin	23381..23638	258	0.739	33.7	43	30.2	27.9	54
262	holin	16343..16603	261	0.714	36	50.6	27.6	29.9	45.4
263	holin	18813..19079	267	0.654	37.8	48.3	28.1	37.1	40.6
264	holin	28082..28363	282	0.683	37.9	38.3	40.4	35.1	58.1
265	holin	19682..19966	285	0.678	38.9	37.9	40	38.9	54.7
266	holin	19682..19966	285	0.678	38.9	37.9	40	38.9	54.7
267	holin	15905..16189	285	0.785	38.6	52.6	42.1	21.1	41.5
268	holin	19771..20061	291	0.679	40.9	49.5	40.2	33	46.1
269	holin	19725..20015	291	0.707	40.2	48.5	41.2	30.9	46.8
270	Holliday_junction_resolvase	5803..6063	261	0.66	35.6	48.3	24.1	34.5	43.8
271	Holliday_junction_resolvase	38549..38824	276	0.675	34.4	47.8	22.8	32.6	50.3
272	Holliday_junction_resolvase	38549..38824	276	0.675	34.4	47.8	22.8	32.6	50.3
273	Holliday_junction_resolvase	40225..40539	315	0.692	39	51.4	36.2	29.5	38.5
274	Holliday_junction_resolvase	34746..35066	321	0.667	39.6	47.7	37.4	33.6	47.8
275	Holliday_junction_resolvase	41063..41383	321	0.676	40.2	47.7	38.3	34.6	46.4
276	Holliday_junction_resolvase	35405..35725	321	0.691	38.6	51.4	36.4	28	40.3
277	Holliday_junction_resolvase	36104..36508	405	0.635	35.6	43.7	27.4	35.6	55.6
278	host_nuclease_inhibitor	33570..33737	168	0.631	39.9	48.2	33.9	37.5	61
279	host_nuclease_inhibitor	32050..32217	168	0.631	39.9	48.2	33.9	37.5	61
280	host_nuclease_inhibitor	33570..33737	168	0.631	39.9	48.2	33.9	37.5	61
281	HTH_DNA_binding_protein	29826..30119	294	0.717	32.3	41.8	30.6	24.5	44
282	hypothetical_protein	34964..35062	99	0.536	45.5	57.6	24.2	54.5	50.6
283	hypothetical_protein	34964..35062	99	0.536	45.5	57.6	24.2	54.5	50.6
284	hypothetical_protein	32474..32584	111	0.652	34.2	40.5	32.4	29.7	61
285	hypothetical_protein	7272..7385	114	0.628	39.5	60.5	26.3	31.6	40.2
286	hypothetical_protein	complement(35105..35224)	120	0.671	38.3	42.5	42.5	30	29.3
287	hypothetical_protein	35722..35841	120	0.79	26.7	40	27.5	12.5	31.4
288	hypothetical_protein	39204..39329	126	0.737	31.7	35.7	28.6	31	46.5
289	hypothetical_protein	39204..39329	126	0.737	31.7	35.7	28.6	31	46.5
290	hypothetical_protein	6443..6568	126	0.772	33.3	40.5	31	28.6	39.2
291	hypothetical_protein	39073..39201	129	0.675	38.8	51.2	27.9	37.2	43.7
292	hypothetical_protein	40307..40435	129	0.68	30.2	46.5	23.3	20.9	44.1
293	hypothetical_protein	40307..40435	129	0.68	30.2	46.5	23.3	20.9	44.1
294	hypothetical_protein	23252..23380	129	0.749	24.8	30.2	23.3	20.9	45.5
295	hypothetical_protein	7841..7978	138	0.791	33.3	45.7	28.3	26.1	33.5
296	hypothetical_protein	complement(22597..22737)	141	0.644	28.4	23.4	31.9	29.8	45.7
297	hypothetical_protein	7782..7931	150	0.726	34	50	24	28	32.4
298	hypothetical_protein	27525..27677	153	0.715	31.4	35.3	29.4	29.4	45.4
299	hypothetical_protein	23390..23542	153	0.74	33.3	41.2	33.3	25.5	40
300	hypothetical_protein	39353..39508	156	0.693	36.5	42.3	38.5	28.8	41.7
301	hypothetical_protein	39353..39508	156	0.693	36.5	42.3	38.5	28.8	41.7
302	hypothetical_protein	37355..37516	162	0.608	43.2	38.9	44.4	46.3	61
303	hypothetical_protein	28350..28511	162	0.723	34.6	42.6	25.9	35.2	61
304	hypothetical_protein	36710..36871	162	0.723	34	40.7	25.9	35.2	61
305	hypothetical_protein	28350..28511	162	0.723	34.6	42.6	25.9	35.2	61
306	hypothetical_protein	6580..6744	165	0.707	24.8	34.5	21.8	18.2	35.4
307	hypothetical_protein	35825..35992	168	0.779	26.8	35.7	25	19.6	43
308	hypothetical_protein	1125..1295	171	0.702	37.4	43.9	31.6	36.8	39.1
309	hypothetical_protein	5114..5284	171	0.778	29.2	45.6	22.8	19.3	35.8
310	hypothetical_protein	3325..3498	174	0.636	43.1	50	34.5	44.8	41.2
311	hypothetical_protein	34914..35087	174	0.733	32.8	32.8	27.6	37.9	30.2
312	hypothetical_protein	29256..29429	174	0.736	33.9	39.7	27.6	34.5	48.3
313	hypothetical_protein	25818..25994	177	0.604	39	50.8	27.1	39	47.7
314	hypothetical_protein	2718..2894	177	0.611	39	50.8	28.8	37.3	36.7
315	hypothetical_protein	28385..28561	177	0.698	35.6	57.6	22	27.1	42.6
316	hypothetical_protein	21166..21345	180	0.818	26.1	28.3	33.3	16.7	34.1
317	hypothetical_protein	21166..21345	180	0.818	26.1	28.3	33.3	16.7	34.1
318	hypothetical_protein	30208..30387	180	0.833	31.7	46.7	30	18.3	28.9
319	hypothetical_protein	26760..26942	183	0.684	39.9	50.8	32.8	36.1	46.1
320	hypothetical_protein	27105..27290	186	0.607	35.5	43.5	29	33.9	50.9
321	hypothetical_protein	36749..36934	186	0.673	37.1	37.1	37.1	37.1	46.7
322	hypothetical_protein	3988..4173	186	0.673	37.1	37.1	37.1	37.1	46.7
323	hypothetical_protein	36749..36934	186	0.673	37.1	37.1	37.1	37.1	46.7
324	hypothetical_protein	22970..23155	186	0.696	34.9	46.8	29	29	45.8
325	hypothetical_protein	31690..31875	186	0.754	33.3	46.8	27.4	25.8	38.3
326	hypothetical_protein	8218..8403	186	0.809	33.9	43.5	35.5	22.6	46.5
327	hypothetical_protein	30440..30628	189	0.645	38.6	38.1	36.5	41.3	36.7
328	hypothetical_protein	30440..30628	189	0.645	38.6	38.1	36.5	41.3	36.7
329	hypothetical_protein	38803..38991	189	0.647	38.1	38.1	34.9	41.3	30.3
330	hypothetical_protein	40539..40730	192	0.673	37	46.9	29.7	34.4	50.5
331	hypothetical_protein	40539..40730	192	0.673	37	46.9	29.7	34.4	50.5
332	hypothetical_protein	38284..38475	192	0.723	38.5	43.8	31.2	40.6	28.4
333	hypothetical_protein	5523..5714	192	0.723	38.5	43.8	31.2	40.6	28.4
334	hypothetical_protein	38284..38475	192	0.723	38.5	43.8	31.2	40.6	28.4
335	hypothetical_protein	23261..23452	192	0.749	25	32.8	18.8	23.4	35.9
336	hypothetical_protein	19304..19495	192	0.751	24	31.2	17.2	23.4	39.8
337	hypothetical_protein	4174..4368	195	0.569	36.4	36.9	32.3	40	61
338	hypothetical_protein	33593..33787	195	0.678	34.4	44.6	27.7	30.8	45
339	hypothetical_protein	39296..39490	195	0.724	30.8	47.7	20	24.6	45.4
340	hypothetical_protein	27521..27715	195	0.75	30.8	29.2	36.9	26.2	34.2
341	hypothetical_protein	35958..36152	195	0.75	30.8	29.2	36.9	26.2	34.2
342	hypothetical_protein	27521..27715	195	0.75	30.8	29.2	36.9	26.2	34.2
343	hypothetical_protein	35466..35663	198	0.714	35.9	47	30.3	30.3	50.8
344	hypothetical_protein	35466..35663	198	0.714	35.9	47	30.3	30.3	50.8
345	hypothetical_protein	36256..36462	207	0.623	43	44.9	39.1	44.9	47.1
346	hypothetical_protein	3495..3701	207	0.623	43	44.9	39.1	44.9	47.1
347	hypothetical_protein	36256..36462	207	0.623	43	44.9	39.1	44.9	47.1
348	hypothetical_protein	22365..22571	207	0.664	31.4	37.7	27.5	29	38.1
349	hypothetical_protein	22365..22571	207	0.664	31.4	37.7	27.5	29	38.1
350	hypothetical_protein	30802..31008	207	0.67	30.4	39.1	24.6	27.5	45.1
351	hypothetical_protein	1907..2116	210	0.702	31.4	35.7	30	28.6	61
352	hypothetical_protein	complement(22381..22590)	210	0.705	34.3	40	35.7	27.1	48.6
353	hypothetical_protein	complement(18421..18630)	210	0.733	32.4	40	34.3	22.9	44.7

354	hypothetical_protein	29996..30211	216	0.693	36.6	45.8	31.9	31.9	60.5
355	hypothetical_protein	32581..32799	219	0.703	38.8	43.8	32.9	39.7	49.9
356	hypothetical_protein	27646..27870	225	0.695	38.7	38.7	41.3	36	58
357	hypothetical_protein	23031..23264	234	0.649	30.3	42.3	17.9	30.8	40.9
358	hypothetical_protein	22135..22368	234	0.671	29.9	46.2	17.9	25.6	44.4
359	hypothetical_protein	22135..22368	234	0.671	29.9	46.2	17.9	25.6	44.4
360	hypothetical_protein	16186..16419	234	0.674	33.8	48.7	23.1	29.5	61
361	hypothetical_protein	30572..30805	234	0.679	31.2	48.7	16.7	28.2	34
362	hypothetical_protein	19074..19307	234	0.7	29.1	46.2	17.9	23.1	42.3
363	hypothetical_protein	36164..36400	237	0.733	35	51.9	32.9	20.3	39.5
364	hypothetical_protein	complement(28504..28746)	243	0.696	30.9	37	22.2	33.3	55.7
365	hypothetical_protein	complement(36864..37106)	243	0.696	30.9	37	22.2	33.3	55.7
366	hypothetical_protein	complement(28504..28746)	243	0.696	30.9	37	22.2	33.3	55.7
367	hypothetical_protein	26860..27102	243	0.738	37.9	45.7	38.3	29.6	51.5
368	hypothetical_protein	22725..22967	243	0.791	36.2	44.4	38.3	25.9	43.5
369	hypothetical_protein	27867..28112	246	0.639	38.6	53.7	28	34.1	55.1
370	hypothetical_protein	30384..30629	246	0.691	35.4	45.1	31.7	29.3	51.8
371	hypothetical_protein	23850..24101	252	0.654	31.7	44	22.6	28.6	45.2
372	hypothetical_protein	27814..28065	252	0.681	31.3	41.7	26.2	26.2	45.6
373	hypothetical_protein	32799..33056	258	0.751	37.6	55.8	31.4	25.6	33.8
374	hypothetical_protein	28993..29259	267	0.663	31.1	44.9	21.3	27	53
375	hypothetical_protein	27031..27297	267	0.707	37.1	47.2	32.6	31.5	58.9
376	hypothetical_protein	35467..35733	267	0.707	37.1	47.2	32.6	31.5	58.9
377	hypothetical_protein	27031..27297	267	0.707	37.1	47.2	32.6	31.5	58.9
378	hypothetical_protein	33752..34021	270	0.693	35.6	36.7	34.4	35.6	39
379	hypothetical_protein	33752..34021	270	0.693	35.6	36.7	34.4	35.6	39
380	hypothetical_protein	37959..38231	273	0.743	28.6	40.7	22	23.1	55
381	hypothetical_protein	32202..32477	276	0.696	38.4	42.4	39.1	33.7	46.2
382	hypothetical_protein	24129..24410	282	0.634	35.8	38.3	33	36.2	49.7
383	hypothetical_protein	36397..36678	282	0.66	29.4	37.2	18.1	33	42.8
384	hypothetical_protein	28037..28318	282	0.666	29.4	38.3	18.1	31.9	42.2
385	hypothetical_protein	28037..28318	282	0.666	29.4	38.3	18.1	31.9	42.2
386	hypothetical_protein	1626..1910	285	0.654	37.9	41.1	36.8	35.8	41.6
387	hypothetical_protein	27727..28011	285	0.728	30.9	34.7	30.5	27.4	32.3
388	hypothetical_protein	27727..28011	285	0.728	30.9	34.7	30.5	27.4	32.3
389	hypothetical_protein	30927..31211	285	0.793	35.8	44.2	34.7	28.4	37.6
390	hypothetical_protein	1310..1597	288	0.653	34.7	44.8	24	35.4	50.9
391	hypothetical_protein	36459..36749	291	0.716	34.4	49.5	22.7	30.9	37.3
392	hypothetical_protein	3698..3988	291	0.716	34.4	49.5	22.7	30.9	37.3
393	hypothetical_protein	36459..36749	291	0.716	34.4	49.5	22.7	30.9	37.3
394	hypothetical_protein	5294..5593	300	0.751	32.7	45	27	26	42.9
395	hypothetical_protein	19380..19685	306	0.662	36.3	44.1	32.4	32.4	45.2
396	hypothetical_protein	19380..19685	306	0.662	36.3	44.1	32.4	32.4	45.2
397	hypothetical_protein	27777..28082	306	0.667	36.6	44.1	32.4	33.3	44.5
398	hypothetical_protein	33288..33596	309	0.755	35.6	47.6	34	25.2	40.8
399	hypothetical_protein	43090..43416	327	0.738	33.9	43.1	35.8	22.9	45.2
400	hypothetical_protein	31876..32205	330	0.717	34.5	45.5	28.2	30	44.8
401	hypothetical_protein	7434..7796	363	0.791	31.7	45.5	26.4	23.1	41
402	hypothetical_protein	15527..15892	366	0.65	35.8	45.9	24.6	36.9	50.3
403	hypothetical_protein	5577..5942	366	0.753	34.4	50	29.5	23.8	48.8
404	hypothetical_protein	25981..26349	369	0.636	40.1	48.8	33.3	38.2	55.5
405	hypothetical_protein	29426..29809	384	0.707	34.6	47.7	30.5	25.8	45.1
406	hypothetical_protein	35084..35467	384	0.717	32.6	48.4	26.6	22.7	43.5
407	hypothetical_protein	6337..6720	384	0.758	31.5	42.2	30.5	21.9	39.6
408	hypothetical_protein	30626..31015	390	0.607	39	32.3	43.8	40.8	47.6
409	hypothetical_protein	34018..34410	393	0.74	37.4	48.9	32.1	31.3	42.6
410	hypothetical_protein	34018..34410	393	0.74	37.4	48.9	32.1	31.3	42.6
411	hypothetical_protein	35065..35463	399	0.732	36.6	49.6	29.3	30.8	39.1
412	hypothetical_protein	35065..35463	399	0.732	36.6	49.6	29.3	30.8	39.1
413	hypothetical_protein	29062..29460	399	0.738	37.6	49.6	30.8	32.3	45.5
414	hypothetical_protein	5939..6340	402	0.657	37.3	47.8	38.8	25.4	46.1
415	hypothetical_protein	37383..37784	402	0.723	32.3	45.5	26.1	25.4	44.3
416	hypothetical_protein	4622..5023	402	0.723	32.3	45.5	26.1	25.4	44.3
417	hypothetical_protein	37383..37784	402	0.723	32.3	45.5	26.1	25.4	44.3
418	hypothetical_protein	18376..18792	417	0.714	35.5	48.2	31.7	26.6	45.5
419	hypothetical_protein	5875..6291	417	0.793	30.9	42.4	30.2	20.1	42.2
420	hypothetical_protein	complement(20978..21409)	432	0.76	29.2	38.9	24.3	24.3	45.5
421	hypothetical_protein	28562..28996	435	0.719	34.3	46.2	24.8	31.7	46.7
422	hypothetical_protein	7561..8121	561	0.74	32.3	43.3	29.9	23.5	44.5
423	hypothetical_protein	complement(20174..20746)	573	0.765	30.5	42.4	25.7	23.6	56.4
424	hypothetical_protein	complement(24934..25548)	615	0.783	34.5	48.8	34.6	20	41
425	hypothetical_protein	complement(24852..25574)	723	0.689	33.1	43.6	29.9	25.7	47.4
426	hypothetical_protein	complement(24852..25574)	723	0.689	33.1	43.6	29.9	25.7	47.4
427	hypothetical_protein	complement(24090..24830)	741	0.749	33.6	42.9	32.4	25.5	37.6
428	hypothetical_protein	complement(24090..24830)	741	0.749	33.6	42.9	32.4	25.5	37.6
429	Ig-like_domain-containing_protein	8184..8423	240	0.751	41.2	53.8	46.2	23.8	50.3
430	Ig-like_domain-containing_protein	8111..8443	333	0.706	42.6	53.2	43.2	31.5	58.5
431	Ig-like_domain-containing_protein	complement(32525..33265)	741	0.749	33.6	42.9	32.4	25.5	37.6
432	integrase	complement(24147..25301)	1155	0.693	33.4	38.7	31.2	30.4	50.9
433	integrase	complement(19690..20844)	1155	0.726	31.9	38.2	30.9	26.8	45.9
434	integrase	complement(23646..24800)	1155	0.726	32.4	39	31.4	26.8	41
435	integrase	complement(19413..20567)	1155	0.726	31.9	38.2	30.9	26.8	45.9
436	integrase	complement(26288..27463)	1176	0.716	33.7	42.1	31.6	27.3	46.3
437	integrase	complement(22820..24022)	1203	0.687	37.6	46.6	37.4	28.7	49.5
438	integrase	complement(22820..24022)	1203	0.687	37.6	46.6	37.4	28.7	49.5
439	integrase	complement(31257..32459)	1203	0.691	37.7	47.1	37.7	28.4	49.5
440	integrase	complement(24281..25483)	1203	0.717	36.6	46.1	37.7	25.9	46
441	integrase	complement(23518..24876)	1359	0.754	32.1	43.3	29.1	23.8	45.1
442	kinase	41570..42100	531	0.737	35	46.9	29.4	28.8	59.3
443	kinase	36103..36636	534	0.685	34.3	42.7	25.3	34.8	47.6
444	kinase	40542..41180	639	0.685	34.9	42.3	29.1	33.3	61
445	kinase	35336..35977	642	0.685	33.6	41.1	25.7	34.1	48.2
446	LysM_peptidoglycan-binding_domain-containing_protein	23155..23370	216	0.874	28.7	44.4	29.2	12.5	32.6
447	major_capsid_protein	5183..6082	900	0.76	38.8	47.3	39	30	46.8
448	major_capsid_protein	5181..6080	900	0.768	38.2	49	38	27.7	42.6
449	major_capsid_protein	5181..6080	900	0.768	38.2	49	38	27.7	42.6
450	major_head_protein	5223..6059	837	0.787	38.6	55.6	37.3	22.9	44.9
451	major_head_protein	6944..7849	906	0.767	40.6	55	41.7	25.2	42.6
452	major_head_protein	13613..14614	1002	0.759	41.9	53	43.1	29.6	41.2
453	major_head_protein	3916..5067	1152	0.746	39.1	52.9	34.9	29.4	48.6
454	major_head_protein	3956..5107	1152	0.764	36.5	51.3	33.9	24.2	41.7
455	major_head_protein	3976..5148	1173	0.748	40.2	51.9	40.7	27.9	43.4
456	major_head_protein	3969..5141	1173	0.762	39.1	50.6	39.4	27.4	43.7
457	major_head_protein	join(3976..5126,5128..5290)	1314	0.735	39.3	50.9	38.8	28.3	44.1
458	major_tail_protein	7747..8181	435	0.747	43.7	52.4	45.5	33.1	38.9
459	major_tail_protein	7724..8161	438	0.702	45.4	55.5	43.2	37.7	42.4
460	major_tail_protein	16138..16575	438	0.722	45	54.8	43.8	36.3	41.6
461	major_tail_protein	7745..8182	438	0.752	43.8	54.8	44.5	32.2	54.3
462	major_tail_protein	7745..8182	438	0.752	43.8	54.8	44.5	32.2	54.3
463	major_tail_protein_a	6712..7290	579	0.804	36.1	49.7	38.9	19.7	38.3
464	major_tail_protein_b	join(6712..7286,7288..7363)	651	0.786	36.7	51.2	38.2	20.7	41.9
465	MazF-like_growth_inhibitor	22442..22834	393	0.72	29.5	36.6	28.2	23.7	44.8
466	metal-dependent_hydrolase	34168..34905	738	0.706	35.4	43.5	29.3	33.3	47.8
467	metallo-protease	complement(21183..21575)	393	0.765	30.3	42.7	26.7	21.4	44.1
468	metallo-protease	complement(28000..28419)	420	0.736	30	37.1	29.3	23.6	44.8
469	metallo-protease	complement(21460..21912)	453	0.751	30.5	41.7	27.2	22.5	41.8
470	metallo-protease	complement(26101..26553)	453	0.783	30	42.4	25.8	21.9	51.3
471	metallo-protease	complement(25599..26051)	453	0.783	30	42.4	25.8	21.9	51.3

472	metallo-protease	complement(25831..26322)	492	0.738	29.5	41.5	25.6	21.3	45.7
473	metallo-protease	complement(26681..27172)	492	0.738	29.5	41.5	25.6	21.3	45.7
474	metallo-protease	complement(34266..34757)	492	0.738	29.5	41.5	25.6	21.3	45.7
475	metallo-protease	complement(25831..26322)	492	0.738	29.5	41.5	25.6	21.3	45.7
476	minor_head_protein	15390..15728	339	0.644	38.6	47.8	31	37.2	44.5
477	minor_head_protein	6976..7314	339	0.655	41.9	51.3	34.5	39.8	51.1
478	minor_head_protein	6997..7335	339	0.671	40.4	51.3	33.6	36.3	53.9
479	minor_head_protein	6997..7335	339	0.671	40.4	51.3	33.6	36.3	53.9
480	minor_head_protein	6999..7337	339	0.676	41	51.3	33.6	38.1	61
481	minor_head_protein	15728..16135	408	0.709	34.3	40.4	30.1	32.4	46.3
482	minor_head_protein	7314..7721	408	0.729	33.3	39	29.4	31.6	43.6
483	minor_head_protein	7335..7742	408	0.738	32.1	39	30.1	27.2	41.7
484	minor_head_protein	7335..7742	408	0.738	32.1	39	30.1	27.2	41.7
485	minor_head_protein	7337..7744	408	0.745	33.1	39.7	30.1	29.4	46
486	minor_head_protein	3383..4522	1140	0.688	39.6	50	36.1	32.9	51.9
487	minor_head_protein	3370..4509	1140	0.703	38.9	50.3	35	31.3	52.9
488	minor_head_protein	3370..4509	1140	0.703	38.9	50.3	35	31.3	52.9
489	minor_head_protein	3372..4511	1140	0.709	39.4	50.5	36.1	31.6	49.2
490	minor_head_protein	11805..12944	1140	0.715	39.2	49.7	35.8	32.1	46.5
491	minor_head_protein_and_DNA_pilot	3740..5149	1410	0.724	36.2	45.3	38.1	25.3	49.1
492	minor_tail_protein	26184..27251	1068	0.705	39.1	49.2	37.9	30.3	53
493	minor_tail_protein	17767..18834	1068	0.739	37.1	48.3	36.8	26.1	49.4
494	minor_tail_protein	17787..18854	1068	0.744	36.7	49.2	35.7	25.3	51.2
495	minor_tail_protein	17787..18854	1068	0.744	36.7	49.2	35.7	25.3	51.2
496	minor_tail_protein	17809..18882	1074	0.746	36.5	45.3	39.1	25.1	49.2
497	minor_tail_protein	13863..14984	1122	0.731	38.1	51.3	36.1	26.7	48.4
498	minor_tail_protein	13885..15006	1122	0.749	38.7	52.1	36.9	27	45.3
499	Mu_Gam-like_end_protection	39487..39963	477	0.634	36.7	45.3	28.3	36.5	47.8
500	Mu_Gam-like_end_protection	29918..30394	477	0.657	35.4	45.9	28.3	32.1	48.1
501	Mu_Gam-like_end_protection	29151..29630	480	0.708	32.7	46.2	25	26.9	52.5
502	Mu_Gam-like_end_protection	35469..35948	480	0.727	32.1	46.2	25.6	24.4	50.1
503	Mu_Gam-like_end_protection	29811..30290	480	0.731	30.8	48.1	21.9	22.5	45.3
504	Mu_Gam-like_end_protection	34631..35110	480	0.747	30.2	48.1	21.9	20.6	44.1
505	neck_protein	6281..6703	423	0.747	30.3	41.1	27.7	22	40.1
506	neck_protein	6288..6710	423	0.794	29.1	39.7	27.7	19.9	36.9
507	ORF58	36897..36989	93	0.73	37.6	45.2	41.9	25.8	25.1
508	ParB_N-terminal_domain-containing_protein	45957..47294	1338	0.7	35.3	45.1	30.9	29.8	48.3
509	PcfJ_domain-containing_protein	39214..40575	1362	0.7	33.3	41.2	28.9	30	49
510	pentapeptide_repeat-containing_protein	34828..35268	441	0.733	35.4	46.3	32	27.9	39.3
511	pentapeptide_repeat-containing_protein	31208..31693	486	0.757	35.2	43.8	35.8	25.9	42.9
512	pentapeptide_repeat-containing_protein	32840..33379	540	0.79	33.9	48.3	33.9	19.4	35.5
513	peptidoglycan_DD-metalloendopeptidase_family_protein	12057..16772	4716	0.722	39.1	48.5	41.2	27.7	48.6
514	phenylalanyl-tRNA_synthetase_subunit_beta	36086..36259	174	0.636	43.1	50	34.5	44.8	41.2
515	phenylalanyl-tRNA_synthetase_subunit_beta	36086..36259	174	0.636	43.1	50	34.5	44.8	41.2
516	portal_protein	2005..3135	1131	0.712	34.1	42.4	28.4	31.6	49.2
517	portal_protein	2013..3143	1131	0.727	34	43.5	28.6	30	49.5
518	portal_protein	2055..3242	1188	0.721	33	40.4	30.6	28	45.7
519	portal_protein	2068..3255	1188	0.751	31.6	42.2	30.1	22.7	40.3
520	portal_protein	2362..3747	1386	0.697	37.4	47	35.1	30.1	49.3
521	portal_protein	10345..11799	1455	0.68	37.7	46.2	30.3	36.7	45.9
522	portal_protein	1876..3366	1491	0.657	38.9	47.1	29.8	39.8	52.5
523	portal_protein	1874..3364	1491	0.661	37.9	46.1	30	37.6	53.4
524	portal_protein	1874..3364	1491	0.661	37.9	46.1	30	37.6	53.4
525	portal_protein	1878..3377	1500	0.675	37.3	45.4	30	36.4	50
526	putative_DNA_replication_protein	108..362	255	0.738	33.7	48.2	23.5	29.4	40.1
527	putative_structural_protein	6707..7285	579	0.756	37	48.7	37.3	24.9	42.7
528	RecE-like_recombination_exonuclease	31811..32770	960	0.718	37.4	48.4	32.8	30.9	45.9
529	RecT-like_ssDNA_annealing_protein	32773..33588	816	0.713	37.6	47.1	34.6	31.2	47.3
530	replication_initiation_protein	26932..27207	276	0.699	33.7	43.5	30.4	27.2	41.3
531	replication_initiation_protein	34917..35834	918	0.708	34.1	44.8	30.1	27.5	44.4
532	replisome_organizer	31076..32053	978	0.678	37.3	49.7	29.1	33.1	49.8
533	RNA_ligase	33955..34842	888	0.686	38.9	52.4	32.1	32.1	47.6
534	RNA_polymerase_sigma_factor	39601..40173	573	0.707	37.3	49.2	31.4	31.4	45.6
535	RNA_polymerase_sigma_factor	39601..40173	573	0.707	37.3	49.2	31.4	31.4	45.6
536	RNA-binding_protein	37144..37371	228	0.685	37.7	50	27.6	35.5	49.3
537	RNA-binding_protein	4383..4610	228	0.685	37.7	50	27.6	35.5	49.3
538	RNA-binding_protein	37144..37371	228	0.685	37.7	50	27.6	35.5	49.3
539	RNA-binding_protein	43033..43284	252	0.672	35.3	50	25	31	61
540	RusA-like_Holliday_junction_resolvase	38545..38949	405	0.641	36.5	37	31.9	40.7	42.5
541	RusA-like_Holliday_junction_resolvase	37148..37585	438	0.66	37.7	44.5	35.6	32.9	47.5
542	Sak4-like_ssDNA_annealing_protein	30303..30992	690	0.719	33.5	44.3	30.4	25.7	44.5
543	Sak4-like_ssDNA_annealing_protein	35961..36650	690	0.765	33.2	44.8	30.9	23.9	45
544	Sak4-like_ssDNA_annealing_protein	35123..35815	693	0.675	36.1	43.7	32.9	31.6	52.2
545	Sak4-like_ssDNA_annealing_protein	29640..30332	693	0.761	33.2	44.2	30.7	24.7	45.7
546	SAP_domain_protein	5164..5283	120	0.615	34.2	45	22.5	35	37.2
547	SbcC-like_subunit_of_palindrome_specific_endonuclease	31479..33422	1944	0.717	33.4	47.8	25.5	27	49.1
548	single_strand_DNA_binding_protein	37623..38105	483	0.742	39.5	52.2	38.5	28	45.6
549	single_strand_DNA_binding_protein	37781..38263	483	0.758	40.2	54	39.1	27.3	46
550	single_strand_DNA_binding_protein	5020..5502	483	0.758	40.2	54	39.1	27.3	46
551	single_strand_DNA_binding_protein	37781..38263	483	0.758	40.2	54	39.1	27.3	46
552	single_strand_DNA_binding_protein	37158..37640	483	0.767	31.9	44.1	27.3	24.2	43.3
553	single_strand_DNA_binding_protein	35465..35947	483	0.773	39.1	52.8	39.8	24.8	42.7
554	single_strand_DNA_binding_protein	31677..32162	486	0.782	32.1	46.9	25.9	23.5	50
555	single_strand_DNA_binding_protein	32335..32820	486	0.784	31.3	44.4	25.9	23.5	39.7
556	single_strand_DNA_binding_protein	37995..38480	486	0.791	31.9	46.9	26.5	22.2	48.9
557	site-specific_integrase	40812..41756	945	0.689	36.6	46	32.1	31.7	51.1
558	site-specific_integrase	29040..29984	945	0.693	36.5	46.3	32.4	30.8	52.4
559	structural_protein_with_Ig_domain	8151..8444	294	0.699	44.6	54.1	46.9	32.7	41.1
560	structural_protein_with_Ig_domain	8151..8444	294	0.699	44.6	54.1	46.9	32.7	41.1
561	structural_protein_with_Ig_domain	16544..16837	294	0.736	41.2	53.1	45.9	24.5	43
562	tail_assembly_chaperone	8476..8898	423	0.743	31	45.4	21.3	26.2	37.9
563	tail_assembly_chaperone	16890..17312	423	0.754	31.2	45.4	21.3	27	39.2
564	tail_assembly_chaperone	8497..8919	423	0.757	31.2	44.7	21.3	27.7	42.3
565	tail_assembly_chaperone	8497..8919	423	0.757	31.2	44.7	21.3	27.7	42.3
566	tail_assembly_chaperone	8498..8920	423	0.771	31.4	46.8	22	25.5	40.5
567	tail_completion_or_Neck1_protein	8605..9204	600	0.633	39.3	46	37	35	55.6
568	tail_family_protein	11055..11762	708	0.763	30.2	43.6	25.8	21.2	39.5
569	tail_family_protein	11033..11740	708	0.763	30.2	43.6	25.8	21.2	39.5
570	tail_family_protein	14928..15746	819	0.744	35.8	42.9	38.5	26	46.4
571	tail_family_protein	14880..15707	828	0.709	36	37.7	34.1	36.2	48.4
572	tail_family_protein	14901..15728	828	0.714	35.6	38	34.4	34.4	50.8
573	tail_family_protein	14901..15728	828	0.714	35.6	38	34.4	34.4	50.8
574	tail_family_protein	23297..24124	828	0.719	35.4	37	34.4	34.8	46.6
575	tail_fiber_protein	14981..15313	333	0.717	36.9	53.2	28.8	28.8	44.5
576	tail_fiber_protein	18851..19192	342	0.709	38.6	56.1	30.7	28.9	43.5
577	tail_fiber_protein	18851..19192	342	0.709	38.6	56.1	30.7	28.9	43.5
578	tail_fiber_protein	15003..15344	342	0.721	36.8	52.6	28.1	29.8	41.9
579	tail_fiber_protein	27248..27589	342	0.725	36.8	52.6	28.9	28.9	41.5
580	tail_fiber_protein	18831..19172	342	0.732	37.4	56.1	30.7	25.4	43.5
581	tail_length_tape_measure_protein	7956..11036	3081	0.734	37.6	49.3	37	26.5	43.4
582	tail_length_tape_measure_protein	7978..11058	3081	0.745	36.9	48	37.2	25.4	43
583	tail_length_tape_measure_protein	9517..14883	5367	0.724	40.5	48.5	42.7	30.1	47.5
584	tail_length_tape_measure_protein	17934..23300	5367	0.726	39.9	47.7	42.3	29.6	48.7
585	tail_length_tape_measure_protein	9538..14904	5367	0.743	39.3	48	42.3	27.7	46.2
586	tail_length_tape_measure_protein	9538..14904	5367	0.743	39.3	48	42.3	27.7	46.2
587	tail_length_tape_measure_protein	9542..14926	5385	0.749	39.3	48.4	42.4	27.1	47.8
588	tail_protein	15262..15486	225	0.764	30.7	40	28	24	33.1
589	tail_protein	15496..15939	444	0.668	36.5	39.9	33.8	35.8	50
590	tail_protein	17954..18397	444	0.677	35.8	37.2	32.4	37.8	48

591	tail_protein	22268..22714	447	0.695	34.7	32.9	36.2	34.9	52.8
592	tail_protein	19179..19634	456	0.663	36.4	38.8	32.2	38.2	49.4
593	tail_protein	6702..7289	588	0.754	38.8	53.6	36.7	26	45.9
594	tail_protein	6741..7328	588	0.756	38.1	53.1	35.7	25.5	46
595	tail_protein	16745..17770	1026	0.696	39.9	46.2	40.1	33.3	54.1
596	tail_protein	25162..26187	1026	0.699	41	46.5	40.6	36	48.8
597	tail_protein	16765..17790	1026	0.706	39.6	46.5	40.1	32.2	51.5
598	tail_protein	16765..17790	1026	0.706	39.6	46.5	40.1	32.2	51.5
599	tail_protein	16781..17809	1029	0.756	35.6	42	37.9	26.8	49.5
600	tail_protein	11762..13888	2127	0.724	35.9	45	37.2	25.4	48.3
601	tail_protein	11740..13866	2127	0.726	35.8	45	37.1	25.4	48.1
602	tail_protein	14528..16822	2295	0.722	36.7	46.7	35	28.4	47.3
603	tail_protein_with_endopeptidase_domain	24139..25161	1023	0.693	36.4	46	29	34	52.4
604	tail_protein_with_endopeptidase_domain	15742..16764	1023	0.696	36.6	46.9	29	33.7	53.3
605	tail_protein_with_endopeptidase_domain	15742..16764	1023	0.696	36.6	46.9	29	33.7	53.3
606	tail_protein_with_endopeptidase_domain	15722..16744	1023	0.701	36.2	45.7	29.6	33.1	48.7
607	tail_protein_with_endopeptidase_domain	15755..16780	1026	0.733	33.4	43.6	31.3	25.4	49.6
608	tail_protein_with_endopeptidase_domain	13670..15262	1593	0.75	32.3	39.4	33.5	23.9	46
609	tail_sheath	10061..11056	996	0.714	41.4	53.9	39.2	31	51.3
610	tail_spike_protein	15486..17405	1920	0.775	31.6	39.2	35.6	20	43.1
611	tail_tape_measure_protein	7947..12866	4920	0.742	38.2	48.4	39.8	26.5	47.1
612	tail_tape_measure_protein	7908..12830	4923	0.744	37.8	47.7	39.4	26.4	47.4
613	tail_terminator	9568..10056	489	0.695	40.1	49.1	37.4	33.7	54.6
614	terminase_large_subunit	530..1861	1332	0.663	38.2	48	28.6	38.1	56.1
615	terminase_large_subunit	530..1861	1332	0.663	38.2	48	28.6	38.1	56.1
616	terminase_large_subunit	532..1863	1332	0.679	37	46.6	28.8	35.6	54.9
617	terminase_large_subunit	8956..10287	1332	0.687	37	47.5	29.1	34.5	52.5
618	terminase_large_subunit	534..1865	1332	0.698	37.2	48.6	29.1	34	51.2
619	terminase_large_subunit	888..2348	1461	0.657	40.6	50.1	36.3	35.3	52.5
620	terminase_large_subunit	358..2001	1644	0.716	37.1	50.5	32.3	28.5	48.1
621	terminase_large_subunit	350..1993	1644	0.726	36.5	50.2	32.1	27.2	48.3
622	terminase_large_subunit	388..2040	1653	0.752	34.5	43.9	32.5	27.2	45.1
623	terminase_large_subunit	402..2054	1653	0.753	34.8	44.6	33	26.7	46.2
624	terminase_small_subunit	62..361	300	0.67	31	39	26	28	46.4
625	terminase_small_subunit	54..353	300	0.691	30	38	26	26	50.4
626	terminase_small_subunit	59..418	360	0.774	31.4	46.7	27.5	20	43.6
627	terminase_small_subunit	42..404	363	0.785	30.6	45.5	27.3	19	40.3
628	terminase_small_subunit	19..549	531	0.694	40.1	53.7	35	31.6	54.2
629	terminase_small_subunit	19..549	531	0.694	40.1	53.7	35	31.6	54.2
630	terminase_small_subunit	21..563	543	0.666	39.8	52.5	33.7	33.1	48.5
631	terminase_small_subunit	23..565	543	0.695	40.3	53	37	30.9	51.6
632	terminase_small_subunit	8445..8987	543	0.695	40.3	53	37	30.9	51.6
633	terminase_small_subunit	26..910	885	0.641	41.5	47.8	37.6	39	53.8
634	transcriptional_activator	43937..44359	423	0.721	34.3	46.1	31.2	25.5	45.4
635	transcriptional_activator	39261..39695	435	0.703	35.4	49	26.2	31	48.2
636	transcriptional_activator	37208..37642	435	0.718	33.6	46.9	26.9	26.9	45.2
637	transcriptional_activator	6763..7197	435	0.718	33.6	46.9	26.9	26.9	45.2
638	transcriptional_regulator	complement(26355..26660)	306	0.716	31	40.2	28.4	24.5	55.6
639	transcriptional_regulator	complement(27205..27510)	306	0.716	31	40.2	28.4	24.5	55.6
640	transcriptional_regulator	complement(34790..35095)	306	0.716	31	40.2	28.4	24.5	55.6
641	transcriptional_regulator	complement(26355..26660)	306	0.716	31	40.2	28.4	24.5	55.6
642	transcriptional_regulator	complement(21929..22249)	321	0.711	36.8	48.6	31.8	29.9	43.8
643	transcriptional_regulator	complement(26570..26890)	321	0.737	35.8	47.7	31.8	28	52.1
644	transcriptional_regulator	complement(26068..26388)	321	0.737	35.8	47.7	31.8	28	52.1
645	transcriptional_regulator	complement(21652..21972)	321	0.762	33.6	47.7	30.8	22.4	39.8
646	transcriptional_regulator	42101..42526	426	0.7	34.5	40.1	32.4	31	45.7
647	transcriptional_regulator	36637..37062	426	0.713	34.7	44.4	33.8	26.1	53
648	transcriptional_regulator	35978..36403	426	0.713	34.7	44.4	33.8	26.1	53
649	transcriptional_regulator	complement(28436..28864)	429	0.704	34.5	44.1	32.2	27.3	48.9
650	transcriptional_regulator	complement(26424..26852)	429	0.804	30.1	47.6	27.3	15.4	37.8
651	transcriptional_regulator	29708..30232	525	0.695	37.9	46.3	36.6	30.9	50.2
652	transcriptional_regulator	29708..30232	525	0.695	37.9	46.3	36.6	30.9	50.2
653	transcriptional_regulator	38071..38595	525	0.697	36.8	46.9	33.7	29.7	49.9
654	transcriptional_regulator	28689..29213	525	0.698	38.1	46.3	38.3	29.7	49.8
655	transcriptional_regulator	29287..29811	525	0.808	33	45.1	32	21.7	34.9
656	transcriptional_regulator	30645..31178	534	0.715	38.8	47.8	37.1	31.5	44.1
657	transcriptional_regulator,_D5-like	41769..42449	681	0.684	34.2	39.2	33	30.4	48.8
658	transcriptional_regulator,_D5-like	29997..30677	681	0.702	32.9	37.9	31.7	29.1	44.2
659	transcriptional_repressor	29025..29210	186	0.636	38.2	40.3	37.1	37.1	52
660	transcriptional_repressor	27157..27360	204	0.71	34.8	39.7	29.4	35.3	37.8
661	transcriptional_repressor	26655..26858	204	0.71	34.8	39.7	29.4	35.3	37.8
662	transcriptional_repressor	22516..22722	207	0.744	33.8	42	27.5	31.9	45
663	transcriptional_repressor	22239..22445	207	0.744	33.8	42	27.5	31.9	45
664	transcriptional_repressor	35221..35463	243	0.841	28.4	37	33.3	14.8	49.6
665	transcriptional_repressor	26773..27027	255	0.804	29.4	37.6	32.9	17.6	53.9
666	transcriptional_repressor	26773..27027	255	0.804	29.4	37.6	32.9	17.6	53.9
667	virion_structural_protein	8270..8605	336	0.731	39	53.6	36.6	26.8	43.8
668	virion_structural_protein	11073..11471	399	0.783	34.6	42.9	39.1	21.8	45.5
669	virion_structural_protein	6474..6923	450	0.756	38.7	52	40	24	43.1
670	XkdX_family_protein	15344..15490	147	0.709	32	38.8	26.5	30.6	34.9
671	XkdX_family_protein	15313..15459	147	0.737	31.3	38.8	28.6	26.5	27.6
672	XkdX_family_protein	19173..19319	147	0.745	30.6	38.8	26.5	26.5	39.8
673	XkdX_family_protein	27590..27736	147	0.763	30.6	38.8	26.5	26.5	32.4
674	XkdX_family_protein	19193..19339	147	0.797	29.3	38.8	26.5	22.4	41.2
675	XkdX_family_protein	19193..19339	147	0.797	29.3	38.8	26.5	22.4	41.2
676	YopX_family_protein	26346..26756	411	0.704	36.3	46	29.9	32.8	49.1
677	YopX_family_protein	35660..36085	426	0.691	36.6	46.5	28.2	35.2	45.1
678	YopX_family_protein	35660..36085	426	0.691	36.6	46.5	28.2	35.2	45.1
679	YopX_family_protein	2881..3324	444	0.701	34.7	45.3	27.7	31.1	49.3
680	YopX_family_protein	27194..27652	459	0.635	40.3	45.1	34.6	41.2	43.9
681	YopX_family_protein	33852..34313	462	0.672	37.4	40.3	34.4	37.7	49.7
682	YopX_family_protein	26069..26617	549	0.696	35.2	47.5	25.1	32.8	41.6
683	zinc-ribbon_domain-containing_protein	complement(25590..25808)	219	0.731	32.9	37	34.2	27.4	40.2
684	zinc-ribbon_domain-containing_protein	complement(26440..26658)	219	0.731	32.9	37	34.2	27.4	40.2
685	zinc-ribbon_domain-containing_protein	complement(34025..34243)	219	0.731	32.9	37	34.2	27.4	40.2
686	zinc-ribbon_domain-containing_protein	complement(25590..25808)	219	0.731	32.9	37	34.2	27.4	40.2

Table S5. The descriptive statistics of size, codon usage, GC content by codon position, and codon usage bias for the genes identified across the genomes of the Listeria virulent phages

No.	Gene	Gene coordinates in a genome	Gene length (nt)	CAI	%GC	%GC1	%GC2	%GC3	Nc
1	3D_domain-containing_protein	42141..42869	729	0.713	35	46.5	34.2	24.3	43.2
2	3D_domain-containing_protein	46314..47042	729	0.721	34.7	46.5	34.2	23.5	43.1
3	3D_domain-containing_protein	46314..47042	729	0.721	34.7	46.5	34.2	23.5	43.1
4	3D_domain-containing_protein	49645..50373	729	0.725	34.4	46.5	34.2	22.6	42.5
5	anti-sigma factor	49637..50227	591	0.638	36.9	44.2	28.4	38.1	47
6	anti-sigma_factor	92607..93212	606	0.636	36.8	43.6	28.2	38.6	47.1
7	anti-sigma_factor	83202..83792	591	0.64	36.7	44.2	28.4	37.6	47.4
8	anti-sigma_factor	85122..85712	591	0.64	36.7	44.2	28.4	37.6	47.4
9	anti-sigma_factor	89327..89917	591	0.641	36.7	44.2	28.4	37.6	47.6
10	anti-sigma_factor	89327..89917	591	0.641	36.7	44.2	28.4	37.6	47.6
11	anti-sigma_factor	complement(45275..45865)	591	0.64	36.7	44.2	28.4	37.6	47.9
12	baseplate J-like protein	30330..31376	1047	0.657	34.9	43.8	30.7	30.1	44.4
13	baseplate protein	29606..30316	711	0.677	35.6	50.2	31.6	24.9	44
14	baseplate_J-like_protein	65816..66862	1047	0.647	35.5	44.4	30.7	31.5	43.9
15	baseplate_J-like_protein	73316..74362	1047	0.657	34.9	43.8	30.7	30.1	44.4
16	baseplate_J-like_protein	63896..64942	1047	0.66	34.8	43.8	30.7	29.8	44.3
17	baseplate_J-like_protein	complement(64125..65171)	1047	0.665	34.8	44.4	30.7	29.2	43.3
18	baseplate_J-like_protein	70021..71067	1047	0.665	34.8	44.4	30.7	29.2	43.3
19	baseplate_J-like_protein	70021..71067	1047	0.665	34.8	44.4	30.7	29.2	43.3

20	baseplate_protein	63172..63882	711	0.674	35.7	50.2	31.2	25.7	44
21	baseplate_protein	65092..65802	711	0.676	35.7	50.2	31.6	25.3	44.4
22	baseplate_protein	complement(65185..65895)	711	0.682	35.6	50.2	31.2	25.3	45.1
23	baseplate_protein	69297..70007	711	0.682	35.6	50.2	31.2	25.3	45.1
24	baseplate_protein	69297..70007	711	0.682	35.6	50.2	31.2	25.3	45.1
25	baseplate_protein	72592..73302	711	0.677	35.6	50.2	31.6	24.9	44
26	CRISPR/Cas_system_associated	35834..36859	1026	0.727	38.6	48.8	37.4	29.5	43.9
27	deoxyuridine_5'-triphosphate_nucleotidohydrolase	complement(43520..44167)	648	0.698	38.9	48.1	41.2	27.3	42
28	DNA binding protein	66415..66729	315	0.61	34.3	46.7	26.7	29.5	46.1
29	DNA helicase	41690..43435	1746	0.686	35.5	46.6	30.8	29	47.2
30	DNA polymerase	21827..23833	2007	0.611	38.5	45	30.5	39.9	55.4
31	DNA polymerase	67983..70091	2109	0.655	36.9	47.7	30.6	32.4	45.4
32	DNA polymerase	66812..67648	837	0.664	36.1	49.5	30.1	28.7	47.1
33	DNA primase	23846..25753	1908	0.629	39.4	46.2	33.8	38.1	53.3
34	DNA primase/helicase	50227..51288	1062	0.65	36.3	47.7	31.1	30.2	44.1
35	DNA_binding_protein	31005..31319	315	0.691	40.6	53.3	29.5	39	50.2
36	DNA_binding_protein	106107..106421	315	0.61	34.3	46.7	26.7	29.5	46.1
37	DNA_binding_protein	109402..109716	315	0.61	34.3	46.7	26.7	29.5	46.1
38	DNA_binding_protein	106107..106421	315	0.61	34.3	46.7	26.7	29.5	46.1
39	DNA_binding_protein	100000..100314	315	0.632	33.3	46.7	26.7	26.7	42.7
40	DNA_binding_protein	complement(28742..29056)	315	0.64	33	46.7	26.7	25.7	45.5
41	DNA_binding_protein	101924..102238	315	0.646	32.7	46.7	26.7	24.8	46.5
42	DNA_helicase	81381..83126	1746	0.689	35.3	46.6	30.8	28.7	46.3
43	DNA_helicase	81381..83126	1746	0.689	35.3	46.6	30.8	28.7	46.3
44	DNA_helicase	84676..86421	1746	0.688	35.3	46.6	30.8	28.7	46.9
45	DNA_helicase	77176..78921	1746	0.693	34.9	46	30.6	28.2	45.3
46	DNA_helicase	complement(52066..53811)	1746	0.694	35	46.4	30.6	28	45.9
47	DNA_helicase	75256..77001	1746	0.703	34.8	46.4	30.6	27.3	45
48	DNA_polymerase	23285..25192	1908	0.58	41.3	44.5	30.7	48.7	58.3
49	DNA_polymerase	110886..113078	2193	0.65	37	46.8	30.8	33.5	45.9
50	DNA_polymerase	101484..103676	2193	0.653	37	46.9	30.5	33.5	45.4
51	DNA_polymerase	103995..106163	2169	0.65	37	46.7	30.8	33.3	46
52	DNA_polymerase	complement(24817..26985)	2169	0.649	37	47	30.7	33.3	46
53	DNA_polymerase	107615..109783	2169	0.652	36.9	46.9	31	32.8	45.4
54	DNA_polymerase	107615..109783	2169	0.652	36.9	46.9	31	32.8	45.4
55	DNA_polymerase	32111..33598	1488	0.676	37.9	51.2	31.7	30.8	50
56	DNA_polymerase	102320..103156	837	0.647	36.4	48.7	30.1	30.5	48.4
57	DNA_polymerase	100397..101233	837	0.652	36.4	49.1	29.7	30.5	47
58	DNA_polymerase	complement(27824..28660)	837	0.659	36.6	49.5	30.8	29.4	46.4
59	DNA_polymerase	109799..110635	837	0.661	36.1	49.5	30.1	28.7	47.1
60	DNA_polymerase	106504..107340	837	0.664	36.1	49.5	30.1	28.7	47.1
61	DNA_polymerase	106504..107340	837	0.664	36.1	49.5	30.1	28.7	47.1
62	DNA_polymerase	34522..35166	645	0.724	37.4	48.8	36.3	27	47.8
63	DNA_primase	25205..27109	1905	0.617	41.3	47.7	32.8	43.5	56.2
64	DNA_primase/helicase	complement(44214..45275)	1062	0.634	36.9	47.7	30.8	32.2	45
65	DNA_primase/helicase	85712..86773	1062	0.641	36.5	47.5	30.8	31.4	45
66	DNA_primase/helicase	93212..94273	1062	0.648	36.4	47.7	31.1	30.5	44.2
67	DNA_primase/helicase	89917..90978	1062	0.648	36.3	47.5	31.1	30.5	44.2
68	DNA_primase/helicase	89917..90978	1062	0.648	36.3	47.5	31.1	30.5	44.2
69	DNA_primase/helicase	83792..84853	1062	0.645	36.4	47.5	31.6	30.2	44.5
70	DNA_replication_inhibitor	39432..39656	225	0.678	33.8	45.3	20	36	49.4
71	DnaC-like_helicase_loader	28528..29310	783	0.707	37.3	42.5	36.8	32.6	48.9
72	DUF1768_domain-containing_protein	complement(22306..22809)	504	0.683	36.7	42.3	36.3	31.5	45.6
73	DUF1768_domain-containing_protein	complement(26444..26947)	504	0.688	36.9	43.5	36.3	31	47.8
74	DUF1768_domain-containing_protein	complement(29780..30283)	504	0.688	36.9	43.5	36.3	31	47.8
75	DUF1768_domain-containing_protein	complement(26444..26947)	504	0.688	36.9	43.5	36.3	31	47.8
76	DUF2829_domain-containing_protein	complement(36106..36381)	276	0.745	36.6	45.7	35.9	28.3	37.8
77	DUF2829_domain-containing_protein	complement(39440..39715)	276	0.745	36.6	45.7	35.9	28.3	37.8
78	DUF2829_domain-containing_protein	complement(36106..36381)	276	0.745	36.6	45.7	35.9	28.3	37.8
79	DUF4379_domain-containing_protein	complement(25021..25653)	633	0.631	37.6	45	35.5	32.2	45.9
80	DUF4379_domain-containing_protein	complement(21685..22317)	633	0.636	36.5	43.6	35.1	30.8	44.6
81	DUF4379_domain-containing_protein	complement(21685..22317)	633	0.636	36.5	43.6	35.1	30.8	44.6
82	DUF4379_domain-containing_protein	complement(17545..18177)	633	0.647	36	43.1	35.1	29.9	43.7
83	endolysin	19450..20325	876	0.647	41.8	45.9	37.7	41.8	51.5
84	endolysin	18076..19110	1035	0.695	37.9	44.3	38	31.3	45.9
85	endolysin	complement(89045..90070)	1026	0.729	40	45.6	46.5	27.8	41.9
86	endolysin	5472..6497	1026	0.732	39.8	45.3	46.5	27.5	44.5
87	endolysin	38983..40008	1026	0.732	39.7	45.3	46.2	27.5	44.8
88	endolysin	45116..46141	1026	0.736	39.7	45.3	46.5	27.2	44.3
89	endolysin	48447..49472	1026	0.736	39.7	45.3	46.5	27.2	44.3
90	endolysin	45116..46141	1026	0.736	39.7	45.3	46.5	27.2	44.3
91	endolysin	40943..41968	1026	0.739	39.6	45.6	46.5	26.6	43.9
92	endonuclease	complement(48576..49094)	519	0.731	34.3	48.6	28.3	26	41.5
93	exonuclease	47733..48113	381	0.641	32.3	39.4	24.4	33.1	54.4
94	exonuclease	90659..92602	1944	0.657	34.4	45.5	28.1	29.5	42.6
95	exonuclease	87424..89307	1884	0.663	34.3	46	27.7	29.1	40.6
96	exonuclease	87424..89307	1884	0.663	34.3	46	27.7	29.1	40.6
97	exonuclease	81299..83182	1884	0.667	34.5	46.7	27.9	29	40.8
98	exonuclease	83219..85102	1884	0.667	34.5	46.7	27.9	29	40.8
99	exonuclease	complement(45885..47768)	1884	0.67	34.4	46.7	27.9	28.8	40.8
100	exonuclease	116670..117941	1272	0.731	34.3	47.4	28.5	26.9	43.5
101	exonuclease	116670..117941	1272	0.731	34.3	47.4	28.5	26.9	43.5
102	exonuclease	110563..111834	1272	0.733	34.3	47.6	28.3	26.9	43.8
103	exonuclease	113070..114341	1272	0.736	34.2	47.2	28.8	26.7	43.8
104	exonuclease	119964..121235	1272	0.734	34.3	47.4	29	26.4	43.5
105	exonuclease	76977..78248	1272	0.734	34	47.4	28.3	26.2	43.9
106	exonuclease	complement(16547..17818)	1272	0.74	33.9	47.2	28.5	25.9	42.9
107	exopolyphosphatase	115682..116611	930	0.661	36.7	46.8	29	34.2	51.2
108	exopolyphosphatase	115682..116611	930	0.661	36.7	46.8	29	34.2	51.2
109	exopolyphosphatase	75989..76918	930	0.661	36.7	46.8	29	34.2	51.2
110	exopolyphosphatase	112082..113011	930	0.671	36.3	46.8	29.7	32.6	48.8
111	exopolyphosphatase	118976..119905	930	0.681	35.8	46.8	29.4	31.3	49
112	exopolyphosphatase	109575..110504	930	0.682	35.6	46.5	29	31.3	47.6
113	exopolyphosphatase	complement(17877..18806)	930	0.689	35.6	47.1	29	30.6	48.1
114	ferredoxin	4500..5447	948	0.71	40.5	49.4	42.4	29.7	45
115	flavodoxin	91066..91518	453	0.641	38.6	48.3	33.1	34.4	45
116	flavodoxin	97161..97613	453	0.632	38.2	47.7	32.5	34.4	44.3
117	flavodoxin	97161..97613	453	0.632	38.2	47.7	32.5	34.4	44.3
118	flavodoxin	complement(37548..38000)	453	0.651	39.1	50.3	33.1	33.8	45.1
119	flavodoxin	92986..93438	453	0.647	38.4	49	32.5	33.8	45.2
120	flavodoxin	57473..57925	453	0.635	37.7	47.7	31.8	33.8	43.9
121	flavodoxin	100456..100908	453	0.645	37.5	47.7	32.5	32.5	44.5
122	glycerophosphoryl diester phosphodiesterase	26798..28330	1533	0.65	36.6	43.4	35.4	30.9	47.2
123	glycerophosphoryl_diester_phosphodiesterase	69784..71316	1533	0.64	36.9	43.8	35.2	31.7	48.3
124	glycerophosphoryl_diester_phosphodiesterase	60364..61896	1533	0.65	36.7	43.6	35.4	30.9	47.4
125	glycerophosphoryl_diester_phosphodiesterase	66489..68021	1533	0.65	36.6	43.4	35.4	30.9	47.2
126	glycerophosphoryl_diester_phosphodiesterase	66489..68021	1533	0.65	36.6	43.4	35.4	30.9	47.2
127	glycerophosphoryl_diester_phosphodiesterase	complement(67171..68703)	1533	0.646	36.6	43.6	35.2	30.9	46.9
128	glycerophosphoryl_diester_phosphodiesterase	62284..63816	1533	0.654	36.6	43.8	35.6	30.3	47.4
129	gp1	29..649	621	0.552	44.4	52.2	35.7	45.4	50.4
130	gp1	20..781	762	0.576	44.5	54.7	35	43.7	54.2
131	gp1	264..551	288	0.643	35.1	43.8	28.1	33.3	47.4
132	gp10	3103..3249	147	0.54	38.1	40.8	24.5	49	44.2
133	gp100	90595..91128	534	0.678	36	47.2	31.5	29.2	50.1
134	gp101	62652..63185	534	0.661	32.4	40.4	28.1	28.7	41.4
135	gp101	91143..91901	759	0.647	34.8	48.2	29.2	26.9	38
136	gp102	91914..92204	291	0.636	34.4	37.1	28.9	37.1	45.1
137	gp103	complement(92633..92914)	282	0.597	37.9	41.5	33	39.4	61
138	gp104	93942..94724	783	0.718	32.1	43.7	24.1	28.4	45.1
139	gp105	94891..95100	210	0.656	36.2	48.6	22.9	37.1	28.1
140	gp106	95213..95476	264	0.793	33.3	42	28.4	29.5	45.1
141	gp106	69562..73017	3456	0.72	38.3	48.1	39.7	27	42

142	gp107	95560..95835	276	0.711	32.2	33.7	30.4	32.6	48.3
143	gp107	73063..73284	222	0.597	34.2	47.3	24.3	31.1	47.4
144	gp108	73287..74579	1293	0.677	39	44.8	41.8	30.4	48.1
145	gp109	97353..97607	255	0.641	33.3	41.2	28.2	30.6	41
146	gp109	74611..75021	411	0.686	37	53.3	31.4	26.3	40.9
147	gp11	3335..3745	411	0.622	40.6	52.6	32.1	37.2	43.2
148	gp11	6670..7398	729	0.722	34.4	46.5	34.2	22.6	41.7
149	gp110	97604..97864	261	0.692	36	47.1	24.1	36.8	55.9
150	gp110	75018..75155	138	0.728	33.3	41.3	32.6	26.1	41.1
151	gp111	97888..98073	186	0.641	29	27.4	24.2	35.5	41
152	gp112	98092..98265	174	0.7	28.7	34.5	31	20.7	32.8
153	gp113	98407..98682	276	0.687	34.1	44.6	28.3	29.3	46.3
154	gp114	98696..98983	288	0.729	31.2	42.7	29.2	21.9	42.2
155	gp115	99137..99409	273	0.648	37.4	45.1	30.8	36.3	42.6
156	gp116	99726..99854	129	0.686	31.8	48.8	20.9	25.6	34.7
157	gp117	100157..100561	405	0.636	39.5	51.1	31.1	36.3	46.5
158	gp118	100564..100782	219	0.672	38.4	42.5	42.5	30.1	38
159	gp118	84900..85547	648	0.696	38.4	46.8	41.2	27.3	41.3
160	gp119	100784..101005	222	0.619	40.1	56.8	29.7	33.8	38
161	gp119	85544..85768	225	0.667	32.4	42.7	30.7	24	55.1
162	gp12	9371..9808	438	0.705	37	47.9	27.4	35.6	37.3
163	gp12	3735..3920	186	0.692	44.1	59.7	37.1	35.5	43.8
164	gp12	9363..9821	459	0.687	35.7	49	24.2	34	50.9
165	gp12	7500..7820	321	0.689	31.5	42.1	26.2	26.2	43.4
166	gp120	101012..101248	237	0.669	32.5	38	24.1	35.4	42.8
167	gp120	85765..86088	324	0.722	33.3	47.2	27.8	25	36.9
168	gp121	101245..101511	267	0.654	37.5	52.8	25.8	33.7	33.2
169	gp121	86081..86503	423	0.724	34.8	53.2	27	24.1	47.9
170	gp122	101504..101995	492	0.643	36	43.9	34.8	29.3	45.6
171	gp123	101998..102504	507	0.648	37.5	50.9	29.6	32	48.5
172	gp124	102515..103699	1185	0.678	35.5	42.5	33.9	30.1	49.9
173	gp125	103862..104287	426	0.68	34.7	46.5	26.8	31	41.8
174	gp126	104305..104595	291	0.703	37.5	57.7	25.8	28.9	42.1
175	gp127	104592..104777	186	0.654	36	46.8	24.2	37.1	60
176	gp127	91521..91817	297	0.729	36.4	57.6	31.3	20.2	39.8
177	gp128	91841..92152	312	0.632	34.6	46.2	26	31.7	45.4
178	gp128	104777..105124	348	0.728	33.6	40.5	33.6	26.7	44.5
179	gp129	105156..105416	261	0.652	37.5	44.8	29.9	37.9	60.4
180	gp13	3922..4143	222	0.628	40.5	62.2	25.7	33.8	48
181	gp130	92537..92674	138	0.729	32.6	47.8	23.9	26.1	37.1
182	gp130	105496..105828	333	0.732	35.1	46.8	34.2	24.3	39.9
183	gp131	92677..93864	1188	0.653	37.4	46	36.4	29.8	43.7
184	gp131	105829..106224	396	0.692	31.1	34.1	32.6	26.5	47.6
185	gp132	106289..106468	180	0.671	34.4	48.3	33.3	21.7	50.4
186	gp133	106491..106853	363	0.621	33.6	49.6	20.7	30.6	55.1
187	gp134	106853..107209	357	0.666	34.7	38.7	30.3	35.3	36.4
188	gp134	96666..99125	2460	0.668	36.3	45	32.1	31.8	45.6
189	gp135	99219..100007	789	0.643	35	43	30.4	31.6	49.3
190	gp136	complement(108039..108359)	321	0.664	36.4	42.1	34.6	32.7	47.4
191	gp137	complement(108352..108660)	309	0.624	38.5	46.6	29.1	39.8	54.4
192	gp138	complement(108674..109183)	510	0.72	30.2	40	28.8	21.8	38.5
193	gp139	103771..104247	477	0.645	36.5	51.6	23.9	34	45.4
194	gp139	complement(109205..109396)	192	0.742	26.6	34.4	25	20.3	38.2
195	gp14	10160..12046	1887	0.637	44.6	51.8	38.6	43.2	55.1
196	gp14	10153..12039	1887	0.655	44	53.3	38.8	39.9	50.2
197	gp14	4150..4263	114	0.73	30.7	34.2	31.6	26.3	32.1
198	gp140	complement(109402..109686)	285	0.739	35.1	57.9	24.2	23.2	53.6
199	gp141	complement(109876..110157)	282	0.654	36.2	43.6	35.1	29.8	35.2
200	gp142	complement(110217..110441)	225	0.713	32.4	40	26.7	30.7	49.9
201	gp142	106920..107297	378	0.724	34.4	41.3	34.9	27	41.3
202	gp143	complement(110442..110984)	543	0.628	36.1	50.3	24.3	33.7	44.4
203	gp144	complement(110981..111208)	228	0.574	39.9	47.4	30.3	42.1	44.4
204	gp145	complement(111211..112464)	1254	0.685	35.6	46.7	30.4	29.9	45.8
205	gp146	complement(112466..112891)	426	0.595	35.9	43	24.6	40.1	51
206	gp146	109192..109578	387	0.651	36.4	49.6	26.4	33.3	42.4
207	gp147	complement(112956..113444)	489	0.671	36.4	42.3	36.2	30.7	48.3
208	gp148	complement(113450..114082)	633	0.647	36	43.6	35.1	29.4	44.3
209	gp149	111854..112237	384	0.722	36.5	49.2	34.4	25.8	43.6
210	gp15	4442..4942	501	0.64	34.7	40.1	32.9	31.1	46.1
211	gp150	complement(114272..114784)	513	0.653	38.8	38.6	41.5	36.3	51.8
212	gp150	112245..112790	546	0.641	40.8	48.9	41.2	32.4	44.1
213	gp151	112845..113042	198	0.66	37.9	42.4	36.4	34.8	49.7
214	gp152	113093..113800	708	0.65	34.9	42.8	33.1	28.8	47.3
215	gp152	complement(115478..115696)	219	0.777	34.2	50.7	32.9	19.2	37.9
216	gp153	113811..114293	483	0.693	35.8	40.4	40.4	26.7	38.8
217	gp154	114353..115237	885	0.657	35.9	42	33.6	32.2	43.9
218	gp155	115326..115763	438	0.708	37.7	46.6	34.9	31.5	40.1
219	gp156	115769..116215	447	0.633	36.7	46.3	28.9	34.9	51.6
220	gp157	116316..117020	705	0.61	35.6	43	31.1	32.8	44.1
221	gp157	complement(118015..118206)	192	0.694	30.7	32.8	31.2	28.1	45.4
222	gp158	complement(118207..118710)	504	0.695	36.5	43.5	36.3	29.8	46
223	gp159	118028..118873	846	0.659	33.8	41.8	30.9	28.7	47.3
224	gp159	complement(118712..118981)	270	0.756	31.9	41.1	34.4	20	45.4
225	gp16	4945..5451	507	0.64	36.3	48.5	27.8	32.5	48.7
226	gp160	118935..119390	456	0.672	37.9	53.9	31.6	28.3	42.9
227	gp161	119396..119974	579	0.677	38.2	51.3	32.6	30.6	42.3
228	gp161	complement(120344..120547)	204	0.7	30.4	36.8	27.9	26.5	47.1
229	gp162	120013..120585	573	0.655	33.7	40.3	30.9	29.8	49.1
230	gp163	120582..121160	579	0.66	35.8	47.2	31.6	28.5	48.7
231	gp163	complement(120985..121209)	225	0.751	28.9	40	28	18.7	33.5
232	gp164	complement(121223..121465)	243	0.671	33.7	40.7	30.9	29.6	36.3
233	gp164	121153..121485	333	0.709	31.5	35.1	30.6	28.8	37
234	gp165	121771..122499	729	0.663	35.5	44.4	30.9	31.3	46
235	gp166	complement(123801..124019)	219	0.652	35.6	46.6	28.8	31.5	48.7
236	gp167	complement(125090..125497)	408	0.602	33.3	34.6	33.8	31.6	41.8
237	gp167	123096..124166	1071	0.674	37.3	52.4	30.8	28.6	44.7
238	gp168	complement(125523..125720)	198	0.682	35.4	48.5	25.8	31.8	29.9
239	gp168	124214..124783	570	0.69	33.7	46.8	28.4	25.8	47.3
240	gp169	complement(127855..128130)	276	0.745	36.6	45.7	35.9	28.3	37.8
241	gp169	124786..125319	534	0.688	35.8	47.2	32.6	27.5	45.8
242	gp17	5461..6645	1185	0.68	36.1	42.5	35.2	30.6	48.8
243	gp17	10718..11620	903	0.722	36.1	53.2	30.2	24.9	40.8
244	gp170	complement(128254..128679)	426	0.629	34	39.4	29.6	33.1	42.9
245	gp170	125334..126092	759	0.661	35	48.2	30	26.9	40
246	gp171	126105..126395	291	0.652	34.4	39.2	28.9	35.1	48.1
247	gp171	complement(130039..130275)	237	0.77	32.1	46.8	34.2	15.2	37
248	gp172	127419..127778	360	0.808	30	40	24.2	25.8	46.4
249	gp173	127863..128012	150	0.769	36.7	44	32	34	44.3
250	gp173	complement(130691..131035)	345	0.685	34.8	46.1	26.1	32.2	48.5
251	gp174	128121..128384	264	0.786	33.7	42	27.3	31.8	43.8
252	gp174	complement(131051..131320)	270	0.673	33.7	43.3	28.9	28.9	42.7
253	gp175	128468..128743	276	0.751	29.3	31.5	29.3	27.2	44.5
254	gp176	128819..129271	453	0.742	31.1	37.1	29.1	27.2	39.3
255	gp177	129273..129368	96	0.683	28.1	31.2	18.8	34.4	29.1
256	gp179	130096..130323	228	0.694	26.3	32.9	22.4	23.7	61
257	gp18	6798..7223	426	0.654	35.4	45.8	27.5	33.1	43.9
258	gp18	13278..13613	336	0.738	28.6	40.2	25	20.5	43
259	gp180	130397..130594	198	0.811	29.3	40.9	24.2	22.7	43.2
260	gp181	130613..130819	207	0.707	38.2	53.6	27.5	33.3	50
261	gp182	130823..131098	276	0.689	34.4	38	32.6	32.6	33.2
262	gp183	131225..131527	303	0.693	35	46.5	26.7	31.7	41.1
263	gp184	131599..131841	243	0.715	33.7	54.3	21	25.9	53.3
264	gp185	131924..132067	144	0.713	33.3	41.7	27.1	31.2	47.6

265	gp186	132270..132611	342	0.737	35.4	46.5	28.9	30.7	36.3
266	gp187	132786..133007	222	0.727	36	45.9	28.4	33.8	35.4
267	gp188	133013..133456	444	0.714	33.8	43.9	31.1	26.4	43.3
268	gp189	133589..134131	543	0.634	35.9	45.3	29.8	32.6	51.3
269	gp19	16299..16544	246	0.588	32.9	34.1	23.2	41.5	49.5
270	gp19	7241..7531	291	0.638	39.5	56.7	25.8	36.1	53.4
271	gp19	15947..16096	150	0.7	35.3	38	36	32	61
272	gp190	134156..134434	279	0.647	35.8	43	33.3	31.2	38.7
273	gp2	717..971	255	0.671	33.3	42.4	29.4	28.2	43.4
274	gp2	422..694	273	0.69	35.9	37.4	46.2	24.2	49.9
275	gp20	16305..16592	288	0.562	34	38.5	22.9	40.6	61
276	gp20	7528..7713	186	0.682	34.4	46.8	24.2	32.3	53.6
277	gp20	16660..16905	246	0.758	33.7	45.1	29.3	26.8	33.4
278	gp21	16780..16971	192	0.62	33.9	35.9	20.3	45.3	46.9
279	gp21	16905..17117	213	0.683	35.2	46.5	23.9	35.2	46.5
280	gp21	7713..8060	348	0.699	35.9	42.2	34.5	31	48.3
281	gp21	15337..15954	618	0.685	34.5	36.4	39.3	27.7	45.7
282	gp22	17119..17448	330	0.677	40.9	42.7	39.1	40.9	44.1
283	gp22	8092..8373	282	0.661	36.2	41.5	33	34	53.4
284	gp22	16937..17317	381	0.657	31.5	38.6	24.4	31.5	45.5
285	gp22	15967..16806	840	0.679	35.2	43.9	34.6	27.1	43.7
286	gp23	17317..17457	141	0.564	36.9	34	25.5	51.1	61
287	gp23	17448..18221	774	0.618	41.6	53.9	34.5	36.4	54.4
288	gp23	8452..8784	333	0.701	36	45	35.1	27.9	38.4
289	gp23	16806..17126	321	0.712	33.6	52.3	25.2	23.4	45.1
290	gp24	17764..17919	156	0.575	38.5	48.1	30.8	36.5	42.9
291	gp24	18218..18460	243	0.687	37.9	39.5	39.5	34.6	56.1
292	gp24	8884..9180	297	0.657	33	39.4	32.3	27.3	39.9
293	gp25	18444..19067	624	0.62	42	49	35.6	41.3	54.3
294	gp25	17927..18085	159	0.703	33.3	37.7	30.2	32.1	40
295	gp25	9245..9424	180	0.679	33.9	48.3	33.3	20	48.8
296	gp26	19103..19261	159	0.57	42.8	50.9	28.3	49.1	43.5
297	gp26	9447..9809	363	0.616	35	51.2	23.1	30.6	52.9
298	gp27	19272..19433	162	0.66	33.3	31.5	31.5	37	61
299	gp27	9809..10165	357	0.634	35.9	42	30.3	35.3	38.9
300	gp27	19265..19627	363	0.644	36.1	44.6	29.8	33.9	52.8
301	gp29	20439..20834	396	0.624	38.4	43.2	28	43.9	53.5
302	gp29	20268..21017	750	0.6	39.2	48	29.2	40.4	51
303	gp29	complement(10995..11315)	321	0.653	37.4	44.9	34.6	32.7	44.6
304	gp3	971..1228	258	0.683	34.9	48.8	19.8	36	42.3
305	gp30	complement(11308..11478)	171	0.573	36.8	38.6	28.1	43.9	56.5
306	gp30	21038..21742	705	0.677	36.2	50.6	23.4	34.5	44.8
307	gp31	21497..22243	747	0.563	39.9	47	26.5	46.2	58.2
308	gp31	complement(11522..12049)	528	0.63	35.2	44.9	25.6	35.2	50.4
309	gp32	complement(12046..12327)	282	0.648	36.5	44.7	34	30.9	37.1
310	gp32	29086..29619	534	0.659	32.6	40.4	28.7	28.7	41.9
311	gp32	22263..22958	696	0.789	34.1	49.6	26.7	25.9	40.1
312	gp33	complement(12387..12611)	225	0.69	31.1	36	26.7	30.7	46.3
313	gp33	25754..25999	246	0.726	30.9	36.6	29.3	26.8	38.7
314	gp34	25983..26411	429	0.632	37.3	47.6	28	36.4	61
315	gp34	complement(12612..13154)	543	0.604	36.1	49.7	23.2	35.4	44.3
316	gp35	complement(13151..13378)	228	0.599	38.6	47.4	28.9	39.5	45.6
317	gp35	27102..27341	240	0.636	37.9	43.8	33.8	36.2	50.8
318	gp35	26526..27065	540	0.689	33.9	45	23.3	33.3	61
319	gp36	27062..27247	186	0.593	39.2	48.4	33.9	35.5	61
320	gp36	27437..27652	216	0.708	33.3	45.8	20.8	33.3	43
321	gp36	complement(13381..14631)	1251	0.676	35	44.6	30.2	30.2	46
322	gp37	complement(14642..15055)	414	0.61	35.7	42.8	25.4	39.1	49.3
323	gp37	27649..28203	555	0.658	40.4	50.3	33	37.8	57
324	gp37	27226..27447	222	0.676	34.7	40.5	33.8	29.7	49.5
325	gp37	35996..39451	3456	0.718	38.3	47.8	39.6	27.4	41.9
326	gp38	39497..39718	222	0.597	34.2	47.3	24.3	31.1	47.4
327	gp38	complement(15121..15609)	489	0.71	35.4	43.6	36.2	26.4	48.3
328	gp39	28233..28673	441	0.608	38.1	44.2	27.2	42.9	61
329	gp39	28784..29215	432	0.641	37.7	41.7	31.2	40.3	50.6
330	gp39	39922..41013	1092	0.669	39.8	45.3	43.4	30.8	47.8
331	gp39	complement(15615..16247)	633	0.647	36.8	44.5	36	29.9	47.5
332	gp4	1252..1437	186	0.646	28	25.8	24.2	33.9	37
333	gp40	28663..28857	195	0.648	41	50.8	32.3	40	55.2
334	gp40	29290..29490	201	0.661	36.3	44.8	28.4	35.8	45.9
335	gp40	41045..41455	411	0.667	37.5	52.6	31.4	28.5	40.9
336	gp41	29487..29957	471	0.625	43.3	46.5	38.9	44.6	47.2
337	gp41	28854..29384	531	0.611	40.1	47.5	32.2	40.7	60.7
338	gp41	complement(16437..16856)	420	0.682	37.1	37.1	42.9	31.4	49.2
339	gp41	41452..41589	138	0.728	33.3	41.3	32.6	26.1	41.1
340	gp42	29957..30172	216	0.641	40.3	48.6	33.3	38.9	49.4
341	gp42	complement(16853..17038)	186	0.648	35.5	29	41.9	35.5	53.2
342	gp42	29374..29925	552	0.68	38.6	48.9	31.5	35.3	57.6
343	gp43	30183..30644	462	0.666	38.5	50.6	26.6	38.3	56.3
344	gp43	29926..30135	210	0.647	38.1	47.1	31.4	35.7	48.2
345	gp44	30136..30540	405	0.637	37.8	44.4	30.4	38.5	61
346	gp44	30647..31012	366	0.686	37.7	45.1	33.6	34.4	40
347	gp44	complement(17643..17861)	219	0.775	32.4	47.9	32.9	16.4	32.1
348	gp45	30537..30707	171	0.698	36.3	43.9	33.3	31.6	32.9
349	gp46	31316..31624	309	0.616	38.2	52.4	26.2	35.9	49.9
350	gp46	30685..30843	159	0.622	34	32.1	34	35.8	40
351	gp46	complement(18258..18614)	357	0.679	35.9	45.4	29.4	32.8	37.7
352	gp47	30869..31150	282	0.717	33	44.7	21.3	33	50.8
353	gp47	31617..31799	183	0.615	36.1	44.3	31.1	32.8	31.7
354	gp48	31908..32012	105	0.51	38.1	34.3	31.4	48.6	54.3
355	gp48	31175..31447	273	0.741	30.8	37.4	29.7	25.3	58.5
356	gp49	32009..32320	312	0.637	39.4	48.1	29.8	40.4	51.5
357	gp49	complement(20179..20370)	192	0.682	31.2	32.8	31.2	29.7	46.7
358	gp49	31611..31886	276	0.756	33	52.2	22.8	23.9	33.8
359	gp5	4960..5556	597	0.67	39.5	56.8	29.1	32.7	50
360	gp5	4918..5499	582	0.663	40.7	57.7	32.5	32	49.2
361	gp5	1455..1628	174	0.699	31	36.2	31	25.9	43.3
362	gp50	32298..32465	168	0.594	37.5	41.1	32.1	39.3	61
363	gp50	31893..32024	132	0.658	31.1	34.1	27.3	31.8	46.2
364	gp50	complement(20371..20874)	504	0.689	36.5	42.3	36.3	31	47.4
365	gp50	51335..51982	648	0.697	39.2	48.6	41.2	27.8	41
366	gp51	32449..33357	909	0.61	41	48.2	32.7	42.2	56.1
367	gp51	32027..32230	204	0.732	39.2	47.1	38.2	32.4	48.9
368	gp51	51979..52203	225	0.637	34.7	45.3	30.7	28	47.3
369	gp51	complement(20876..21145)	270	0.75	32.2	42.2	34.4	20	42.5
370	gp52	32227..32376	150	0.567	35.3	38	30	38	59.6
371	gp52	33357..33548	192	0.661	39.6	45.3	39.1	34.4	48
372	gp52	52200..52523	324	0.725	33	46.3	26.9	25.9	34.7
373	gp53	33550..34179	630	0.628	37.9	44.3	28.6	41	51.4
374	gp53	32373..32588	216	0.681	33.8	40.3	22.2	38.9	43.2
375	gp53	complement(22509..22712)	204	0.696	30.4	35.3	27.9	27.9	51.7
376	gp53	52516..52938	423	0.707	35.5	53.2	27	26.2	50.1
377	gp54	34270..34674	405	0.597	40	49.6	29.6	40.7	53.7
378	gp54	32578..32829	252	0.702	35.7	51.2	27.4	28.6	49.9
379	gp55	34677..35090	414	0.549	46.6	50.7	35.5	53.6	59.2
380	gp55	32810..33103	294	0.648	34	38.8	27.6	35.7	47.6
381	gp55	complement(23150..23374)	225	0.748	29.3	41.3	28	18.7	40.7
382	gp56	35063..35326	264	0.572	47.7	53.4	37.5	52.3	60.7
383	gp56	33075..33371	297	0.674	36.7	46.5	28.3	35.4	43.5
384	gp56	complement(23388..23630)	243	0.696	32.5	40.7	29.6	27.2	36.1
385	gp57	33368..33652	285	0.599	37.5	41.1	28.4	43.2	46.7
386	gp58	33737..34504	768	0.57	42.8	50.4	33.2	44.9	52.8

387	gp59	34520..34705	186	0.598	40.3	46.8	30.6	43.5	32.6
388	gp59	complement(25924..26142)	219	0.659	36.1	49.3	28.8	30.1	39.1
389	gp59	57928..58224	297	0.736	36	57.6	31.3	19.2	38.4
390	gp6	1770..2045	276	0.634	35.5	44.6	28.3	33.7	51.4
391	gp60	34660..34896	237	0.635	37.1	43	29.1	39.2	61
392	gp60	complement(27214..27621)	408	0.601	33.1	34.6	33.1	31.6	41.5
393	gp61	34889..35062	174	0.682	35.1	39.7	29.3	36.2	48.1
394	gp61	complement(27647..27844)	198	0.682	35.4	48.5	25.8	31.8	29.9
395	gp61	58943..59080	138	0.749	31.2	45.7	23.9	23.9	41.2
396	gp62	28039..28203	165	0.521	47.3	40	47.3	54.5	43.3
397	gp62	35366..35527	162	0.579	35.8	33.3	38.9	35.2	60.2
398	gp62	59083..60270	1188	0.649	37.5	45.7	36.6	30.1	43.6
399	gp63	29045..29299	255	0.539	40	31.8	43.5	44.7	43.6
400	gp64	complement(29979..30254)	276	0.745	36.6	45.7	35.9	28.3	37.8
401	gp65	complement(30378..30803)	426	0.629	34	39.4	29.6	33.1	42.9
402	gp65	63081..65540	2460	0.678	35.5	44.6	31.1	30.7	44.2
403	gp66	65634..66422	789	0.643	35.1	43.7	30	31.6	51.5
404	gp66	complement(32166..32402)	237	0.77	32.1	46.8	34.2	15.2	37
405	gp68	complement(32818..33162)	345	0.685	34.8	46.1	26.1	32.2	48.5
406	gp69	complement(33178..33447)	270	0.662	33.3	42.2	28.9	28.9	42.7
407	gp7	2059..2346	288	0.72	32.3	41.7	30.2	25	49
408	gp7	4213..4410	198	0.662	29.3	36.4	27.3	24.2	34.3
409	gp70	70186..70662	477	0.643	36.7	51.6	24.5	34	45.3
410	gp71	33933..34205	273	0.68	36.6	37.4	46.2	26.4	53
411	gp73	73335..73712	378	0.744	33.6	41.3	34.9	24.6	41.7
412	gp75	74409..74570	162	0.782	35.2	66.7	16.7	22.2	42.1
413	gp76	complement(74626..74769)	144	0.685	36.1	33.3	43.8	31.2	30.9
414	gp76	37724..37921	198	0.662	29.3	36.4	27.3	24.2	34.3
415	gp77	37911..38549	639	0.662	36.9	50.7	30	30	51.3
416	gp78	75606..75992	387	0.652	37	49.6	27.1	34.1	41.9
417	gp78	38539..38919	381	0.667	34.6	42.5	31.5	29.9	39.8
418	gp8	2499..2771	273	0.633	37.7	46.2	30.8	36.3	40.3
419	gp8	4400..5038	639	0.662	36.9	50.7	30	30	51.3
420	gp80	40181..40909	729	0.722	34.4	46.5	34.2	22.6	41.7
421	gp81	78268..78651	384	0.706	37.5	50	34.4	28.1	44.4
422	gp81	41011..41331	321	0.694	31.5	42.1	26.2	26.2	43.2
423	gp82	78659..79204	546	0.642	41	49.5	41.2	32.4	45.3
424	gp83	79259..79456	198	0.674	36.9	42.4	36.4	31.8	50.7
425	gp84	79507..80214	708	0.642	35.5	42.4	33.5	30.5	45.3
426	gp85	80225..80707	483	0.665	36.6	40.4	40.4	29.2	41.1
427	gp85	44229..45122	894	0.724	35.7	52	30.9	24.2	41.6
428	gp86	80767..81651	885	0.663	35.4	41.4	33.2	31.5	43.2
429	gp87	81740..82177	438	0.708	37.7	46.6	34.9	31.5	40.1
430	gp87	46777..47172	396	0.701	29.8	43.2	24.2	22	41.7
431	gp88	82183..82629	447	0.641	36.2	46.3	28.9	33.6	49
432	gp89	82604..83434	831	0.602	35.7	40.1	32.9	34.3	43.5
433	gp9	7409..7834	426	0.558	45.5	49.3	40.1	47.2	61
434	gp9	7394..7858	465	0.602	43.4	47.7	36.8	45.8	50.2
435	gp9	5028..5408	381	0.667	34.6	42.5	31.5	29.9	39.8
436	gp9	2904..3032	129	0.697	31	48.8	20.9	23.3	31.1
437	gp90	48896..49513	618	0.683	35	37.4	39.3	28.2	45.4
438	gp91	84442..85287	846	0.664	33.8	42.6	31.6	27.3	46
439	gp91	49526..50365	840	0.684	35.1	44.3	34.3	26.8	44
440	gp92	85349..85816	468	0.658	38.5	54.5	31.4	29.5	44.6
441	gp92	50365..50685	321	0.709	34	52.3	25.2	24.3	45.7
442	gp93	85849..86421	573	0.669	33.2	41.9	29.8	27.7	47
443	gp94	86418..86996	579	0.658	36.6	47.2	32.1	30.6	43.9
444	gp95	86989..87321	333	0.719	31.8	33.3	34.2	27.9	32.9
445	gp96	87607..88335	729	0.67	34.8	44	30.5	30	43.2
446	gp98	88932..89975	1044	0.677	37.4	52.6	29.9	29.6	47.2
447	gp99	90023..90592	570	0.682	34	47.9	28.9	25.3	46.8
448	head maturation protease	9931..10725	795	0.713	37.1	47.5	35.5	28.3	39.2
449	head morphogenesis	3930..4841	912	0.595	43.3	51.3	34.9	43.8	52.6
450	head protein	7855..8322	468	0.626	41.2	51.9	34	37.8	54.4
451	head protein	6543..7079	537	0.667	39.7	48	34.6	36.3	51.9
452	head_maturation_protease	45403..46197	795	0.712	37.2	47.5	35.5	28.7	39.9
453	head_maturation_protease	52907..53701	795	0.714	37.2	47.5	35.5	28.7	40.1
454	head_maturation_protease	43442..44236	795	0.717	37.2	47.9	35.5	28.3	40
455	head_maturation_protease	49576..50370	795	0.717	37	47.2	35.5	28.3	39.6
456	head_maturation_protease	49576..50370	795	0.717	37	47.2	35.5	28.3	39.6
457	head_maturation_protease	complement(84804..85598)	795	0.721	37	47.5	35.5	27.9	40.1
458	head_morphogenesis	8548..9333	786	0.71	35.6	47.3	32.4	27.1	44.7
459	head_protein	7834..8298	465	0.57	41.7	47.7	29	48.4	55.9
460	head_protein	6518..7054	537	0.642	41.7	50.8	33	41.3	54.3
461	head_scaffolding_protein	9403..10044	642	0.724	39.3	56.5	35	26.2	39.5
462	head-tail adaptor	7072..7419	348	0.652	39.1	44	31	42.2	51.7
463	head-tail_adaptor	7054..7407	354	0.608	42.7	49.2	34.7	44.1	51.8
464	head-tail_connector	12213..12572	360	0.645	36.7	51.7	29.2	29.2	44.2
465	helicase	29311..30639	1329	0.742	37.1	49.2	34.3	27.8	41.8
466	helicase DnaB-like	45108..46571	1464	0.701	35.5	49.6	29.3	27.7	43.7
467	helicase_DnaB-like	84799..86262	1464	0.692	35.8	49.6	29.3	28.5	43.2
468	helicase_DnaB-like	84799..86262	1464	0.692	35.8	49.6	29.3	28.5	43.2
469	helicase_DnaB-like	88094..89557	1464	0.694	35.5	49	29.3	28.3	42.8
470	helicase_DnaB-like	78674..80137	1464	0.701	35.5	49.4	29.3	27.7	43.7
471	helicase_DnaB-like	80594..82057	1464	0.704	35.5	49.4	29.3	27.7	44
472	helicase_DnaB-like	complement(48930..50393)	1464	0.704	35.3	49.4	29.3	27.3	43.4
473	helix-turn-helix_XRE-family-like_protein	complement(29464..29706)	243	0.659	33.7	42	29.6	29.6	35.8
474	helix-turn-helix_XRE-family-like_protein	complement(32799..33041)	243	0.659	33.7	42	29.6	29.6	35.8
475	helix-turn-helix_XRE-family-like_protein	complement(29464..29706)	243	0.659	33.7	42	29.6	29.6	35.8
476	helix-turn-helix_XRE-family-like_protein	complement(25326..25568)	243	0.664	33.7	42	29.6	29.6	40.1
477	hemolysin	96260..96601	342	0.671	34.8	54.4	21.9	28.1	47.3
478	hemolysin	complement(32452..32793)	342	0.682	34.2	54.4	21.9	26.3	47
479	hemolysin	complement(38585..38926)	342	0.682	34.2	54.4	21.9	26.3	47
480	hemolysin	complement(34412..34753)	342	0.682	34.2	54.4	21.9	26.3	47
481	hemolysin	complement(41916..42257)	342	0.682	34.2	54.4	21.9	26.3	47
482	hemolysin	complement(38585..38926)	342	0.682	34.2	54.4	21.9	26.3	47
483	hemolysin	complement(130325..130666)	342	0.682	34.2	54.4	21.9	26.3	47
484	HNH_endonuclease	complement(59485..59796)	312	0.649	38.8	48.1	34.6	33.7	44.4
485	HNH_endonuclease	complement(61319..62242)	924	0.671	35.7	44.2	32.8	30.2	51.2
486	HNH_endonuclease	35222..35833	612	0.715	36.8	46.1	34.8	29.4	48.3
487	HNH_endonuclease	complement(27211..27708)	498	0.666	35.5	41	36.7	28.9	42
488	HNH_endonuclease	103272..103769	498	0.666	35.5	41	36.7	28.9	42
489	HNH_endonuclease	33715..34218	504	0.686	35.3	41.1	36.3	28.6	43.4
490	HNH_endonuclease	complement(62454..62849)	396	0.714	35.9	43.9	41.7	22	46.8
491	HNH_endonuclease	27553..27924	372	0.769	30.6	42.7	32.3	16.9	38.5
492	holin	15427..15876	450	0.624	39.8	48	30.7	40.7	57.6
493	holin	15487..15942	456	0.648	37.5	42.8	32.2	37.5	46.7
494	holin	4216..4500	285	0.721	39.6	42.1	44.2	32.6	46.6
495	holin	117395..117760	366	0.733	35.8	51.6	27.9	27.9	39.4
496	holin	114101..114466	366	0.753	35.8	53.3	27.9	26.2	37.5
497	holin	114101..114466	366	0.753	35.8	53.3	27.9	26.2	37.5
498	holin	107994..108359	366	0.748	35.2	51.6	27.9	26.2	37.5
499	holin	110486..110866	381	0.746	35.2	52.8	26.8	26	38.1
500	HTH DNA binding protein	43450..45090	1641	0.688	36.7	47.5	33.3	29.3	45.4
501	HTH_DNA_binding_protein	78936..80576	1641	0.682	36.7	47.2	33.1	30	45.6
502	HTH_DNA_binding_protein	complement(50411..52024)	1614	0.685	36.6	47.2	33.1	29.4	43.4
503	HTH_DNA_binding_protein	86436..88076	1641	0.693	36.3	47.5	33.1	28.3	44.1
504	HTH_DNA_binding_protein	83141..84781	1641	0.693	36.2	47.2	33.1	28.3	43.6
505	HTH_DNA_binding_protein	83141..84781	1641	0.693	36.2	47.2	33.1	28.3	43.6
506	HTH_DNA_binding_protein	77016..78656	1641	0.689	36.1	47.3	32.7	28.3	45
507	hypothetical_protein	8287..8433	147	0.553	38.8	42.9	24.5	49	43.5
508	hypothetical_protein	11494..11640	147	0.553	38.8	42.9	24.5	49	43.5

509	hypothetical_protein	8287..8433	147	0.553	38.8	42.9	24.5	49	43.5
510	hypothetical_protein	34193..34405	213	0.591	47.4	46.5	49.3	46.5	42.9
511	hypothetical_protein	complement(127356..127571)	216	0.638	37.5	43.1	23.6	45.8	60.7
512	hypothetical_protein	complement(127134..127331)	198	0.578	46	53	39.4	45.5	45.4
513	hypothetical_protein	26520..26681	162	0.528	40.1	33.3	42.6	44.4	58.1
514	hypothetical_protein	5141..5338	198	0.596	45.5	53	39.4	43.9	61
515	hypothetical_protein	10995..11192	198	0.596	45.5	53	39.4	43.9	61
516	hypothetical_protein	complement(19219..19446)	228	0.556	39.5	46.1	28.9	43.4	40.9
517	hypothetical_protein	complement(19219..19446)	228	0.556	39.5	46.1	28.9	43.4	40.9
518	hypothetical_protein	complement(22553..22780)	228	0.574	39.9	47.4	30.3	42.1	44.4
519	hypothetical_protein	complement(13237..13407)	171	0.588	37.4	43.9	26.3	42.1	58.3
520	hypothetical_protein	complement(62260..62475)	216	0.626	39.4	41.7	34.7	41.7	48.2
521	hypothetical_protein	complement(55495..55749)	255	0.653	41.2	40	42.4	41.2	53
522	hypothetical_protein	7788..7985	198	0.603	43.9	53	37.9	40.9	48.7
523	hypothetical_protein	7788..7985	198	0.603	43.9	53	37.9	40.9	48.7
524	hypothetical_protein	complement(27026..27253)	228	0.599	37.7	36.8	35.5	40.8	61
525	hypothetical_protein	115718..115945	228	0.596	38.2	44.7	28.9	40.8	46.2
526	hypothetical_protein	10706..10978	273	0.629	38.5	44	30.8	40.7	39.4
527	hypothetical_protein	complement(58907..59083)	177	0.622	43.5	62.7	27.1	40.7	59.7
528	hypothetical_protein	complement(26866..26991)	126	0.622	48.4	50	54.8	40.5	32.9
529	hypothetical_protein	complement(60404..60514)	111	0.533	34.2	40.5	21.6	40.5	51.6
530	hypothetical_protein	complement(132304..132570)	267	0.593	39	42.7	33.7	40.4	61
531	hypothetical_protein	complement(132304..132570)	267	0.593	39	42.7	33.7	40.4	61
532	hypothetical_protein	complement(126196..126600)	405	0.615	40.7	51.1	31.1	40	47.5
533	hypothetical_protein	complement(17998..18117)	120	0.601	35.8	47.5	20	40	52.7
534	hypothetical_protein	complement(17998..18117)	120	0.601	35.8	47.5	20	40	52.7
535	hypothetical_protein	complement(19924..20232)	309	0.624	38.5	46.6	29.1	39.8	54.4
536	hypothetical_protein	complement(24038..24460)	423	0.595	36.2	41.8	27	39.7	41.8
537	hypothetical_protein	complement(1965..2153)	189	0.635	34.9	39.7	25.4	39.7	48.2
538	hypothetical_protein	114038..114460	423	0.596	35.7	43.3	24.1	39.7	48.5
539	hypothetical_protein	complement(20704..21126)	423	0.596	35.7	43.3	24.1	39.7	48.5
540	hypothetical_protein	complement(20704..21126)	423	0.596	35.7	43.3	24.1	39.7	48.5
541	hypothetical_protein	complement(15079..15306)	228	0.604	37.7	44.7	28.9	39.5	46.2
542	hypothetical_protein	complement(436..717)	282	0.597	37.9	41.5	33	39.4	61
543	hypothetical_protein	complement(128380..128592)	213	0.61	36.2	40.8	28.2	39.4	42.9
544	hypothetical_protein	115343..115549	207	0.642	39.1	42	36.2	39.1	46.7
545	hypothetical_protein	complement(60177..60422)	246	0.655	41.1	50	34.1	39	49.1
546	hypothetical_protein	complement(16564..16986)	423	0.6	35.7	43.3	24.8	39	49.5
547	hypothetical_protein	51273..51473	201	0.683	35.8	43.3	25.4	38.8	44.4
548	hypothetical_protein	complement(122150..122335)	186	0.655	36.6	45.2	25.8	38.7	57.9
549	hypothetical_protein	134934..135143	210	0.658	37.1	48.6	24.3	38.6	29
550	hypothetical_protein	134934..135143	210	0.658	37.1	48.6	24.3	38.6	29
551	hypothetical_protein	4547..4819	273	0.632	38.5	46.2	30.8	38.5	45.7
552	hypothetical_protein	7499..7771	273	0.635	37.4	42.9	30.8	38.5	35.5
553	hypothetical_protein	7499..7771	273	0.635	37.4	42.9	30.8	38.5	35.5
554	hypothetical_protein	complement(124880..125239)	360	0.618	37.5	50.8	23.3	38.3	41.6
555	hypothetical_protein	128041..128331	291	0.608	35.7	40.2	28.9	38.1	45.9
556	hypothetical_protein	1488..1637	150	0.732	38	44	32	38	44.5
557	hypothetical_protein	complement(120263..120436)	174	0.599	41.4	46.6	39.7	37.9	35.6
558	hypothetical_protein	complement(16590..16898)	309	0.629	38.8	46.6	32	37.9	49.4
559	hypothetical_protein	complement(16590..16898)	309	0.629	38.8	46.6	32	37.9	49.4
560	hypothetical_protein	13391..13651	261	0.652	37.5	44.8	29.9	37.9	60.4
561	hypothetical_protein	16725..16985	261	0.652	37.5	44.8	29.9	37.9	60.4
562	hypothetical_protein	13391..13651	261	0.652	37.5	44.8	29.9	37.9	60.4
563	hypothetical_protein	113173..113649	477	0.614	38.2	52.8	23.9	37.7	51
564	hypothetical_protein	complement(56222..56779)	558	0.658	39.2	47.3	32.8	37.6	46.6
565	hypothetical_protein	complement(128806..129087)	282	0.637	37.9	43.6	33	37.2	58.8
566	hypothetical_protein	complement(22923..23108)	186	0.66	36	29	41.9	37.1	53
567	hypothetical_protein	complement(22923..23108)	186	0.66	36	29	41.9	37.1	53
568	hypothetical_protein	134929..135219	291	0.634	34.7	38.1	28.9	37.1	45.4
569	hypothetical_protein	2696..2905	210	0.656	36.7	48.6	24.3	37.1	30.9
570	hypothetical_protein	12827..13012	186	0.654	36	46.8	24.2	37.1	60
571	hypothetical_protein	16161..16346	186	0.654	36	46.8	24.2	37.1	60
572	hypothetical_protein	12827..13012	186	0.654	36	46.8	24.2	37.1	60
573	hypothetical_protein	9457..9642	186	0.605	31.2	32.3	24.2	37.1	44.8
574	hypothetical_protein	complement(25843..26262)	420	0.655	39	37.1	43.6	36.4	54
575	hypothetical_protein	112238..112657	420	0.649	39	37.1	43.6	36.4	56.8
576	hypothetical_protein	52343..52648	306	0.666	37.3	47.1	28.4	36.3	42.2
577	hypothetical_protein	118943..119149	207	0.643	37.7	42	34.8	36.2	41.5
578	hypothetical_protein	122237..122443	207	0.643	37.7	42	34.8	36.2	41.5
579	hypothetical_protein	118943..119149	207	0.643	37.7	42	34.8	36.2	41.5
580	hypothetical_protein	131610..131900	291	0.645	34.4	38.1	28.9	36.1	39.9
581	hypothetical_protein	131610..131900	291	0.645	34.4	38.1	28.9	36.1	39.9
582	hypothetical_protein	complement(32324..32755)	432	0.625	34.5	38.9	28.5	36.1	41
583	hypothetical_protein	9166..9456	291	0.638	39.5	56.7	25.8	36.1	53.4
584	hypothetical_protein	5968..6225	258	0.692	36	47.7	24.4	36	58.6
585	hypothetical_protein	5968..6225	258	0.692	36	47.7	24.4	36	58.6
586	hypothetical_protein	118266..118574	309	0.651	36.6	43.7	30.1	35.9	53.8
587	hypothetical_protein	complement(18367..18786)	420	0.628	38.6	38.6	41.4	35.7	48.6
588	hypothetical_protein	8926..9144	219	0.625	42	43.8	46.6	35.6	42
589	hypothetical_protein	8926..9144	219	0.625	42	43.8	46.6	35.6	42
590	hypothetical_protein	complement(121511..121771)	261	0.666	36.4	42.5	31	35.6	51.2
591	hypothetical_protein	11373..11735	363	0.592	36.4	49.6	24	35.5	58.4
592	hypothetical_protein	complement(127654..127926)	273	0.653	37	45.1	30.8	35.2	37.5
593	hypothetical_protein	complement(13450..13977)	528	0.631	35.6	44.3	27.3	35.2	46.5
594	hypothetical_protein	117047..117166	120	0.602	31.7	45	15	35	52.9
595	hypothetical_protein	complement(21332..21451)	120	0.602	31.7	45	15	35	52.9
596	hypothetical_protein	6893..7399	507	0.628	37.3	48.5	28.4	34.9	52
597	hypothetical_protein	118593..118979	387	0.642	37.2	48.8	27.9	34.9	44.6
598	hypothetical_protein	9176..9433	258	0.686	36.4	51.2	23.3	34.9	44.4
599	hypothetical_protein	complement(29623..29820)	198	0.631	35.4	43.9	27.3	34.8	39.7
600	hypothetical_protein	complement(18455..18679)	225	0.676	33.3	38.7	26.7	34.7	45.1
601	hypothetical_protein	complement(18455..18679)	225	0.676	33.3	38.7	26.7	34.7	45.1
602	hypothetical_protein	14726..15088	363	0.595	36.1	50.4	23.1	34.7	57.7
603	hypothetical_protein	18060..18422	363	0.595	36.1	50.4	23.1	34.7	57.7
604	hypothetical_protein	14726..15088	363	0.595	36.1	50.4	23.1	34.7	57.7
605	hypothetical_protein	125591..126421	831	0.602	35.7	40.1	32.9	34.3	43.5
606	hypothetical_protein	12581..12817	237	0.663	31.6	38	22.8	34.2	41
607	hypothetical_protein	complement(64333..64578)	246	0.645	37	43.9	32.9	34.1	58.8
608	hypothetical_protein	115299..115685	387	0.652	37	49.6	27.1	34.1	41.9
609	hypothetical_protein	115299..115685	387	0.652	37	49.6	27.1	34.1	41.9
610	hypothetical_protein	10017..10298	282	0.698	34.8	40.4	29.8	34	45.2
611	hypothetical_protein	109878..110354	477	0.643	36.7	51.6	24.5	34	45.3
612	hypothetical_protein	109878..110354	477	0.643	36.7	51.6	24.5	34	45.3
613	hypothetical_protein	54365..54664	300	0.733	35.7	49	24	34	39.9
614	hypothetical_protein	106258..106734	477	0.641	36.5	51.6	23.9	34	48.7
615	hypothetical_protein	complement(56047..56232)	186	0.699	37.6	41.9	37.1	33.9	36.6
616	hypothetical_protein	9453..9638	186	0.674	34.9	45.2	25.8	33.9	53
617	hypothetical_protein	6249..6434	186	0.641	29	29	24.2	33.9	40.7
618	hypothetical_protein	6249..6434	186	0.641	29	29	24.2	33.9	40.7
619	hypothetical_protein	9146..9367	222	0.619	40.1	56.8	29.7	33.8	38
620	hypothetical_protein	12353..12574	222	0.619	40.1	56.8	29.7	33.8	38
621	hypothetical_protein	9146..9367	222	0.619	40.1	56.8	29.7	33.8	38
622	hypothetical_protein	complement(131015..131236)	222	0.727	36	45.9	28.4	33.8	35.4
623	hypothetical_protein	6874..7095	222	0.727	36	45.9	28.4	33.8	35.4
624	hypothetical_protein	946..1167	222	0.724	35.6	45.9	27	33.8	36.3
625	hypothetical_protein	complement(12924..13190)	267	0.636	36.3	43.8	31.5	33.7	48.3
626	hypothetical_protein	complement(19611..19877)	267	0.64	36.7	46.1	30.3	33.7	43.3
627	hypothetical_protein	9480..9746	267	0.651	37.8	53.9	25.8	33.7	35.2
628	hypothetical_protein	9480..9746	267	0.651	37.8	53.9	25.8	33.7	35.2
629	hypothetical_protein	12814..13080	267	0.654	37.5	52.8	25.8	33.7	33.2

630	hypothetical_protein	complement(129198..129455)	258	0.693	35.7	48.8	24.4	33.7	56.6
631	hypothetical_protein	complement(22014..22556)	543	0.628	36.1	50.3	24.3	33.7	44.4
632	hypothetical_protein	complement(22507..22926)	420	0.669	37.6	37.9	41.4	33.6	51.7
633	hypothetical_protein	complement(22507..22926)	420	0.669	37.6	37.9	41.4	33.6	51.7
634	hypothetical_protein	117827..118273	447	0.696	38.3	46.3	34.9	33.6	42.1
635	hypothetical_protein	complement(119717..120073)	357	0.672	34.7	38.7	31.9	33.6	35
636	hypothetical_protein	118829..119533	705	0.607	35.3	42.1	30.2	33.6	43.6
637	hypothetical_protein	complement(20188..20544)	357	0.674	36.4	46.2	29.4	33.6	42.4
638	hypothetical_protein	121879..122325	447	0.64	36	45.6	28.9	33.6	46.6
639	hypothetical_protein	121879..122325	447	0.64	36	45.6	28.9	33.6	46.6
640	hypothetical_protein	125161..125616	456	0.644	36.2	46.7	28.3	33.6	49
641	hypothetical_protein	complement(15591..16136)	546	0.633	41	48.4	41.2	33.5	44.9
642	hypothetical_protein	49913..50146	234	0.654	35.9	41	33.3	33.3	44.7
643	hypothetical_protein	18422..18781	360	0.674	34.7	39.2	31.7	33.3	34.9
644	hypothetical_protein	98241..98672	432	0.636	34	38.2	30.6	33.3	42.6
645	hypothetical_protein	11735..12094	360	0.659	33.6	37.5	30	33.3	32.3
646	hypothetical_protein	complement(125334..125639)	306	0.66	35.9	46.1	28.4	33.3	39.7
647	hypothetical_protein	complement(18803..19189)	387	0.66	36.7	49.6	27.1	33.3	42.6
648	hypothetical_protein	111699..112085	387	0.659	36.7	49.6	27.1	33.3	43.1
649	hypothetical_protein	2912..3055	144	0.732	36.1	47.9	27.1	33.3	46.1
650	hypothetical_protein	2912..3055	144	0.732	36.1	47.9	27.1	33.3	46.1
651	hypothetical_protein	complement(38951..39184)	234	0.675	36.3	48.7	26.9	33.3	47.6
652	hypothetical_protein	complement(34778..35011)	234	0.675	36.3	48.7	26.9	33.3	47.6
653	hypothetical_protein	complement(42282..42515)	234	0.675	36.3	48.7	26.9	33.3	47.6
654	hypothetical_protein	complement(38951..39184)	234	0.675	36.3	48.7	26.9	33.3	47.6
655	hypothetical_protein	2573..2779	207	0.724	36.7	52.2	24.6	33.3	61
656	hypothetical_protein	2573..2779	207	0.724	36.7	52.2	24.6	33.3	61
657	hypothetical_protein	115942..116484	543	0.624	36.5	52.5	23.8	33.1	44
658	hypothetical_protein	118624..118887	264	0.637	36	43.2	31.8	33	48.3
659	hypothetical_protein	complement(63363..63572)	210	0.636	35.2	41.4	31.4	32.9	44.2
660	hypothetical_protein	123694..124422	729	0.645	35.8	44	30.5	32.9	44
661	hypothetical_protein	complement(65049..65267)	219	0.639	33.8	38.4	30.1	32.9	47.6
662	hypothetical_protein	complement(12163..12609)	447	0.656	36	46.3	28.9	32.9	50.6
663	hypothetical_protein	complement(27899..28117)	219	0.643	36.5	47.9	28.8	32.9	45.6
664	hypothetical_protein	complement(29049..29837)	789	0.635	35.2	43	30	32.7	51.5
665	hypothetical_protein	118279..118728	450	0.658	36	46.7	28.7	32.7	50.6
666	hypothetical_protein	complement(24246..24722)	477	0.649	36.1	51.6	23.9	32.7	45.6
667	hypothetical_protein	complement(36505..36936)	432	0.64	33.8	38.9	29.9	32.6	43.3
668	hypothetical_protein	complement(39839..40270)	432	0.64	33.8	38.9	29.9	32.6	43.3
669	hypothetical_protein	complement(36505..36936)	432	0.64	33.8	38.9	29.9	32.6	43.3
670	hypothetical_protein	4270..4812	543	0.63	35.7	45.3	29.3	32.6	47
671	hypothetical_protein	4270..4812	543	0.63	35.7	45.3	29.3	32.6	47
672	hypothetical_protein	complement(25784..26437)	654	0.698	35.9	49.1	26.1	32.6	49.9
673	hypothetical_protein	complement(36391..36528)	138	0.645	34.1	45.7	23.9	32.6	45.8
674	hypothetical_protein	120460..121344	885	0.645	36.5	42.7	34.2	32.5	45.1
675	hypothetical_protein	120460..121344	885	0.645	36.5	42.7	34.2	32.5	45.1
676	hypothetical_protein	15088..15447	360	0.685	34.2	39.2	30.8	32.5	34.6
677	hypothetical_protein	15088..15447	360	0.685	34.2	39.2	30.8	32.5	34.6
678	hypothetical_protein	44731..45210	480	0.654	34.4	43.8	26.9	32.5	49.2
679	hypothetical_protein	complement(12615..13049)	435	0.701	37.7	46.2	34.5	32.4	41.4
680	hypothetical_protein	complement(125752..125973)	222	0.624	39.6	56.8	29.7	32.4	37.6
681	hypothetical_protein	6306..6527	222	0.624	39.6	56.8	29.7	32.4	37.6
682	hypothetical_protein	8723..9148	426	0.664	35.4	46.5	27.5	32.4	44.4
683	hypothetical_protein	complement(123107..123532)	426	0.666	35.2	45.8	27.5	32.4	43.4
684	hypothetical_protein	122426..123130	705	0.612	34.9	42.1	30.2	32.3	42.6
685	hypothetical_protein	122426..123130	705	0.612	34.9	42.1	30.2	32.3	42.6
686	hypothetical_protein	121436..121873	438	0.702	37.9	47.3	34.2	32.2	44.1
687	hypothetical_protein	121436..121873	438	0.702	37.9	47.3	34.2	32.2	44.1
688	hypothetical_protein	complement(13144..14028)	885	0.646	36.3	43.1	33.6	32.2	44.6
689	hypothetical_protein	116860..117744	885	0.643	36.3	43.1	33.6	32.2	45.1
690	hypothetical_protein	108217..108720	504	0.669	36.9	42.3	36.3	32.1	46.4
691	hypothetical_protein	complement(8976..9443)	468	0.646	39.1	53.2	32.1	32.1	42.2
692	hypothetical_protein	96002..96235	234	0.692	35.9	48.7	26.9	32.1	46.1
693	hypothetical_protein	complement(325..474)	150	0.778	36.7	44	34	32	39.7
694	hypothetical_protein	129852..130001	150	0.778	36.7	44	34	32	39.7
695	hypothetical_protein	10233..10739	507	0.648	37.5	50.9	29.6	32	48.5
696	hypothetical_protein	13567..14073	507	0.648	37.5	50.9	29.6	32	48.5
697	hypothetical_protein	10233..10739	507	0.648	37.5	50.9	29.6	32	48.5
698	hypothetical_protein	5644..6168	525	0.707	35.8	47.4	28	32	46.7
699	hypothetical_protein	complement(14315..14539)	225	0.696	34.2	42.7	28	32	50.8
700	hypothetical_protein	complement(122799..123089)	291	0.667	37.8	55.7	25.8	32	49.9
701	hypothetical_protein	complement(18680..19222)	543	0.622	35.7	50.3	24.9	32	42.9
702	hypothetical_protein	complement(18680..19222)	543	0.622	35.7	50.3	24.9	32	42.9
703	hypothetical_protein	complement(24328..24684)	357	0.675	36.1	47.1	29.4	31.9	39.2
704	hypothetical_protein	complement(27664..28020)	357	0.675	36.1	47.1	29.4	31.9	39.2
705	hypothetical_protein	complement(24328..24684)	357	0.675	36.1	47.1	29.4	31.9	39.2
706	hypothetical_protein	112847..113479	633	0.631	37.6	45	36	31.8	45.9
707	hypothetical_protein	11046..11243	198	0.675	36.4	47	30.3	31.8	39.9
708	hypothetical_protein	complement(19449..20702)	1254	0.675	35.7	45.7	29.7	31.8	45.6
709	hypothetical_protein	complement(19449..20702)	1254	0.675	35.7	45.7	29.7	31.8	45.6
710	hypothetical_protein	complement(60719..60916)	198	0.72	33.8	50	19.7	31.8	51.2
711	hypothetical_protein	53684..53911	228	0.654	36.8	42.1	36.8	31.6	37.1
712	hypothetical_protein	complement(29190..29597)	408	0.615	33.1	33.1	34.6	31.6	42.9
713	hypothetical_protein	121448..121903	456	0.654	38.6	53.3	30.9	31.6	42.7
714	hypothetical_protein	42213..42479	267	0.699	41.6	50.6	42.7	31.5	36.6
715	hypothetical_protein	124718..125164	447	0.708	37.6	46.3	34.9	31.5	39.7
716	hypothetical_protein	complement(90134..90514)	381	0.661	35.2	42.5	31.5	31.5	39.8
717	hypothetical_protein	complement(16277..16543)	267	0.651	36	46.1	30.3	31.5	42.2
718	hypothetical_protein	complement(16277..16543)	267	0.651	36	46.1	30.3	31.5	42.2
719	hypothetical_protein	102908..103126	219	0.652	35.6	46.6	28.8	31.5	48.7
720	hypothetical_protein	complement(32040..32258)	219	0.652	35.6	46.6	28.8	31.5	48.7
721	hypothetical_protein	complement(35374..35592)	219	0.652	35.6	46.6	28.8	31.5	48.7
722	hypothetical_protein	complement(32040..32258)	219	0.652	35.6	46.6	28.8	31.5	48.7
723	hypothetical_protein	complement(64086..64343)	258	0.687	36	36	40.7	31.4	43.2
724	hypothetical_protein	118352..118897	546	0.65	39.9	47.8	40.7	31.3	41.2
725	hypothetical_protein	121646..122191	546	0.65	39.9	47.8	40.7	31.3	41.2
726	hypothetical_protein	118352..118897	546	0.65	39.9	47.8	40.7	31.3	41.2
727	hypothetical_protein	complement(29931..32390)	2460	0.683	36.4	45.2	32.6	31.3	45.1
728	hypothetical_protein	38724..39416	693	0.683	42.1	58.9	36.4	31.2	44
729	hypothetical_protein	82483..82704	222	0.597	34.2	47.3	24.3	31.1	47.4
730	hypothetical_protein	15431..15856	426	0.68	34.7	46.5	26.8	31	41.8
731	hypothetical_protein	complement(58036..58200)	165	0.646	33.9	36.4	34.5	30.9	36.7
732	hypothetical_protein	complement(7781..8353)	573	0.649	34.2	39.8	31.9	30.9	47.3
733	hypothetical_protein	complement(14540..15082)	543	0.639	34.4	49.2	23.2	30.9	50
734	hypothetical_protein	complement(64813..65046)	234	0.7	38.5	47.4	37.2	30.8	59.2
735	hypothetical_protein	complement(55829..56023)	195	0.634	36.9	43.1	36.9	30.8	45.6
736	hypothetical_protein	complement(57320..57952)	633	0.684	34.9	46.4	27.5	30.8	47.6
737	hypothetical_protein	53908..54267	360	0.656	34.2	44.2	27.5	30.8	40.4
738	hypothetical_protein	complement(58211..58366)	156	0.714	30.1	32.7	26.9	30.8	41.1
739	hypothetical_protein	complement(557..916)	360	0.784	31.9	40.8	24.2	30.8	41.6
740	hypothetical_protein	129410..129769	360	0.784	31.9	40.8	24.2	30.8	41.6
741	hypothetical_protein	132911..133270	360	0.769	31.9	40.8	24.2	30.8	42.3
742	hypothetical_protein	1043..1402	360	0.769	31.9	40.8	24.2	30.8	42.3
743	hypothetical_protein	132911..133270	360	0.769	31.9	40.8	24.2	30.8	42.3
744	hypothetical_protein	6358..6699	342	0.741	34.2	43.9	28.1	30.7	36.5
745	hypothetical_protein	131449..131712	264	0.794	33.3	42	27.3	30.7	42.8
746	hypothetical_protein	3014..3277	264	0.789	32.6	39.8	27.3	30.7	46.2
747	hypothetical_protein	complement(21789..22013)	225	0.713	32.4	40	26.7	30.7	49.9
748	hypothetical_protein	79412..80704	1293	0.676	38.7	43.6	42	30.6	48.2
749	hypothetical_protein	79412..80704	1293	0.676	38.7	43.6	42	30.6	48.2
750	hypothetical_protein	50980..51273	294	0.738	38.8	48	37.8	30.6	55.6

751	hypothetical_protein	126114..126692	579	0.658	36.8	47.7	32.1	30.6	45.3
752	hypothetical_protein	126114..126692	579	0.658	36.8	47.7	32.1	30.6	45.3
753	hypothetical_protein	129405..129983	579	0.658	36.6	47.2	32.1	30.6	43.9
754	hypothetical_protein	5714..5968	255	0.641	33.7	42.4	28.2	30.6	41.3
755	hypothetical_protein	5714..5968	255	0.641	33.7	42.4	28.2	30.6	41.3
756	hypothetical_protein	8922..9176	255	0.641	33.3	41.2	28.2	30.6	41
757	hypothetical_protein	52707..53543	837	0.697	39.9	54.5	34.8	30.5	53.6
758	hypothetical_protein	130594..131322	729	0.665	35	44	30.5	30.5	43.3
759	hypothetical_protein	82707..83999	1293	0.675	39.1	44.5	42.2	30.4	48.6
760	hypothetical_protein	50689..50964	276	0.721	34.8	41.3	32.6	30.4	45.6
761	hypothetical_protein	3816..4091	276	0.683	34.8	43.5	30.4	30.4	50.9
762	hypothetical_protein	3863..4219	357	0.691	38.4	49.6	35.3	30.3	44.3
763	hypothetical_protein	110480..110836	357	0.699	35.3	46.2	29.4	30.3	41.8
764	hypothetical_protein	complement(58363..58620)	258	0.689	38	53.5	30.2	30.2	43.2
765	hypothetical_protein	6598..6945	348	0.688	34.8	44	30.2	30.2	34.1
766	hypothetical_protein	complement(11358..12062)	705	0.624	33.8	41.7	29.4	30.2	38.7
767	hypothetical_protein	3010..3267	258	0.719	34.9	53.5	20.9	30.2	47.5
768	hypothetical_protein	complement(125975..126193)	219	0.671	38.8	42.5	43.8	30.1	39.3
769	hypothetical_protein	6086..6304	219	0.671	38.8	42.5	43.8	30.1	39.3
770	hypothetical_protein	12133..12351	219	0.671	38.8	42.5	43.8	30.1	39.3
771	hypothetical_protein	4837..5115	279	0.653	38.7	45.2	40.9	30.1	41.1
772	hypothetical_protein	4837..5115	279	0.653	38.7	45.2	40.9	30.1	41.1
773	hypothetical_protein	98775..99962	1188	0.649	37.4	45.7	36.4	30.1	43.6
774	hypothetical_protein	98775..99962	1188	0.649	37.4	45.7	36.4	30.1	43.6
775	hypothetical_protein	complement(35201..36388)	1188	0.649	37.3	45.7	36.1	30.1	43.7
776	hypothetical_protein	10750..11934	1185	0.685	35.6	42.3	34.4	30.1	49.3
777	hypothetical_protein	10750..11934	1185	0.685	35.6	42.3	34.4	30.1	49.3
778	hypothetical_protein	14084..15268	1185	0.678	35.5	42.5	33.9	30.1	49.9
779	hypothetical_protein	complement(7206..7784)	579	0.658	36.6	47.7	32.1	30.1	46
780	hypothetical_protein	125045..125512	468	0.656	38.7	55.1	30.8	30.1	45.7
781	hypothetical_protein	125045..125512	468	0.656	38.7	55.1	30.8	30.1	45.7
782	hypothetical_protein	complement(15309..16562)	1254	0.68	35.1	45.2	29.9	30.1	47.6
783	hypothetical_protein	72048..72605	558	0.652	33	40.3	28.5	30.1	41.4
784	hypothetical_protein	122482..123201	720	0.647	35.1	42.5	32.9	30	45.5
785	hypothetical_protein	133354..133503	150	0.791	35.3	44	32	30	40.5
786	hypothetical_protein	133354..133503	150	0.791	35.3	44	32	30	40.5
787	hypothetical_protein	127303..128031	729	0.67	34.8	44	30.5	30	43.2
788	hypothetical_protein	127303..128031	729	0.67	34.8	44	30.5	30	43.2
789	hypothetical_protein	complement(5867..6595)	729	0.67	34.7	43.6	30.5	30	42.3
790	hypothetical_protein	complement(90504..91142)	639	0.657	37.1	51.2	30	30	49.3
791	hypothetical_protein	44044..44682	639	0.662	36.9	50.7	30	30	51.3
792	hypothetical_protein	47375..48013	639	0.662	36.9	50.7	30	30	51.3
793	hypothetical_protein	44044..44682	639	0.662	36.9	50.7	30	30	51.3
794	hypothetical_protein	complement(125636..125755)	120	0.707	35	45	30	30	36.5
795	hypothetical_protein	complement(123685..124869)	1185	0.685	35.9	42.5	35.2	29.9	49.3
796	hypothetical_protein	44672..45052	381	0.667	34.6	42.5	31.5	29.9	39.8
797	hypothetical_protein	48003..48383	381	0.667	34.6	42.5	31.5	29.9	39.8
798	hypothetical_protein	44672..45052	381	0.667	34.6	42.5	31.5	29.9	39.8
799	hypothetical_protein	complement(21448..21729)	282	0.654	36.2	43.6	35.1	29.8	35.2
800	hypothetical_protein	3258..3599	342	0.742	34.5	45.6	28.1	29.8	37.1
801	hypothetical_protein	3258..3599	342	0.742	34.5	45.6	28.1	29.8	37.1
802	hypothetical_protein	75207..76499	1293	0.677	38.7	43.6	42.9	29.7	48.8
803	hypothetical_protein	complement(54488..55780)	1293	0.677	38.5	43.4	42.5	29.7	48.5
804	hypothetical_protein	114752..115297	546	0.668	39.9	49.5	40.7	29.7	40.7
805	hypothetical_protein	7410..8570	1161	0.687	35.1	41.9	33.9	29.7	46.8
806	hypothetical_protein	119200..119907	708	0.649	34.9	41.9	33.1	29.7	45.4
807	hypothetical_protein	119200..119907	708	0.649	34.9	41.9	33.1	29.7	45.4
808	hypothetical_protein	complement(22783..24036)	1254	0.686	35.2	45.5	30.6	29.7	47.3
809	hypothetical_protein	complement(22114..22305)	192	0.685	30.7	32.8	29.7	29.7	47
810	hypothetical_protein	79188..79409	222	0.609	34.7	47.3	27	29.7	48.1
811	hypothetical_protein	79188..79409	222	0.609	34.7	47.3	27	29.7	48.1
812	hypothetical_protein	105459..105701	243	0.664	33.7	42	29.6	29.6	40.1
813	hypothetical_protein	complement(63776..64039)	264	0.701	33	31.8	37.5	29.5	55.2
814	hypothetical_protein	46806..46988	183	0.714	35.5	42.6	34.4	29.5	40.9
815	hypothetical_protein	complement(8392..8970)	579	0.681	37.8	51.3	32.6	29.5	43.6
816	hypothetical_protein	128336..128803	468	0.658	38.5	54.5	31.4	29.5	44.6
817	hypothetical_protein	complement(4194..5270)	1077	0.669	37.8	52.6	31.2	29.5	44.9
818	hypothetical_protein	81..344	264	0.788	33.3	43.2	27.3	29.5	45.7
819	hypothetical_protein	81..344	264	0.788	33.3	43.2	27.3	29.5	45.7
820	hypothetical_protein	complement(17051..17539)	489	0.686	36.4	43.6	36.2	29.4	51.5
821	hypothetical_protein	83962..84441	480	0.656	36.5	50	30	29.4	44.1
822	hypothetical_protein	9739..10230	492	0.643	36	43.9	34.8	29.3	45.6
823	hypothetical_protein	13073..13564	492	0.643	36	43.9	34.8	29.3	45.6
824	hypothetical_protein	9739..10230	492	0.643	36	43.9	34.8	29.3	45.6
825	hypothetical_protein	9638..9985	348	0.704	35.1	42.2	33.6	29.3	48.1
826	hypothetical_protein	121936..122508	573	0.667	33.7	40.8	30.9	29.3	48.4
827	hypothetical_protein	3361..3636	276	0.724	30.4	32.6	29.3	29.3	47.4
828	hypothetical_protein	6768..7043	276	0.686	34.4	45.7	28.3	29.3	48.5
829	hypothetical_protein	6768..7043	276	0.686	34.4	45.7	28.3	29.3	48.5
830	hypothetical_protein	119918..120400	483	0.668	36.9	41	40.4	29.2	39.5
831	hypothetical_protein	123212..123694	483	0.668	36.9	41	40.4	29.2	39.5
832	hypothetical_protein	119918..120400	483	0.668	36.9	41	40.4	29.2	39.5
833	hypothetical_protein	123754..124638	885	0.677	34.7	41.7	33.2	29.2	41.2
834	hypothetical_protein	complement(60918..61277)	360	0.705	35.8	45.8	32.5	29.2	43.2
835	hypothetical_protein	126722..127255	534	0.675	36	47.2	31.5	29.2	48
836	hypothetical_protein	complement(56794..56937)	144	0.716	37.5	54.2	29.2	29.2	38.9
837	hypothetical_protein	127270..128028	759	0.632	35.4	47.8	29.2	29.2	39.8
838	hypothetical_protein	40499..40879	381	0.674	34.6	42.5	32.3	29.1	42.8
839	hypothetical_protein	114462..115715	1254	0.695	35.1	46.4	29.9	28.9	45
840	hypothetical_protein	complement(39311..39580)	270	0.662	33.3	42.2	28.9	28.9	42.7
841	hypothetical_protein	complement(35138..35407)	270	0.662	33.3	42.2	28.9	28.9	42.7
842	hypothetical_protein	complement(42642..42911)	270	0.662	33.3	42.2	28.9	28.9	42.7
843	hypothetical_protein	complement(39311..39580)	270	0.662	33.3	42.2	28.9	28.9	42.7
844	hypothetical_protein	complement(131411..131752)	342	0.749	32.7	42.1	27.2	28.9	38.2
845	hypothetical_protein	12097..12522	426	0.702	33.6	45.1	26.8	28.9	39.7
846	hypothetical_protein	12097..12522	426	0.702	33.6	45.1	26.8	28.9	39.7
847	hypothetical_protein	12540..12830	291	0.703	37.5	57.7	25.8	28.9	42.1
848	hypothetical_protein	12540..12830	291	0.703	37.5	57.7	25.8	28.9	42.1
849	hypothetical_protein	15874..16164	291	0.696	37.1	56.7	25.8	28.9	40.7
850	hypothetical_protein	complement(14581..15288)	708	0.652	34.6	41.9	33.1	28.8	47.1
851	hypothetical_protein	123076..123408	333	0.712	31.8	34.2	32.4	28.8	36.8
852	hypothetical_protein	101207..101404	198	0.681	34.3	45.5	28.8	28.8	44.2
853	hypothetical_protein	complement(33762..33959)	198	0.681	34.3	45.5	28.8	28.8	44.2
854	hypothetical_protein	complement(37096..37293)	198	0.681	34.3	45.5	28.8	28.8	44.2
855	hypothetical_protein	complement(33762..33959)	198	0.681	34.3	45.5	28.8	28.8	44.2
856	hypothetical_protein	116769..117050	282	0.667	35.8	44.7	34	28.7	36.2
857	hypothetical_protein	complement(18114..18395)	282	0.667	35.8	44.7	34	28.7	36.2
858	hypothetical_protein	complement(18114..18395)	282	0.667	35.8	44.7	34	28.7	36.2
859	hypothetical_protein	complement(33329..33736)	408	0.636	31.4	33.1	32.4	28.7	42.9
860	hypothetical_protein	complement(36663..37070)	408	0.636	31.4	33.1	32.4	28.7	42.9
861	hypothetical_protein	complement(33329..33736)	408	0.636	31.4	33.1	32.4	28.7	42.9
862	hypothetical_protein	120541..121386	846	0.648	34.6	43.3	31.9	28.7	46.7
863	hypothetical_protein	116321..116698	378	0.722	34.7	40.5	34.9	28.6	41.4
864	hypothetical_protein	41301..42212	912	0.702	35.7	45.1	33.6	28.6	50.2
865	hypothetical_protein	39871..40509	639	0.669	36.5	50.7	30	28.6	49.4
866	hypothetical_protein	complement(20248..20499)	252	0.728	36.5	54.8	26.2	28.6	48.5
867	hypothetical_protein	94601..95788	1188	0.657	37.3	45.2	38.1	28.5	43.7
868	hypothetical_protein	complement(60516..60716)	201	0.726	35.8	41.8	37.3	28.4	48.9
869	hypothetical_protein	37370..37918	549	0.683	37.9	51.9	33.3	28.4	45.3
870	hypothetical_protein	421..768	348	0.74	33	43.1	27.6	28.4	41.4
871	hypothetical_protein	130112..130894	783	0.708	32.4	43.7	25.3	28.4	41.8

872	hypothetical_protein	74983..75204	222	0.607	33.3	47.3	24.3	28.4	51.2
873	hypothetical_protein	1747..2529	783	0.718	32.1	43.7	24.1	28.4	45.1
874	hypothetical_protein	98796..99071	276	0.745	36.6	45.7	35.9	28.3	37.8
875	hypothetical_protein	131796..132071	276	0.748	30.1	31.5	30.4	28.3	53.8
876	hypothetical_protein	134587..134862	276	0.765	36.2	51.1	29.3	28.3	39.8
877	hypothetical_protein	134587..134862	276	0.765	36.2	51.1	29.3	28.3	39.8
878	hypothetical_protein	complement(21191..21679)	489	0.696	36	43.6	36.2	28.2	48.1
879	hypothetical_protein	complement(21191..21679)	489	0.696	36	43.6	36.2	28.2	48.1
880	hypothetical_protein	complement(24527..25015)	489	0.697	35.8	42.9	36.2	28.2	49.7
881	hypothetical_protein	complement(16144..16527)	384	0.699	37	48.4	34.4	28.1	46.1
882	hypothetical_protein	117961..118344	384	0.699	37	48.4	34.4	28.1	46.1
883	hypothetical_protein	121255..121638	384	0.699	37	48.4	34.4	28.1	46.1
884	hypothetical_protein	117961..118344	384	0.699	37	48.4	34.4	28.1	46.1
885	hypothetical_protein	133610..134143	534	0.681	36	47.2	32.6	28.1	47
886	hypothetical_protein	130291..130824	534	0.678	35.6	47.2	31.5	28.1	48.6
887	hypothetical_protein	130291..130824	534	0.678	35.6	47.2	31.5	28.1	48.6
888	hypothetical_protein	complement(26252..26443)	192	0.694	30.7	32.8	31.2	28.1	45.4
889	hypothetical_protein	complement(29588..29779)	192	0.694	30.7	32.8	31.2	28.1	45.4
890	hypothetical_protein	complement(26252..26443)	192	0.694	30.7	32.8	31.2	28.1	45.4
891	hypothetical_protein	108721..108912	192	0.695	30.2	32.8	29.7	28.1	39.1
892	hypothetical_protein	64572..65105	534	0.665	32.4	40.4	28.7	28.1	41.6
893	hypothetical_protein	115600..116307	708	0.657	34.6	42.4	33.5	28	46.4
894	hypothetical_protein	complement(58620..58898)	279	0.719	34.4	41.9	33.3	28	42.4
895	hypothetical_protein	94964..95188	225	0.637	34.7	45.3	30.7	28	47.3
896	hypothetical_protein	17065..17397	333	0.691	36.3	45.9	35.1	27.9	39.1
897	hypothetical_protein	126685..127017	333	0.719	32.1	33.3	35.1	27.9	33
898	hypothetical_protein	126685..127017	333	0.719	32.1	33.3	35.1	27.9	33
899	hypothetical_protein	129976..130308	333	0.719	31.8	33.3	34.2	27.9	32.9
900	hypothetical_protein	complement(66466..66852)	387	0.732	35.1	46.5	31	27.9	36.8
901	hypothetical_protein	106377..106580	204	0.696	30.4	35.3	27.9	27.9	51.7
902	hypothetical_protein	complement(56934..57320)	387	0.703	35.1	50.4	27.1	27.9	35.8
903	hypothetical_protein	5441..5569	129	0.659	32.6	48.8	20.9	27.9	38
904	hypothetical_protein	109412..109789	378	0.725	34.7	41.3	34.9	27.8	43.1
905	hypothetical_protein	3712..4164	453	0.738	31.8	37.7	29.8	27.8	42.7
906	hypothetical_protein	87685..88008	324	0.702	34.3	47.2	27.8	27.8	36.9
907	hypothetical_protein	58322..58939	618	0.685	34.5	36.4	39.3	27.7	45.7
908	hypothetical_protein	80736..81146	411	0.676	37.5	53.3	31.4	27.7	40.3
909	hypothetical_protein	80736..81146	411	0.676	37.5	53.3	31.4	27.7	40.3
910	hypothetical_protein	125545..126117	573	0.671	33.3	41.9	30.4	27.7	46.5
911	hypothetical_protein	125545..126117	573	0.671	33.3	41.9	30.4	27.7	46.5
912	hypothetical_protein	128836..129408	573	0.669	33.2	41.9	29.8	27.7	47
913	hypothetical_protein	7677..8024	348	0.641	33.3	44	28.4	27.6	38.2
914	hypothetical_protein	2056..2283	228	0.665	29.4	34.2	26.3	27.6	50.5
915	hypothetical_protein	2056..2283	228	0.665	29.4	34.2	26.3	27.6	50.5
916	hypothetical_protein	122505..123083	579	0.682	35.2	45.6	32.6	27.5	43.2
917	hypothetical_protein	complement(3041..3574)	534	0.682	35.6	47.2	32	27.5	41.7
918	hypothetical_protein	complement(65882..66415)	534	0.662	32.4	41	28.7	27.5	42.6
919	hypothetical_protein	complement(120073..120225)	153	0.643	28.8	41.2	17.6	27.5	30.5
920	hypothetical_protein	50875..51432	558	0.689	34.6	36.6	39.8	27.4	43.1
921	hypothetical_protein	complement(65279..65530)	252	0.682	37.3	46.4	38.1	27.4	43.5
922	hypothetical_protein	complement(59076..59306)	231	0.692	34.6	42.9	33.8	27.3	43
923	hypothetical_protein	14163..14459	297	0.682	32.3	36.4	33.3	27.3	44.9
924	hypothetical_protein	14163..14459	297	0.682	32.3	36.4	33.3	27.3	44.9
925	hypothetical_protein	127429..128274	846	0.664	33.8	42.6	31.6	27.3	46
926	hypothetical_protein	17497..17793	297	0.673	33	40.4	31.3	27.3	39
927	hypothetical_protein	130839..131597	759	0.647	34.9	48.2	29.2	27.3	37.8
928	hypothetical_protein	130839..131597	759	0.647	34.9	48.2	29.2	27.3	37.8
929	hypothetical_protein	55657..56496	840	0.679	35.2	43.9	34.6	27.1	43.7
930	hypothetical_protein	58952..59791	840	0.679	35.2	43.9	34.6	27.1	43.7
931	hypothetical_protein	55657..56496	840	0.679	35.2	43.9	34.6	27.1	43.7
932	hypothetical_protein	1749..2102	354	0.646	33.1	44.1	28	27.1	37.2
933	hypothetical_protein	113485..113973	489	0.707	35.6	43.6	36.2	27	47.7
934	hypothetical_protein	complement(54046..54456)	411	0.679	37.2	52.6	32.1	27	40
935	hypothetical_protein	124138..124983	846	0.667	33.7	42.6	31.6	27	45.5
936	hypothetical_protein	124138..124983	846	0.667	33.7	42.6	31.6	27	45.5
937	hypothetical_protein	38221..38631	411	0.687	33.3	44.5	28.5	27	48.1
938	hypothetical_protein	88001..88423	423	0.707	35.5	52.5	27	27	48.5
939	hypothetical_protein	complement(55783..56004)	222	0.616	33.3	48.6	24.3	27	50.7
940	hypothetical_protein	55087..55644	558	0.692	34.4	36.6	39.8	26.9	46
941	hypothetical_protein	55087..55644	558	0.692	34.4	36.6	39.8	26.9	46
942	hypothetical_protein	134158..134916	759	0.647	34.8	48.2	29.2	26.9	38
943	hypothetical_protein	102070..103257	1188	0.666	36.4	45.7	36.9	26.8	41.8
944	hypothetical_protein	51445..52284	840	0.684	35.1	44.3	34.3	26.8	44
945	hypothetical_protein	complement(14088..14570)	483	0.691	35.8	40.4	40.4	26.7	39
946	hypothetical_protein	116318..116800	483	0.691	35.8	40.4	40.4	26.7	39
947	hypothetical_protein	51485..51832	348	0.73	34.8	43.1	34.5	26.7	44.2
948	hypothetical_protein	13012..13359	348	0.728	33.6	40.5	33.6	26.7	44.5
949	hypothetical_protein	16346..16693	348	0.728	33.6	40.5	33.6	26.7	44.5
950	hypothetical_protein	13012..13359	348	0.728	33.6	40.5	33.6	26.7	44.5
951	hypothetical_protein	complement(43299..43523)	225	0.647	33.8	42.7	32	26.7	51.6
952	hypothetical_protein	87464..87688	225	0.638	34.2	45.3	30.7	26.7	51.3
953	hypothetical_protein	12569..12985	417	0.726	38.8	52.5	37.4	26.6	52
954	hypothetical_protein	complement(13974..14255)	282	0.689	35.1	44.7	34	26.6	35.7
955	hypothetical_protein	28126..28509	384	0.725	33.1	41.4	31.2	26.6	40.3
956	hypothetical_protein	53694..54605	912	0.709	36.5	53.3	29.6	26.6	40.8
957	hypothetical_protein	45627..46088	462	0.742	28.6	39	20.1	26.6	48.9
958	hypothetical_protein	complement(56050..59454)	3405	0.725	38.1	48.3	39.5	26.5	41.5
959	hypothetical_protein	complement(83906..84811)	906	0.709	36.8	53.3	30.5	26.5	41.3
960	hypothetical_protein	complement(28585..28788)	204	0.696	30.4	36.8	27.9	26.5	55.1
961	hypothetical_protein	complement(24447..24650)	204	0.696	30.4	36.8	27.9	26.5	55.1
962	hypothetical_protein	complement(31920..32123)	204	0.696	30.4	36.8	27.9	26.5	55.1
963	hypothetical_protein	complement(28585..28788)	204	0.696	30.4	36.8	27.9	26.5	55.1
964	hypothetical_protein	40066..40338	273	0.68	36.6	37.4	46.2	26.4	53
965	hypothetical_protein	40066..40338	273	0.68	36.6	37.4	46.2	26.4	53
966	hypothetical_protein	complement(66014..66457)	444	0.74	34.5	45.9	31.1	26.4	44.3
967	hypothetical_protein	68777..69310	534	0.683	32	41	28.7	26.4	41.4
968	hypothetical_protein	68777..69310	534	0.683	32	41	28.7	26.4	41.4
969	hypothetical_protein	133613..134395	783	0.72	32.2	44.4	25.7	26.4	41.6
970	hypothetical_protein	133613..134395	783	0.72	32.2	44.4	25.7	26.4	41.6
971	hypothetical_protein	complement(42873..42986)	114	0.721	32.5	39.5	31.6	26.3	49.8
972	hypothetical_protein	76531..76941	411	0.682	37.5	54.7	31.4	26.3	40.5
973	hypothetical_protein	133038..133607	570	0.685	34	47.4	28.4	26.3	48.2
974	hypothetical_protein	92206..92628	423	0.709	35.5	53.2	27	26.2	48.2
975	hypothetical_protein	92206..92628	423	0.709	35.5	53.2	27	26.2	48.2
976	hypothetical_protein	95501..95923	423	0.707	35.5	53.2	27	26.2	50.1
977	hypothetical_protein	50476..50796	321	0.689	31.5	42.1	26.2	26.2	43.4
978	hypothetical_protein	47145..47465	321	0.686	31.5	42.1	26.2	26.2	43.7
979	hypothetical_protein	42972..43292	321	0.686	31.5	42.1	26.2	26.2	43.7
980	hypothetical_protein	47145..47465	321	0.686	31.5	42.1	26.2	26.2	43.7
981	hypothetical_protein	complement(87709..87903)	195	0.739	32.8	52.3	20	26.2	43.7
982	hypothetical_protein	10378..10710	333	0.738	35.7	44.1	36.9	26.1	43.5
983	hypothetical_protein	complement(53912..54049)	138	0.728	33.3	41.3	32.6	26.1	41.1
984	hypothetical_protein	428..703	276	0.749	29	31.5	29.3	26.1	50.2
985	hypothetical_protein	428..703	276	0.749	29	31.5	29.3	26.1	50.2
986	hypothetical_protein	101930..102067	138	0.732	31.9	45.7	23.9	26.1	35.7
987	hypothetical_protein	43373..43591	219	0.76	33.3	39.7	34.2	26	37.8
988	hypothetical_protein	complement(121803..122150)	348	0.734	33.3	40.5	33.6	25.9	43.6
989	hypothetical_protein	complement(88132..88872)	741	0.699	35.5	47.8	32.8	25.9	45.8
990	hypothetical_protein	complement(9505..10350)	846	0.667	33	41.5	31.6	25.9	44.7
991	hypothetical_protein	95185..95508	324	0.725	33	46.3	26.9	25.9	34.7
992	hypothetical_protein	complement(129455..129709)	255	0.692	29.8	37.6	25.9	25.9	32.9

993	hypothetical_protein	779..1231	453	0.74	30.9	37.7	29.1	25.8	42.1
994	hypothetical_protein	779..1231	453	0.74	30.9	37.7	29.1	25.8	42.1
995	hypothetical_protein	129743..130288	546	0.683	34.2	48.9	28	25.8	50
996	hypothetical_protein	129743..130288	546	0.683	34.2	48.9	28	25.8	50
997	hypothetical_protein	complement(23741..24112)	372	0.712	33.9	48.4	27.4	25.8	49.2
998	hypothetical_protein	complement(78690..79529)	840	0.687	34.9	44.6	34.3	25.7	43.3
999	hypothetical_protein	1173..1616	444	0.727	33.6	43.9	31.1	25.7	42.1
1000	hypothetical_protein	complement(64571..64816)	246	0.729	37.4	45.1	41.5	25.6	46
1001	hypothetical_protein	50363..51265	903	0.717	36.2	53.2	29.9	25.6	41
1002	hypothetical_protein	50363..51265	903	0.717	36.2	53.2	29.9	25.6	41
1003	hypothetical_protein	43156..43389	234	0.794	32.5	44.9	26.9	25.6	48.8
1004	hypothetical_protein	complement(91132..91248)	117	0.681	30.8	41	25.6	25.6	30.4
1005	hypothetical_protein	43938..44054	117	0.681	30.8	41	25.6	25.6	30.4
1006	hypothetical_protein	39765..39881	117	0.681	30.8	41	25.6	25.6	30.4
1007	hypothetical_protein	47269..47385	117	0.681	30.8	41	25.6	25.6	30.4
1008	hypothetical_protein	43938..44054	117	0.681	30.8	41	25.6	25.6	30.4
1009	hypothetical_protein	complement(126903..127031)	129	0.686	31.8	48.8	20.9	25.6	34.7
1010	hypothetical_protein	11295..11423	129	0.687	31.8	48.8	20.9	25.6	36.4
1011	hypothetical_protein	8088..8216	129	0.668	31.8	48.8	20.9	25.6	37
1012	hypothetical_protein	8088..8216	129	0.668	31.8	48.8	20.9	25.6	37
1013	hypothetical_protein	45200..45634	435	0.713	35.4	44.8	35.9	25.5	39.3
1014	hypothetical_protein	43648..44436	789	0.699	30.7	39.5	27	25.5	42.7
1015	hypothetical_protein	35893..36165	273	0.687	36.3	37.4	46.2	25.3	52
1016	hypothetical_protein	43397..43669	273	0.687	36.3	37.4	46.2	25.3	52
1017	hypothetical_protein	47208..47717	510	0.696	32.2	37.6	33.5	25.3	40.7
1018	hypothetical_protein	114361..114744	384	0.716	35.9	48.4	34.4	25	43.5
1019	hypothetical_protein	7101..7544	444	0.724	33.1	43.2	31.1	25	41.3
1020	hypothetical_protein		144	0.794	34.7	50	29.2	25	32.8
1021	hypothetical_protein	5305..5448	144	0.768	32.6	43.8	29.2	25	51.8
1022	hypothetical_protein	complement(128080..128367)	288	0.707	32.3	42.7	29.2	25	44.6
1023	hypothetical_protein	complement(79542..80108)	567	0.712	33.7	36	40.2	24.9	40.2
1024	hypothetical_protein	14953..15555	603	0.738	34	47.3	29.9	24.9	37.4
1025	hypothetical_protein	complement(2268..3026)	759	0.653	34	47.8	29.2	24.9	36.8
1026	hypothetical_protein	complement(25269..25787)	519	0.735	32	47.4	23.7	24.9	45.7
1027	hypothetical_protein	51904..52350	447	0.791	34.9	48.3	31.5	24.8	40.2
1028	hypothetical_protein	8045..8323	279	0.692	36.6	44.1	40.9	24.7	41.3
1029	hypothetical_protein	3719..4168	450	0.762	34.7	49.3	30	24.7	32.5
1030	hypothetical_protein	3719..4168	450	0.762	34.7	49.3	30	24.7	32.5
1031	hypothetical_protein	complement(3577..4146)	570	0.702	33.3	46.8	28.4	24.7	47.1
1032	hypothetical_protein	complement(21196..21573)	378	0.744	33.6	41.3	34.9	24.6	41.7
1033	hypothetical_protein	113027..113404	378	0.744	33.6	41.3	34.9	24.6	41.7
1034	hypothetical_protein	113027..113404	378	0.744	33.6	41.3	34.9	24.6	41.7
1035	hypothetical_protein	46190..47083	894	0.724	36	52.7	30.9	24.5	40.4
1036	hypothetical_protein	13731..14063	333	0.732	35.1	46.8	34.2	24.3	39.9
1037	hypothetical_protein	13731..14063	333	0.732	35.1	46.8	34.2	24.3	39.9
1038	hypothetical_protein	complement(6881..7213)	333	0.741	29.4	32.4	31.5	24.3	33.4
1039	hypothetical_protein	complement(59860..60156)	297	0.758	36.4	48.5	36.4	24.2	44
1040	hypothetical_protein	10810..11106	297	0.648	31.3	36.4	33.3	24.2	38.7
1041	hypothetical_protein	126174..126719	546	0.704	33.7	47.8	29.1	24.2	47.3
1042	hypothetical_protein	complement(17640..17924)	285	0.733	35.4	56.8	25.3	24.2	53.5
1043	hypothetical_protein	complement(17640..17924)	285	0.733	35.4	56.8	25.3	24.2	53.5
1044	hypothetical_protein	9660..9833	174	0.697	28.7	31	31	24.1	42.8
1045	hypothetical_protein	complement(42979..43302)	324	0.738	32.7	47.2	26.9	24.1	34.2
1046	hypothetical_protein	59788..60111	324	0.71	34	52.8	25	24.1	45.5
1047	hypothetical_protein	7057..7344	288	0.718	32.3	42.7	30.2	24	41.6
1048	hypothetical_protein	7057..7344	288	0.718	32.3	42.7	30.2	24	41.6
1049	hypothetical_protein	complement(130560..131009)	450	0.727	32.7	46.7	27.3	24	37.7
1050	hypothetical_protein	131086..131361	276	0.809	34.8	50	30.4	23.9	34
1051	hypothetical_protein	98635..98772	138	0.749	31.2	45.7	23.9	23.9	41.2
1052	hypothetical_protein	98635..98772	138	0.749	31.2	45.7	23.9	23.9	41.2
1053	hypothetical_protein	94461..94598	138	0.751	31.2	45.7	23.9	23.9	42.6
1054	hypothetical_protein	complement(130245..130523)	279	0.726	36.9	47.3	39.8	23.7	41.2
1055	hypothetical_protein	2123..2401	279	0.723	35.8	45.2	38.7	23.7	43.7
1056	hypothetical_protein	complement(59299..59475)	177	0.786	33.9	45.8	32.2	23.7	31.3
1057	hypothetical_protein	4989..5216	228	0.694	26.3	32.9	22.4	23.7	61
1058	hypothetical_protein	complement(121099..121431)	333	0.75	35.1	47.7	34.2	23.4	38.7
1059	hypothetical_protein	10021..10251	231	0.723	31.6	42.9	28.6	23.4	42.1
1060	hypothetical_protein	complement(78370..78690)	321	0.712	33.6	52.3	25.2	23.4	45.1
1061	hypothetical_protein	52284..52604	321	0.712	33.6	52.3	25.2	23.4	45.1
1062	hypothetical_protein	117240..117524	285	0.739	35.1	57.9	24.2	23.2	53.6
1063	hypothetical_protein	complement(20974..21258)	285	0.739	35.1	57.9	24.2	23.2	53.6
1064	hypothetical_protein	48741..49091	351	0.712	29.6	41	24.8	23.1	42.3
1065	hypothetical_protein	complement(16912..17421)	510	0.705	31.4	41.8	29.4	22.9	40.5
1066	hypothetical_protein	complement(16912..17421)	510	0.705	31.4	41.8	29.4	22.9	40.5
1067	hypothetical_protein	2906..3010	105	0.657	28.6	37.1	25.7	22.9	36.9
1068	hypothetical_protein	91669..91893	225	0.67	32.4	44	30.7	22.7	58.9
1069	hypothetical_protein	91669..91893	225	0.67	32.4	44	30.7	22.7	58.9
1070	hypothetical_protein	2357..2554	198	0.787	33.8	48.5	30.3	22.7	41.9
1071	hypothetical_protein	2357..2554	198	0.787	33.8	48.5	30.3	22.7	41.9
1072	hypothetical_protein	complement(62898..63098)	201	0.676	32.8	43.3	32.8	22.4	33.1
1073	hypothetical_protein	complement(128797..128970)	174	0.731	29.9	36.2	31	22.4	44.4
1074	hypothetical_protein	3501..3674	174	0.676	28.7	32.8	31	22.4	39.8
1075	hypothetical_protein	56496..56816	321	0.717	33.3	52.3	25.2	22.4	43.8
1076	hypothetical_protein	56496..56816	321	0.717	33.3	52.3	25.2	22.4	43.8
1077	hypothetical_protein	complement(42563..42763)	201	0.731	31.8	53.7	19.4	22.4	41.3
1078	hypothetical_protein	56263..56598	336	0.722	29.2	40.2	25	22.3	41.6
1079	hypothetical_protein	48364..48579	216	0.767	32.9	45.8	30.6	22.2	34.6
1080	hypothetical_protein	complement(63108..63350)	243	0.744	29.2	35.8	29.6	22.2	45.8
1081	hypothetical_protein	complement(81883..82248)	366	0.715	29.5	41.8	24.6	22.1	42.1
1082	hypothetical_protein	52923..53303	381	0.705	29.7	42.5	24.4	22	41.7
1083	hypothetical_protein	52923..53303	381	0.705	29.7	42.5	24.4	22	41.7
1084	hypothetical_protein	10265..10552	288	0.728	31.6	42.7	30.2	21.9	43.8
1085	hypothetical_protein	complement(65542..65775)	234	0.758	33.3	38.5	39.7	21.8	45.7
1086	hypothetical_protein	117743..118252	510	0.72	30.2	40	28.8	21.8	38.5
1087	hypothetical_protein	complement(20246..20755)	510	0.72	30.2	40	28.8	21.8	38.5
1088	hypothetical_protein	complement(65788..66024)	237	0.707	35.4	40.5	44.3	21.5	38.3
1089	hypothetical_protein	91890..92213	324	0.765	32.1	48.1	26.9	21.3	30.1
1090	hypothetical_protein	91890..92213	324	0.765	32.1	48.1	26.9	21.3	30.1
1091	hypothetical_protein	101625..101837	213	0.684	24.9	28.2	25.4	21.1	29.4
1092	hypothetical_protein	42472..43152	681	0.793	31.3	47.6	25.1	21.1	40.3
1093	hypothetical_protein	47732..48277	546	0.748	29.5	39.6	28	20.9	41.5
1094	hypothetical_protein	6453..6626	174	0.724	28.7	34.5	31	20.7	41.1
1095	hypothetical_protein	6453..6626	174	0.724	28.7	34.5	31	20.7	41.1
1096	hypothetical_protein	39960..40238	279	0.768	33.7	48.4	32.3	20.4	53.5
1097	hypothetical_protein	complement(20777..20968)	192	0.742	26.6	34.4	25	20.3	38.2
1098	hypothetical_protein	100896..101207	312	0.715	36.5	57.7	31.7	20.2	41.7
1099	hypothetical_protein	complement(25088..25312)	225	0.746	29.8	40	29.3	20	41.2
1100	hypothetical_protein	12972..13349	378	0.727	33.9	47.6	34.1	19.8	35.1
1101	hypothetical_protein	4104..4391	288	0.744	30.9	42.7	30.2	19.8	41.5
1102	hypothetical_protein	complement(24102..24302)	201	0.803	32.3	55.2	22.4	19.4	44.3
1103	hypothetical_protein	46320..46676	357	0.752	32.5	45.4	32.8	19.3	43.6
1104	hypothetical_protein	complement(23713..23931)	219	0.777	33.8	50.7	31.5	19.2	37.9
1105	hypothetical_protein	complement(27049..27267)	219	0.777	33.8	50.7	31.5	19.2	37.9
1106	hypothetical_protein	complement(23713..23931)	219	0.777	33.8	50.7	31.5	19.2	37.9
1107	hypothetical_protein	97616..97912	297	0.736	36	57.6	31.3	19.2	38.4
1108	hypothetical_protein	97616..97912	297	0.736	36	57.6	31.3	19.2	38.4
1109	hypothetical_protein	50249..50581	333	0.757	35.1	46.8	39.6	18.9	41.1
1110	hypothetical_protein	complement(22811..23080)	270	0.76	31.9	42.2	34.4	18.9	41.8
1111	hypothetical_protein	14603..14890	288	0.842	37.5	52.1	41.7	18.8	35.4
1112	hypothetical_protein	complement(17443..17634)	192	0.738	25.5	32.8	25	18.8	31.9
1113	hypothetical_protein	complement(17443..17634)	192	0.738	25.5	32.8	25	18.8	31.9

1114	hypothetical_protein	complement(29226..29450)	225	0.752	28.4	40	26.7	18.7	37.4
1115	hypothetical_protein	complement(32561..32785)	225	0.752	28.4	40	26.7	18.7	37.4
1116	hypothetical_protein	complement(29226..29450)	225	0.752	28.4	40	26.7	18.7	37.4
1117	hypothetical_protein	complement(24315..25022)	708	0.804	35.7	56.8	31.8	18.6	38.6
1118	hypothetical_protein	complement(37249..37545)	297	0.724	35.4	55.6	32.3	18.2	37.2
1119	hypothetical_protein	93441..93737	297	0.724	35.4	55.6	32.3	18.2	37.2
1120	hypothetical_protein	46088..46354	267	0.806	34.8	56.2	30.3	18	30.3
1121	hypothetical_protein	complement(26949..27218)	270	0.766	31.5	42.2	34.4	17.8	41.1
1122	hypothetical_protein	complement(30285..30554)	270	0.766	31.5	42.2	34.4	17.8	41.1
1123	hypothetical_protein	complement(26949..27218)	270	0.766	31.5	42.2	34.4	17.8	41.1
1124	hypothetical_protein	107946..108215	270	0.762	31.5	42.2	34.4	17.8	44.8
1125	hypothetical_protein	complement(19573..19791)	219	0.783	33.3	49.3	32.9	17.8	37.7
1126	hypothetical_protein	111233..111451	219	0.77	33.3	50.7	31.5	17.8	34.9
1127	hypothetical_protein	complement(63586..63789)	204	0.746	27.5	32.4	32.4	17.6	30.3
1128	hypothetical_protein	46995..47186	192	0.79	25.5	32.8	26.6	17.2	40.2
1129	hypothetical_protein	11257..12135	879	0.802	36.5	46.8	45.7	17.1	38.3
1130	hypothetical_protein	complement(14478..14693)	216	0.682	33.3	33.3	50	16.7	38.1
1131	hypothetical_protein	complement(17812..18027)	216	0.682	33.3	33.3	50	16.7	38.1
1132	hypothetical_protein	complement(14478..14693)	216	0.682	33.3	33.3	50	16.7	38.1
1133	hypothetical_protein	complement(11125..11340)	216	0.698	31.5	33.3	44.4	16.7	34.5
1134	hypothetical_protein	96651..96887	237	0.76	32.9	48.1	34.2	16.5	39.7
1135	hypothetical_protein	complement(120451..120639)	189	0.705	33.9	46	39.7	15.9	38.9
1136	hypothetical_protein	complement(38299..38535)	237	0.77	32.1	46.8	34.2	15.2	37
1137	hypothetical_protein	complement(41630..41866)	237	0.77	32.1	46.8	34.2	15.2	37
1138	hypothetical_protein	complement(38299..38535)	237	0.77	32.1	46.8	34.2	15.2	37
1139	hypothetical_protein	complement(34126..34362)	237	0.777	32.1	48.1	34.2	13.9	37.6
1140	Ig domain containing protein	74791..75498	708	0.7	35.7	41.9	36	29.2	46.4
1141	Ig_domain_containing_protein	complement(19297..19977)	681	0.695	35.8	41.4	36.6	29.5	48.7
1142	Ig_domain_containing_protein	114484..115191	708	0.7	35.7	41.9	36	29.2	46.4
1143	Ig_domain_containing_protein	114484..115191	708	0.7	35.7	41.9	36	29.2	46.4
1144	Ig_domain_containing_protein	117778..118485	708	0.699	35.6	41.5	36	29.2	46
1145	Ig_domain_containing_protein	110884..111591	708	0.702	35.6	41.5	36	29.2	46
1146	Ig_domain_containing_protein	108377..109084	708	0.697	35.9	41.9	36.9	28.8	49.1
1147	kinase	28203..28787	585	0.608	40.5	46.7	31.8	43.1	58.3
1148	major head protein	5582..6475	894	0.726	39.9	51.3	38.9	29.5	37.8
1149	major head protein	11790..13196	1407	0.772	38.7	52.9	37.5	25.8	39
1150	major tail protein	8323..9285	963	0.71	41.8	52	42.1	31.5	45.8
1151	major_head_protein	5543..6451	909	0.747	38.5	49.5	38	28.1	34.6
1152	major_head_protein	51435..52841	1407	0.779	38.6	52.9	37.5	25.4	38.9
1153	major_head_protein	51435..52841	1407	0.779	38.6	52.9	37.5	25.4	38.9
1154	major_head_protein	45292..46695	1404	0.78	38.5	52.8	37.6	25.2	39.2
1155	major_head_protein	54775..56181	1407	0.78	38.5	52.9	37.5	25.2	38.2
1156	major_head_protein	complement(82330..83736)	1407	0.78	38.4	52.7	37.5	24.9	38.1
1157	major_head_protein	47253..48659	1407	0.786	38.4	52.9	37.5	24.7	39.1
1158	major_head_protein	10075..10980	906	0.794	37.5	52.6	40.4	19.5	36
1159	major_tail_protein	8299..9279	981	0.679	43.1	50.2	41.6	37.6	44.8
1160	major_tail_protein	13792..14502	711	0.739	40.9	50.6	46.8	25.3	39.1
1161	membrane protein	52..438	387	0.654	35.4	46.5	27.1	32.6	48
1162	membrane_protein	43027..43413	387	0.651	35.7	46.5	27.1	33.3	48.5
1163	membrane_protein	33563..33949	387	0.648	35.4	46.5	27.1	32.6	48.3
1164	membrane_protein	39696..40082	387	0.648	35.4	46.5	27.1	32.6	48.3
1165	membrane_protein	35523..35909	387	0.648	35.4	46.5	27.1	32.6	48.3
1166	membrane_protein	39696..40082	387	0.648	35.4	46.5	27.1	32.6	48.3
1167	membrane_protein	complement(95104..95490)	387	0.665	35.1	46.5	27.1	31.8	48.4
1168	metal-dependent hydrolase	19633..20256	624	0.635	38.9	42.8	31.2	42.8	53.6
1169	metal-dependent phosphohydrolase	complement(120540..120971)	432	0.691	32.6	46.5	24.3	27.1	56.2
1170	metal-dependent_hydrolase	20884..21456	573	0.605	39.4	44	29.3	45	50.7
1171	metal-dependent_phosphohydrolase	complement(28781..29212)	432	0.681	33.1	47.2	25	27.1	52.8
1172	metal-dependent_phosphohydrolase	complement(32116..32547)	432	0.681	33.1	47.2	25	27.1	52.8
1173	metal-dependent_phosphohydrolase	complement(28781..29212)	432	0.681	33.1	47.2	25	27.1	52.8
1174	metal-dependent_phosphohydrolase	complement(24643..25074)	432	0.695	32.4	46.5	24.3	26.4	53.9
1175	metal-dependent_phosphohydrolase	complement(22705..23136)	432	0.707	32.4	47.2	25	25	53.7
1176	metal-dependent_phosphohydrolase	105953..106384	432	0.72	31	46.5	23.6	22.9	53.2
1177	methyltransferase	31675..32130	456	0.638	36.4	44.1	32.2	32.9	50.8
1178	minor tail protein	12046..13332	1287	0.633	40.2	46.9	36.6	37.3	56.2
1179	minor_head_protein	3872..4780	909	0.556	44.3	51.5	34.3	47.2	55.2
1180	minor_tail_protein	12036..13295	1260	0.616	43.3	45.2	37.9	46.9	53.4
1181	minor_tail_protein	507..2405	1899	0.707	39.4	47.6	41.7	29.1	45.8
1182	Mom-like_DNA_modification_protein	1386..2039	654	0.704	32.9	39.4	29.8	29.4	41.9
1183	Mom-like_DNA_modification_protein	1386..2039	654	0.704	32.9	39.4	29.8	29.4	41.9
1184	Mom-like_DNA_modification_protein	129426..130079	654	0.717	32.6	39	30.3	28.4	43
1185	Mom-like_DNA_modification_protein	4319..4972	654	0.722	32.3	39.9	29.8	27.1	43.6
1186	nicotinamide phosphoribosyl transferase	61192..62985	1794	0.702	36.1	47.8	31.4	28.9	45.2
1187	nicotinamide_phosphoribosyl_transferase	94786..96570	1785	0.694	36.6	49.1	30.9	29.9	46.8
1188	nicotinamide_phosphoribosyl_transferase	104179..105972	1794	0.698	36.6	48.7	31.4	29.8	46.8
1189	nicotinamide_phosphoribosyl_transferase	96710..98494	1785	0.703	36.6	49.1	31.4	29.4	48
1190	nicotinamide_phosphoribosyl_transferase	100884..102677	1794	0.705	36.3	48.5	31.3	29.1	45.7
1191	nicotinamide_phosphoribosyl_transferase	100884..102677	1794	0.705	36.3	48.5	31.3	29.1	45.7
1192	nicotinamide_phosphoribosyl_transferase	complement(32486..34279)	1794	0.704	36.2	48.3	31.3	29.1	45.8
1193	NinI-like serine-threonine phosphatase	complement(107253..108026)	774	0.625	37.7	44.2	31.4	37.6	48.3
1194	NinI-like_serine-threonine_phosphatase	complement(12138..12911)	774	0.622	38	44.2	31.8	38	47.6
1195	NinI-like_serine-threonine_phosphatase	118900..119673	774	0.626	37.9	44.2	31.8	37.6	47.7
1196	NinI-like_serine-threonine_phosphatase	complement(15491..16264)	774	0.627	37.6	43.8	31.8	37.2	48.8
1197	NinI-like_serine-threonine_phosphatase	complement(15491..16264)	774	0.627	37.6	43.8	31.8	37.2	48.8
1198	NinI-like_serine-threonine_phosphatase	complement(18825..19598)	774	0.635	36.8	43.4	31.8	35.3	47
1199	NinI-like_serine-threonine_phosphatase	complement(10209..10982)	774	0.638	36.7	43.4	31.8	34.9	44.9
1200	NrdA-like aerobic NDP reductase large subunit	55238..56269	1032	0.679	38.5	45.9	36.9	32.6	45.7
1201	NrdA-like_aerobic_NDP_reductase_large_subunit	98221..99252	1032	0.674	38.9	46.2	37.2	33.1	46
1202	NrdA-like_aerobic_NDP_reductase_large_subunit	88801..89832	1032	0.683	38.7	46.2	37.2	32.6	45.1
1203	NrdA-like_aerobic_NDP_reductase_large_subunit	complement(39234..40265)	1032	0.683	38.7	46.2	37.2	32.6	45.1
1204	NrdA-like_aerobic_NDP_reductase_large_subunit	90721..91752	1032	0.684	38.6	46.2	37.2	32.3	44.9
1205	NrdA-like_aerobic_NDP_reductase_large_subunit	94926..95957	1032	0.694	38	45.6	37.5	30.8	44.3
1206	NrdA-like_aerobic_NDP_reductase_large_subunit	94926..95957	1032	0.694	38	45.6	37.5	30.8	44.3
1207	nucleotide kinase	27444..28220	777	0.694	37.3	44	33.6	34.4	54.1
1208	PcfJ_domain-containing_protein	106026..108527	2502	0.67	36.1	44.7	31.4	32	46.6
1209	PcfJ_domain-containing_protein	98590..101049	2460	0.675	35.9	44.4	31.7	31.6	44.7
1210	PcfJ_domain-containing_protein	102773..105232	2460	0.672	35.4	43.7	31.6	31.1	45.3
1211	PcfJ_domain-containing_protein	102773..105232	2460	0.672	35.4	43.7	31.6	31.1	45.3
1212	pentapeptide_repeat-containing_protein	complement(122332..122802)	471	0.768	33.5	44.6	31.2	24.8	36
1213	peptide methionine sulfoxide reductase	9862..10125	264	0.663	35.2	46.6	30.7	28.4	51.4
1214	peptide_methionine_sulfoxide_reductase	9875..10147	273	0.598	40.7	52.7	29.7	39.6	61
1215	phosphoesterase	complement(18959..19576)	618	0.699	33.3	43.2	28.2	28.6	43.2
1216	phosphoesterase	complement(114864..115481)	618	0.71	32.8	42.7	27.7	28.2	42.3
1217	phosphoesterase	111448..112065	618	0.711	32.8	42.7	28.2	27.7	43.7
1218	phosphoesterase	complement(26435..27052)	618	0.698	32.5	42.7	28.2	26.7	44.1
1219	phosphoesterase	complement(17029..17646)	618	0.726	33	44.2	28.6	26.2	42.1
1220	phosphoesterase	complement(23099..23716)	618	0.699	32.4	42.7	28.2	26.2	44
1221	phosphoesterase	complement(23099..23716)	618	0.699	32.4	42.7	28.2	26.2	44
1222	phosphoheptose isomerase	complement(115713..116090)	378	0.639	36.2	46	30.2	32.5	41.7
1223	phosphoheptose_isomerase	complement(17878..18255)	378	0.621	37.3	46	31	34.9	41.1
1224	phosphoheptose_isomerase	110839..111216	378	0.629	37	45.2	32.5	33.3	43.1
1225	phosphoheptose_isomerase	complement(23948..24325)	378	0.637	36.5	46.8	30.2	32.5	40
1226	phosphoheptose_isomerase	complement(27284..27661)	378	0.637	36.5	46.8	30.2	32.5	40
1227	phosphoheptose_isomerase	complement(23948..24325)	378	0.637	36.5	46.8	30.2	32.5	40
1228	phosphoheptose_isomerase	complement(19808..20185)	378	0.63	36.2	46	30.2	32.5	44.7
1229	porphyrin biosynthesis	83439..84455	1017	0.663	37.2	46.3	34.5	30.7	46.5
1230	porphyrin_biosynthesis	40260..41282	1023	0.728	38.4	49	34.3	32	44.5
1231	porphyrin_biosynthesis	complement(10337..11353)	1017	0.661	37.7	46.9	34.5	31.6	50.2
1232	porphyrin_biosynthesis	119538..120554	1017	0.662	37.4	46.6	34.5	31	48.3
1233	porphyrin_biosynthesis	117025..118041	1017	0.658	37.4	46.9	34.2	31	51
1234	porphyrin_biosynthesis	126426..127442	1017	0.663	37.2	46.3	34.5	30.7	46.6
1235	porphyrin_biosynthesis	123135..124151	1017	0.664	37.2	46.3	34.5	30.7	46.8

1236	porphyrin_biosynthesis	123135..124151	1017	0.664	37.2	46.3	34.5	30.7	46.8
1237	portal protein	2322..3914	1593	0.587	43.1	52	31.5	45.8	55.4
1238	portal protein	7822..8172	351	0.643	33.9	40.2	24.8	36.8	51.7
1239	portal protein	8189..9832	1644	0.677	37.5	49.3	33.6	29.6	44.4
1240	portal_protein	2008..3621	1614	0.594	43.2	51.5	32.7	45.4	56.3
1241	portal_protein	41333..41683	351	0.635	34.2	40.2	24.8	37.6	50.1
1242	portal_protein	47467..47817	351	0.637	34.2	40.2	24.8	37.6	50.1
1243	portal_protein	47467..47817	351	0.637	34.2	40.2	24.8	37.6	50.1
1244	portal_protein	43294..43644	351	0.634	34.2	40.2	24.8	37.6	50.6
1245	portal_protein	50798..51148	351	0.634	34.2	40.2	24.8	37.6	50.6
1246	portal_protein	complement(87357..87707)	351	0.63	34.2	40.2	24.8	37.6	51.2
1247	portal_protein	41688..43343	1656	0.684	37.1	49.3	33.2	29	45
1248	portal_protein	43661..45304	1644	0.679	37	49.1	33.2	28.8	45.1
1249	portal_protein	47834..49477	1644	0.68	37	49.3	33.2	28.5	45
1250	portal_protein	47834..49477	1644	0.68	37	49.3	33.2	28.5	45
1251	portal_protein	51165..52808	1644	0.685	36.7	49.1	33.2	27.7	44.3
1252	portal_protein	complement(85697..87340)	1644	0.689	36.7	49.3	33.2	27.6	43.6
1253	portal_protein	6892..8541	1650	0.728	37.2	50.5	34.4	26.5	45.1
1254	primase	30716..31681	966	0.692	37.6	46.6	37.6	28.6	49.1
1255	putative_deoxyuridine_5'-triphosphate_nucleotidohydrolase	86820..87467	648	0.687	39.5	48.6	41.2	28.7	41.6
1256	putative_deoxyuridine_5'-triphosphate_nucleotidohydrolase	94320..94967	648	0.697	39.2	48.6	41.2	27.8	41
1257	putative_deoxyuridine_5'-triphosphate_nucleotidohydrolase	91025..91672	648	0.692	38.7	47.2	41.2	27.8	41.2
1258	putative_deoxyuridine_5'-triphosphate_nucleotidohydrolase	91025..91672	648	0.692	38.7	47.2	41.2	27.8	41.2
1259	putative_tail_protein	71533..74937	3405	0.716	38.5	47.8	39.9	27.7	42
1260	putative_tail_protein	78982..82437	3456	0.719	38.3	47.9	39.6	27.3	41.9
1261	putative_tail_protein	75738..79142	3405	0.725	38.1	48.3	39.6	26.5	41.7
1262	putative_tail_protein	75738..79142	3405	0.725	38.1	48.3	39.6	26.5	41.7
1263	recombination endonuclease	48184..49617	1434	0.67	34.9	48.3	28.7	27.8	39.8
1264	recombination exonuclease	46586..47638	1053	0.707	35.7	49	33.3	24.8	41.9
1265	recombination_exonuclease	80152..81204	1053	0.694	36.4	49.3	33.3	26.5	44.2
1266	recombination_exonuclease	complement(47863..48915)	1053	0.705	35.9	49.3	33.6	24.8	42.7
1267	recombination_exonuclease	89572..90624	1053	0.71	35.4	48.1	33.6	24.5	41.6
1268	recombination_exonuclease	82072..83124	1053	0.706	35.7	49.3	33.3	24.5	42.3
1269	recombination_exonuclease	86277..87329	1053	0.715	35.3	49	33.3	23.6	41.5
1270	recombination_exonuclease	86277..87329	1053	0.715	35.3	49	33.3	23.6	41.5
1271	ribonucleoside diphosphate reductase small subunit	56445..57476	1032	0.701	36.3	44.2	32	32.8	45
1272	ribonucleoside_diphosphate_reductase_small_subunit	96133..97164	1032	0.701	36.4	44.2	32	33.1	44.7
1273	ribonucleoside_diphosphate_reductase_small_subunit	96133..97164	1032	0.701	36.4	44.2	32	33.1	44.7
1274	ribonucleoside_diphosphate_reductase_small_subunit	99428..100459	1032	0.701	36.3	44.2	32	32.8	45
1275	ribonucleoside_diphosphate_reductase_small_subunit	90038..91069	1032	0.702	36.1	44.2	32	32.3	44.2
1276	ribonucleoside_diphosphate_reductase_small_subunit	91958..92989	1032	0.708	35.9	44.5	32	31.4	43.7
1277	ribonucleoside_diphosphate_reductase_small_subunit	complement(37997..39028)	1032	0.715	35.6	44.2	32	30.5	43.1
1278	ribonucleotide reductase large subunit	53678..55027	1350	0.674	36.9	46.7	34.2	29.8	43.7
1279	ribonucleotide_reductase_large_subunit	93366..94715	1350	0.678	36.9	47.1	34.4	29.1	43.7
1280	ribonucleotide_reductase_large_subunit	93366..94715	1350	0.678	36.9	47.1	34.4	29.1	43.7
1281	ribonucleotide_reductase_large_subunit	87190..88590	1401	0.678	36.5	46	34.7	28.9	45.1
1282	ribonucleotide_reductase_large_subunit	96610..98010	1401	0.685	36.2	45.8	34.7	28.1	44.8
1283	ribonucleotide_reductase_large_subunit	89161..90510	1350	0.685	36.2	46.2	34.4	28	42.8
1284	ribonucleotide_reductase_large_subunit	complement(40476..41825)	1350	0.684	36.4	46.9	34.2	28	42.8
1285	ribose-phosphate pyrophosphokinase	60267..61181	915	0.696	36.9	47.9	35.7	27.2	43.1
1286	ribose-phosphate_pyrophosphokinase	103254..104168	915	0.691	37.5	47.9	36.4	28.2	45.5
1287	ribose-phosphate_pyrophosphokinase	complement(34290..35204)	915	0.705	36.6	46.2	36.1	27.5	42.6
1288	ribose-phosphate_pyrophosphokinase	93861..94775	915	0.69	37	47.9	35.7	27.5	45.3
1289	ribose-phosphate_pyrophosphokinase	99959..100873	915	0.696	36.9	47.9	35.7	27.2	43.1
1290	ribose-phosphate_pyrophosphokinase	99959..100873	915	0.696	36.9	47.9	35.7	27.2	43.1
1291	ribose-phosphate_pyrophosphokinase	95785..96699	915	0.691	37	48.5	35.4	27.2	43.4
1292	ribosome_associated_inhibitor_A;_zinc_finger_domain	complement(25611..25943)	333	0.55	41.1	41.4	40.5	41.4	54.1
1293	ribosome_associated_inhibitor_A;_zinc_finger_domain	complement(31727..31939)	213	0.609	36.6	35.2	33.8	40.8	46.1
1294	ribosome_associated_inhibitor_A;_zinc_finger_domain	complement(31727..31939)	213	0.609	36.6	35.2	33.8	40.8	46.1
1295	ribosome_associated_inhibitor_A;_zinc_finger_domain	103227..103439	213	0.617	36.2	35.2	33.8	39.4	46.1
1296	ribosome_associated_inhibitor_A;_zinc_finger_domain	complement(35061..35273)	213	0.627	35.7	35.2	33.8	38	47.4
1297	ribosome_associated_inhibitor_A;_zinc_finger_domain	complement(27586..27798)	213	0.619	35.7	36.6	32.4	38	47.3
1298	RNA ligase	complement(116527..117468)	942	0.693	35.4	47.5	31.5	27.1	43.7
1299	RNA polymerase beta subunit	19970..20557	588	0.641	40	55.1	26.5	38.3	46.8
1300	RNA polymerase sigma factor	73712..74350	639	0.654	36.5	50.2	27.2	31.9	44.9
1301	RNA_ligase	109459..110400	942	0.689	35	45.5	30.9	28.7	43.7
1302	RNA_ligase	complement(20626..21567)	942	0.704	34.9	47.5	30.9	26.4	44.3
1303	RNA_ligase	complement(18691..19632)	942	0.701	35.1	47.8	31.5	26.1	41
1304	RNA_ligase	complement(24764..25705)	942	0.719	34.2	47.1	31.5	23.9	39.9
1305	RNA_ligase	complement(28100..29041)	942	0.719	34.2	47.1	31.5	23.9	39.9
1306	RNA_ligase	complement(24764..25705)	942	0.719	34.2	47.1	31.5	23.9	39.9
1307	RNA_polymerase_beta_subunit	62944..63543	600	0.639	40	54.5	27	38.5	47.4
1308	RNA_polymerase_beta_subunit	53530..54123	594	0.644	39.9	55.1	26.3	38.4	47.8
1309	RNA_polymerase_beta_subunit	55450..56043	594	0.646	39.7	55.1	26.3	37.9	47.2
1310	RNA_polymerase_beta_subunit	complement(74932..75525)	594	0.649	39.6	55.1	26.3	37.4	46.1
1311	RNA_polymerase_beta_subunit	59661..60248	588	0.662	38.8	54.6	26.5	35.2	46.2
1312	RNA_polymerase_beta_subunit	59661..60248	588	0.662	38.8	54.6	26.5	35.2	46.2
1313	RNA_polymerase_sigma_factor	107297..107935	639	0.648	37.1	50.7	27.2	33.3	47.4
1314	RNA_polymerase_sigma_factor	complement(20558..21196)	639	0.652	36.9	50.7	27.2	32.9	49.3
1315	RNA_polymerase_sigma_factor	109789..110427	639	0.657	36.5	50.2	27.2	31.9	47.4
1316	RNA_polymerase_sigma_factor	113404..114042	639	0.658	36.3	50.2	27.2	31.5	44.7
1317	RNA_polymerase_sigma_factor	113404..114042	639	0.658	36.3	50.2	27.2	31.5	44.7
1318	RNA_polymerase_sigma_factor	116695..117336	642	0.66	36.4	50.9	27.1	31.3	45.4
1319	RNA-binding protein	complement(121570..123090)	1521	0.671	37.9	43	37.5	33.3	48.6
1320	RNA-binding_protein	complement(23733..25253)	1521	0.67	38.1	44	37.7	32.7	48.7
1321	RNA-binding_protein	complement(25669..27189)	1521	0.684	37.4	43.6	37.5	31.2	46
1322	RNA-binding_protein	103837..105357	1521	0.681	37.5	44	37.3	31.2	46.5
1323	RNA-binding_protein	complement(29809..31332)	1524	0.687	37	43.9	36.6	30.5	44.7
1324	RNA-binding_protein	complement(33143..34666)	1524	0.687	37	43.9	36.6	30.5	44.7
1325	RNA-binding_protein	complement(29809..31332)	1524	0.687	37	43.9	36.6	30.5	44.7
1326	RusA-like Holliday junction resolvase	52941..53561	621	0.694	38.2	43.5	40.1	30.9	41.7
1327	RusA-like_Holliday_junction_resolvase	complement(41940..42560)	621	0.694	38.3	44	39.6	31.4	39.8
1328	RusA-like_Holliday_junction_resolvase	88426..89046	621	0.705	37.7	43	39.6	30.4	38.3
1329	RusA-like_Holliday_junction_resolvase	92631..93251	621	0.704	37.7	43.5	39.6	30	38.6
1330	RusA-like_Holliday_junction_resolvase	92631..93251	621	0.704	37.7	43.5	39.6	30	38.6
1331	RusA-like_Holliday_junction_resolvase	86506..87126	621	0.704	37.5	43	39.6	30	38.7
1332	RusA-like_Holliday_junction_resolvase	95926..96546	621	0.714	37.4	43	39.6	29.5	38.1
1333	RuvC-like_Holliday_junction_resolvase	36856..37368	513	0.681	39.4	48.5	34.5	35.1	54.2
1334	sigma_factor	37896..38261	366	0.736	32.2	42.6	32.8	21.3	37.1
1335	single strand DNA binding protein	70700..71959	1260	0.729	41.7	61.4	38.1	25.7	40.6
1336	single_strand_DNA_binding_protein	complement(22949..24208)	1260	0.73	41.8	61.2	38.1	26.2	41.1
1337	single_strand_DNA_binding_protein	106771..108036	1266	0.722	41.9	62.1	37.4	26.1	41.4
1338	single_strand_DNA_binding_protein	110392..111651	1260	0.729	41.8	61.7	38.1	25.7	40.5
1339	single_strand_DNA_binding_protein	110392..111651	1260	0.729	41.8	61.7	38.1	25.7	40.5
1340	single_strand_DNA_binding_protein	104285..105544	1260	0.74	41.5	61.2	38.3	25	39.7
1341	single_strand_DNA_binding_protein	113686..114945	1260	0.741	41.3	61.9	37.9	24	40
1342	synaptonemal_complex_1_(SCP-1)_domain-containing_protein	105326..106114	789	0.643	35.1	43.7	30	31.6	51.5
1343	synaptonemal_complex_1_(SCP-1)_domain-containing_protein	105326..106114	789	0.643	35.1	43.7	30	31.6	51.5
1344	synaptonemal_complex_1_(SCP-1)_domain-containing_protein	108621..109409	789	0.643	35.1	43.7	30	31.6	51.8
1345	synaptonemal_complex_1_(SCP-1)_domain-containing_protein	101143..101931	789	0.643	35.1	43.7	30	31.6	52.8
1346	tail assembly chaperone	19459..19902	444	0.717	34.5	46.6	29.7	27	44.4
1347	tail associated lysin	20619..24347	3729	0.716	41.7	51.2	45.5	28.2	43.7
1348	tail fiber protein	complement(114085..114282)	198	0.689	38.9	50	33.3	33.3	37
1349	tail fiber protein	13620..14501	882	0.69	39.6	53.1	37.8	27.9	46.6
1350	tail protein	13325..14470	1146	0.635	39.9	43.5	35.6	40.6	56
1351	tail protein	14486..15325	840	0.672	42	46.1	43.6	36.4	52.2
1352	tail protein with lysin activity	24393..26780	2388	0.71	36.6	42.7	39.4	27.8	40.7
1353	tail sheath	17130..18818	1689	0.731	38.9	51	38.5	27.2	42.6
1354	tail_assembly_chaperone	complement(75593..76036)	444	0.712	35.1	46.6	29.7	29.1	44.8
1355	tail_assembly_chaperone	54939..55382	444	0.709	34.7	46.6	29.1	28.4	44.7
1356	tail_assembly_chaperone	59150..59593	444	0.718	34.7	46.6	29.7	27.7	42.7
1357	tail_assembly_chaperone	59150..59593	444	0.718	34.7	46.6	29.7	27.7	42.7

1358	tail_assembly_chaperone	62445..62888	444	0.717	34.5	46.6	29.7	27	44.4
1359	tail_assembly_chaperone	53019..53462	444	0.729	34.5	46.6	29.7	27	44.4
1360	tail_associated_lysin	63605..67333	3729	0.713	41.8	51.1	45.6	28.6	43.8
1361	tail_associated_lysin	56105..59833	3729	0.712	41.8	51.3	45.5	28.4	44.5
1362	tail_associated_lysin	54185..57913	3729	0.711	41.6	51	45.4	28.3	44.4
1363	tail_associated_lysin	complement(71154..74870)	3717	0.715	41.5	51	45.4	28.1	44.6
1364	tail_associated_lysin	60310..64038	3729	0.718	41.4	51.1	45.5	27.7	44.5
1365	tail_associated_lysin	60310..64038	3729	0.718	41.4	51.1	45.5	27.7	44.5
1366	tail_fiber_protein	complement(22320..22517)	198	0.689	38.9	50	33.3	33.3	37
1367	tail_fiber_protein	complement(18180..18377)	198	0.689	38.9	50	33.3	33.3	37
1368	tail_fiber_protein	complement(22320..22517)	198	0.689	38.9	50	33.3	33.3	37
1369	tail_fiber_protein	complement(25656..25853)	198	0.666	39.9	54.5	33.3	31.8	35.5
1370	tail_fiber_protein	112647..112844	198	0.703	38.9	53	33.3	30.3	35.7
1371	tail_fiber_protein	complement(16250..16447)	198	0.697	37.9	50	33.3	30.3	37.1
1372	tail_fiber_protein	53310..54191	882	0.685	39.6	53.1	37.4	28.2	46.7
1373	tail_fiber_protein	53310..54191	882	0.685	39.6	53.1	37.4	28.2	46.7
1374	tail_fiber_protein	47179..48060	882	0.69	39.1	52.4	37.4	27.6	46.1
1375	tail_fiber_protein	49098..49979	882	0.69	39.1	52.4	37.4	27.6	46.1
1376	tail_fiber_protein	56605..57486	882	0.692	39.5	53.1	38.1	27.2	46.6
1377	tail_fiber_protein	complement(80995..81876)	882	0.698	39.2	53.1	37.8	26.9	45.7
1378	tail_length_tape_measure_protein	15697..18609	2913	0.733	38.1	48.3	40.9	25.2	46
1379	tail_protein	13288..14451	1164	0.599	43	44.1	38.1	46.6	57.4
1380	tail_protein	14459..15295	837	0.657	42.1	43.7	43.4	39.1	51.7
1381	tail_protein	20716..23502	2787	0.743	38.9	47.1	42.4	27.2	42.9
1382	tail_protein	18614..20704	2091	0.731	38.7	49.4	41.6	25.3	44.7
1383	tail_protein_with_lysin_activity	57959..60346	2388	0.709	36.8	42.8	39.4	28.1	41.4
1384	tail_protein_with_lysin_activity	67379..69766	2388	0.709	36.9	43.1	39.4	28	43.2
1385	tail_protein_with_lysin_activity	complement(68721..71108)	2388	0.709	36.8	42.8	39.6	27.9	41.6
1386	tail_protein_with_lysin_activity	59879..62266	2388	0.708	36.7	42.8	39.6	27.8	41.5
1387	tail_protein_with_lysin_activity	64084..66471	2388	0.71	36.6	43	39.6	27.4	41.1
1388	tail_protein_with_lysin_activity	64084..66471	2388	0.71	36.6	43	39.6	27.4	41.1
1389	tail_sheath	56820..58508	1689	0.72	39.4	51.9	38.4	27.9	43.5
1390	tail_sheath	56820..58508	1689	0.72	39.4	51.9	38.4	27.9	43.5
1391	tail_sheath	60115..61803	1689	0.731	38.9	51	38.5	27.2	42.6
1392	tail_sheath	complement(76678..78366)	1689	0.73	38.7	50.8	38.4	26.8	43
1393	tail_sheath	50689..52377	1689	0.734	38.7	51.2	38.5	26.5	42.4
1394	tail_sheath	52608..54296	1689	0.731	38.7	51	38.5	26.5	42.9
1395	tail_terminator	13349..13768	420	0.729	34.3	50	33.6	19.3	44.5
1396	terminase large subunit	781..2073	1293	0.538	45.6	52	36.4	48.3	54.4
1397	terminase large subunit	1747..3300	1554	0.689	38.2	46.5	36.1	31.9	42.8
1398	terminase large subunit	1116..1397	282	0.65	36.5	43.6	34	31.9	46.7
1399	terminase small subunit	700..1116	417	0.64	38.1	50.4	28.8	35.3	49.2
1400	terminase_large_subunit	637..1959	1323	0.569	45	51.7	37.2	46.3	54.1
1401	terminase_large_subunit	36587..36868	282	0.624	37.2	43.6	34	34	46.5
1402	terminase_large_subunit	44091..44372	282	0.624	37.2	43.6	34	34	46.5
1403	terminase_large_subunit	complement(94145..94426)	282	0.628	37.2	44.7	34	33	45.3
1404	terminase_large_subunit	37218..38771	1554	0.684	38.4	46.7	36.1	32.4	42.4
1405	terminase_large_subunit	35258..36811	1554	0.689	38.2	46.5	36.1	31.9	42.8
1406	terminase_large_subunit	41391..42944	1554	0.689	38.2	46.5	36.1	31.9	42.8
1407	terminase_large_subunit	41391..42944	1554	0.689	38.2	46.5	36.1	31.9	42.8
1408	terminase_large_subunit	44722..46275	1554	0.688	38.1	46.3	36.1	31.9	43.3
1409	terminase_large_subunit	34627..34908	282	0.65	36.5	43.6	34	31.9	46.7
1410	terminase_large_subunit	40760..41041	282	0.65	36.5	43.6	34	31.9	46.7
1411	terminase_large_subunit	40760..41041	282	0.65	36.5	43.6	34	31.9	46.7
1412	terminase_large_subunit	complement(92242..93795)	1554	0.688	38	46.3	36.1	31.5	43.5
1413	terminase_large_subunit	5516..6859	1344	0.697	36.8	47.8	33.5	29	45.2
1414	terminase_small_subunit	34211..34627	417	0.64	38.4	50.4	28.8	36	50.4
1415	terminase_small_subunit	40344..40760	417	0.64	38.4	50.4	28.8	36	50.4
1416	terminase_small_subunit	40344..40760	417	0.64	38.4	50.4	28.8	36	50.4
1417	terminase_small_subunit	36171..36587	417	0.637	37.9	48.9	28.8	36	49
1418	terminase_small_subunit	43675..44091	417	0.637	37.9	48.9	28.8	36	49
1419	terminase_small_subunit	complement(94426..94842)	417	0.65	37.2	50.4	27.3	33.8	50.8
1420	terminase_small_subunit	1..504	504	0.75	35.7	52.4	29.2	25.6	41.9
1421	thioredoxin	complement(36914..37225)	312	0.61	34.9	44.2	26.9	33.7	53.1
1422	thioredoxin	93761..94072	312	0.614	34.9	45.2	26	33.7	52.9
1423	thioredoxin	97936..98247	312	0.632	34.6	46.2	26	31.7	45.4
1424	thioredoxin	101231..101542	312	0.632	34.6	46.2	26	31.7	45.4
1425	thioredoxin	97936..98247	312	0.632	34.6	46.2	26	31.7	45.4
1426	thioredoxin domain	58248..58940	693	0.605	35.1	38.5	29.9	36.8	41.2
1427	thioredoxin_domain	101499..101927	429	0.585	35.4	33.6	32.2	40.6	50.8
1428	thioredoxin_domain	92127..92534	408	0.592	34.6	32.4	31.6	39.7	51.9
1429	thioredoxin_domain	complement(36811..36939)	129	0.649	30.2	20.9	30.2	39.5	42
1430	thioredoxin_domain	98333..98632	300	0.581	35.7	37	31	39	50.6
1431	thioredoxin_domain	98333..98632	300	0.581	35.7	37	31	39	50.6
1432	thioredoxin_domain	94159..94458	300	0.58	35.7	37	31	39	52.3
1433	thioredoxin_domain	complement(36531..36830)	300	0.597	35.7	38	32	37	48.2
1434	transposase_domain-containing_protein	125020..126102	1083	0.665	38	52.9	31.3	29.6	45.1
1435	transposase_domain-containing_protein	128628..129671	1044	0.679	37.3	52.6	29.9	29.3	46.8
1436	transposase_domain-containing_protein	128628..129671	1044	0.679	37.3	52.6	29.9	29.3	46.8
1437	transposase_domain-containing_protein	131920..132990	1071	0.673	37.3	53.2	30.8	28	45.5
1438	tRNA amidotransferase	complement(117482..118018)	537	0.671	33.7	48.6	25.7	26.8	43.6
1439	tRNA splicing ligase	complement(119028..120311)	1284	0.68	37	48.4	33.2	29.4	49.5
1440	tRNA_amidotransferase	complement(19646..20182)	537	0.666	33.9	48.6	25.7	27.4	43.5
1441	tRNA_amidotransferase	complement(21581..22117)	537	0.685	33.7	48.6	25.7	26.8	43.9
1442	tRNA_amidotransferase	108909..109445	537	0.684	33.3	47.5	25.7	26.8	46.7
1443	tRNA_amidotransferase	complement(25719..26255)	537	0.689	33.7	49.7	25.7	25.7	43
1444	tRNA_amidotransferase	complement(25719..26255)	537	0.689	33.7	49.7	25.7	25.7	43
1445	tRNA_amidotransferase	complement(29055..29591)	537	0.689	33.5	49.2	25.7	25.7	43.1
1446	tRNA_splicing_ligase	complement(27265..28476)	1212	0.678	37.8	50	32.9	30.4	49.3
1447	tRNA_splicing_ligase	complement(30601..31812)	1212	0.678	37.8	50	32.9	30.4	49.3
1448	tRNA_splicing_ligase	complement(27265..28476)	1212	0.678	37.8	50	32.9	30.4	49.3
1449	tRNA_splicing_ligase	complement(23127..24338)	1212	0.684	37.8	50.2	33.2	30	50.5
1450	tRNA_splicing_ligase	106688..107899	1212	0.685	37.5	50.2	32.9	29.5	48.3
1451	tRNA_splicing_ligase	complement(21192..22403)	1212	0.682	37.5	50	33.4	29	48.9
1452	UvsX-like recombinase	72029..73273	1245	0.684	37.6	48.7	34.9	29.2	45
1453	UvsX-like_recombinase	105614..106858	1245	0.676	38.2	48.7	34.9	31.1	45.2
1454	UvsX-like_recombinase	108106..109350	1245	0.672	38	48.4	34.9	30.6	45.1
1455	UvsX-like_recombinase	complement(21635..22879)	1245	0.677	37.8	48.4	34.9	30.1	45.5
1456	UvsX-like_recombinase	115015..116259	1245	0.68	37.7	48.4	34.9	29.6	45.3
1457	UvsX-like_recombinase	111721..112965	1245	0.684	37.6	48.7	34.9	29.2	45
1458	UvsX-like_recombinase	111721..112965	1245	0.684	37.6	48.7	34.9	29.2	45
1459	virion structural protein	3369..4208	840	0.654	36.4	46.1	31.4	31.8	46.2
1460	virion structural protein	88350..88817	468	0.655	34.6	46.2	27.6	30.1	50.9
1461	virion structural protein	31412..35341	3930	0.684	37.1	44.6	38.2	28.6	43.6
1462	virion structural protein	14519..15337	819	0.664	36.5	43.2	38.1	28.2	44.4
1463	virion structural protein	28368..29081	714	0.701	39.6	52.5	41.6	24.8	43.7
1464	virion structural protein	18937..19308	372	0.773	35.8	46	37.1	24.2	39.2
1465	virion structural protein	35458..35979	522	0.768	39.3	52.3	44.3	21.3	36.2
1466	virion_structural_protein	complement(91334..92173)	840	0.647	36.5	46.1	30.7	32.9	43.4
1467	virion_structural_protein	38840..39679	840	0.651	36.5	46.1	31.4	32.1	46.1
1468	virion_structural_protein	36880..37719	840	0.654	36.4	46.1	31.4	31.8	46.2
1469	virion_structural_protein	46344..47183	840	0.655	36.4	46.4	31.4	31.4	46.1
1470	virion_structural_protein	43013..43852	840	0.654	36.2	45.7	31.4	31.4	46.4
1471	virion_structural_protein	43013..43852	840	0.654	36.2	45.7	31.4	31.4	46.4
1472	virion_structural_protein	131338..131805	468	0.646	34.8	45.5	27.6	31.4	50.9
1473	virion_structural_protein	124438..124905	468	0.649	34.4	44.9	27.6	30.8	51
1474	virion_structural_protein	complement(5385..5852)	468	0.643	34.2	44.2	27.6	30.8	47.7
1475	virion_structural_protein	128046..128513	468	0.655	34.6	46.2	27.6	30.1	50.9
1476	virion_structural_protein	128046..128513	468	0.655	34.6	46.2	27.6	30.1	50.9
1477	virion_structural_protein	54209..55027	819	0.66	37.1	43.6	38.5	29.3	44.4
1478	virion_structural_protein	54209..55027	819	0.66	37.1	43.6	38.5	29.3	44.4
1479	virion_structural_protein	48078..48896	819	0.662	36.8	42.9	38.5	28.9	45.2

1480	virion_structural_protein	49997..50815	819	0.662	36.8	42.9	38.5	28.9	45.2
1481	virion_structural_protein	74398..78327	3930	0.683	37.3	44.7	38.2	28.9	44.2
1482	virion_structural_protein	complement(80159..80977)	819	0.658	36.9	43.6	38.1	28.9	45.3
1483	virion_structural_protein	64978..68907	3930	0.689	37.2	44.6	38.2	28.8	43.8
1484	virion_structural_protein	66898..70827	3930	0.69	37.2	44.6	38.4	28.5	43.5
1485	virion_structural_protein	complement(60160..64089)	3930	0.692	37.1	44.7	38.2	28.2	43.7
1486	virion_structural_protein	57504..58322	819	0.666	36.5	43.2	38.1	28.2	44.1
1487	virion_structural_protein	71103..75032	3930	0.692	37.1	44.7	38.5	28.1	43.6
1488	virion_structural_protein	71103..75032	3930	0.692	37.1	44.7	38.5	28.1	43.6
1489	virion_structural_protein	122514..122981	468	0.669	33.3	44.9	27.6	27.6	47.9
1490	virion_structural_protein	71354..72067	714	0.683	40.5	52.5	41.6	27.3	45.4
1491	virion_structural_protein	68059..68772	714	0.686	39.9	51.7	41.6	26.5	42.9
1492	virion_structural_protein	68059..68772	714	0.686	39.9	51.7	41.6	26.5	42.9
1493	virion_structural_protein	2418..3857	1440	0.711	38.5	49	41	25.6	48
1494	virion_structural_protein	58576..58998	423	0.765	36.4	46.1	37.6	25.5	40.7
1495	virion_structural_protein	58576..58998	423	0.765	36.4	46.1	37.6	25.5	40.7
1496	virion_structural_protein	61859..62293	435	0.765	35.9	45.5	36.6	25.5	41.6
1497	virion_structural_protein	complement(66420..67133)	714	0.699	39.8	52.5	41.6	25.2	44.3
1498	virion_structural_protein	63854..64567	714	0.701	39.6	52.1	41.6	25.2	44.2
1499	virion_structural_protein	61934..62647	714	0.701	39.6	52.5	41.6	24.8	43.7
1500	virion_structural_protein	52445..52867	423	0.771	35.9	45.4	37.6	24.8	40.5
1501	virion_structural_protein	complement(76188..76610)	423	0.771	35.9	45.4	37.6	24.8	40.5
1502	virion_structural_protein	54364..54786	423	0.771	35.9	45.4	37.6	24.8	40.5
1503	virion_structural_protein	78444..78965	522	0.747	39.3	50.6	44.3	23	40.6
1504	virion_structural_protein	70944..71465	522	0.756	39.5	52.3	44.3	21.8	36.5
1505	virion_structural_protein	69024..69545	522	0.757	39.5	52.3	44.3	21.8	36.7
1506	virion_structural_protein	complement(59522..60043)	522	0.759	39.3	52.3	44.3	21.3	36.5
1507	virion_structural_protein	75149..75670	522	0.759	39.3	52.3	44.3	21.3	36.5
1508	virion_structural_protein	75149..75670	522	0.759	39.3	52.3	44.3	21.3	36.5
1509	XkdX_domain-containing_protein	81143..81280	138	0.728	33.3	41.3	32.6	26.1	41.1
1510	XkdX_domain-containing_protein	76938..77075	138	0.728	33.3	41.3	32.6	26.1	41.1
1511	XkdX_domain-containing_protein	84438..84575	138	0.728	33.3	41.3	32.6	26.1	41.1
1512	XkdX_domain-containing_protein	81143..81280	138	0.728	33.3	41.3	32.6	26.1	41.1
1513	YopX-like_protein	11726..12130	405	0.614	40.7	51.1	31.9	39.3	46.3
1514	YopX-like_protein	5873..5977	105	0.611	43.8	42.9	51.4	37.1	60.3
1515	YopX-like_protein	8519..8923	405	0.636	39.5	51.1	31.1	36.3	46.5
1516	YopX-like_protein	8519..8923	405	0.636	39.5	51.1	31.1	36.3	46.5

Table S6. Pearson correlation matrix of gene length and codon usage variables determined for the Listeria temperate phages

	Gene length (nt)	CAI	%GC	%GC1	%GC2	%GC3	Nc
Gene size	1	0.127635205	0.19417064	0.160823947	0.291766376	-0.06819659	0.131537255
CAI	0.127635205	1	-0.380393759	-0.023074247	0.058244738	-0.771116391	-0.34337243
%GC	0.19417064	-0.380393759	1	0.648315578	0.674092117	0.637598991	0.303236176
%GC1	0.160823947	-0.023074247	0.648315578	1	0.183155486	0.096478078	0.120393647
%GC2	0.291766376	0.058244738	0.674092117	0.183155486	1	0.141452884	0.148098888
%GC3	-0.06819659	-0.771116391	0.637598991	0.096478078	0.141452884	1	0.323813823
Nc	0.131537255	-0.34337243	0.303236176	0.120393647	0.148098888	0.323813823	1

Table S7. Pearson correlation matrix of gene length and codon usage variables determined for the *Listeria* virulent phages

	Gene length (nt)	CAI	%GC	%GC1	%GC2	%GC3	Nc
Gene size	1	0.075015398	0.287901448	0.19593678	0.339159338	-0.056126326	0.023859624
CAI	0.075015398	1	-0.364786803	0.084304832	0.109301452	-0.762573135	-0.424095709
%GC	0.287901448	-0.364786803	1	0.550245332	0.519047521	0.56105733	0.267889214
%GC1	0.19593678	0.084304832	0.550245332	1	-0.053041098	-0.036302343	0.015767212
%GC2	0.339159338	0.109301452	0.519047521	-0.053041098	1	-0.080731293	-0.062486315
%GC3	-0.056126326	-0.762573135	0.56105733	-0.036302343	-0.080731293	1	0.469281875
Nc	0.023859624	-0.424095709	0.267889214	0.015767212	-0.062486315	0.469281875	1
